# Multicomponent Green Synthesis Involving Aryl Aldehydes and Trapped Enols: Dimerization over Cyclization

**DOI:** 10.1021/acsomega.5c07008

**Published:** 2026-01-22

**Authors:** Sarah K. Zingales, McKenna Gibson, Julio Tapia-Hernandez, Kendall Jenkins, Mitchel Munzing, Grace Dickerson, Selena Speikers, David J. Frazer, Clifford W. Padgett, Michael T. Wentzel

**Affiliations:** † Department of Chemical and Environmental Sciences, United States Coast Guard Academy, 31 Mohegan Ave Pkwy, New London, Connecticut 06320, United States; ‡ Department of Chemistry, 8515 University of Saint Joseph, 1678 Asylum Ave, West Hartford, Connecticut 06117, United States; § Department of Chemistry, 5517 Augsburg University, 2211 Riverside Ave, Minneapolis, Minnesota 55454, United States; ∥ Department of Chemistry and Biochemistry, 7604 Georgia Southern University, 11935 Abercorn St, Savannah, Georgia 31419, United States

## Abstract

This report serves two main purposes: (1) to correct the literature in the area of multicomponent synthesis involving aryl aldehydes and trapped enols 4-hydroxycoumarin **1** or 4-hydroxy-6-methyl-2-pyrone **2** and (2) to fully characterize the dimerization products bis-coumarins **3** and bis-pyrones **4**. There have been many reports of cyclizations occurring with these species and various catalysts; however, many products have been mis-characterized and are, in fact, dimers. We successfully synthesized these dimers using a green, one-pot reaction in water that avoids hazardous organic solvents, uses a catalytic amount of acid, does not require chromatography for purification, and has strong green chemistry metrics. Our simplified procedure resulted in high yields of dimers ranging from 24 to 96% including the first report of a meta-substituted bis-pyrone **4i**. Herein, we report a green method for their synthesis, along with their photophysical properties, full characterization, and potential as AChE inhibitors for anti-Alzheimer’s therapy.

## Graphical abstract

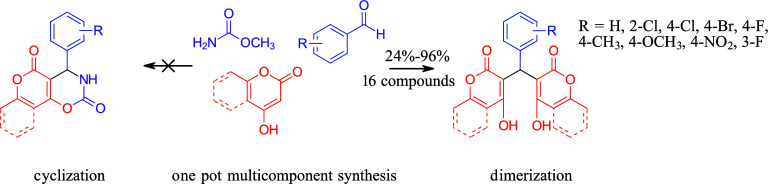

## Introduction

Creating green, efficient methods to access compounds containing the privileged structures 4-coumarinol **1** and 4-hydroxy-6-methyl-2-pyrone **2** (Figure 1), is important due to their existence as pharmacophores that are found in natural products (ammoresinol,^1^ fusapyrone,^2^ arisugacin A,^3^ scopoletin,^4^), approved drugs (warfarin,^5^ tipranavir^6^), and other biologically active compounds (anti-Alzheimer’s,^7,8^ anticoagulant,^9^ antitubercular,^10^ antimitotic,^11^ anticancer,^12^ antimicrobial,^13^ and antiviral^14,15^ ). Our lab is specifically interested in synthesis of bis-coumarins **3** and bis-pyrones **4**. While bis-coumarins **3** are well established in the literature, displaying a variety of biological activities, including antibacterial,^16^ anticoagulant,^17,18^ antiviral,^19,20^ antiparasitic,^21,22^ and antitumor,^23^ bis-pyrones **4** have been less extensively studied.

**Figure 1:**
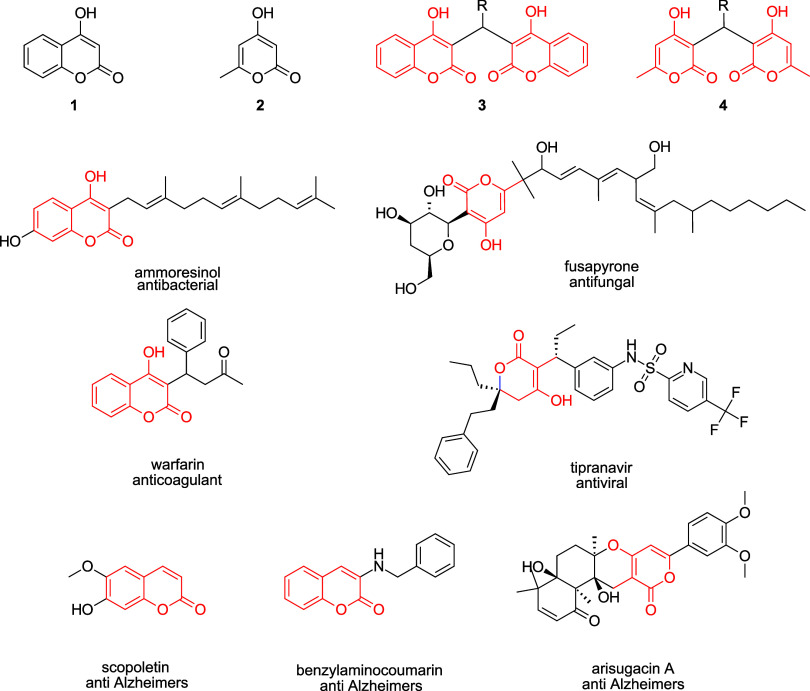
4-Coumarinol **1** and 4-hydroxy-6-methyl-2*H*-pyran-2-one **2** are scaffolds (highlighted in red) that occur in natural products (such as the antibacterial compound ammoresinol and the antifungal compound fusapyrone), approved drugs (such as the anticoagulant warfarin and antiviral tipranavir), compounds that have anti-Alzheimer’s activity (scopoletin, benzylaminocoumarin, and arisugacin A), bis-coumarins **3,** and bis-pyrones **4**.

The bis-pyrones **4** have been synthesized by various groups from 4-hydroxy-6-methyl-2-pyrone **2**; however, the currently published syntheses have significant drawbacks (Figure 2, Table S1), including use of (A) stoichiometric reagents, (B) strong acids, (C) reflux conditions, (D) heavy metals, (E) organic solvents, (F) chlorinated solvents, and/or required purification by (G) column chromatography or (H) recrystallization from organic solvents.

**Figure 2:**
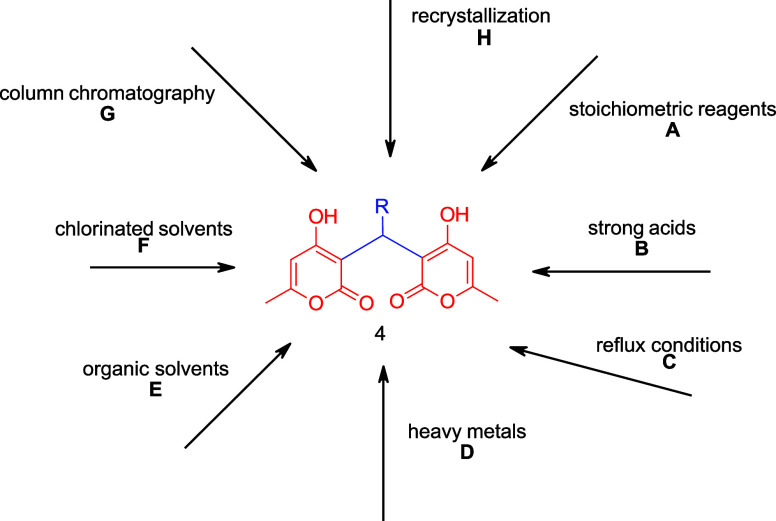
Current published synthetic methods to bis-pyrones **4**.

## Results and Discussion

Recently, our research group has been interested in developing a green version of a Biginelli-like reaction to synthesize oxazinones from trapped enols (such as **1** or **2**), aryl aldehyde, methyl carbamate, and TsOH catalyst. However, with these conditions, no cyclization products were observed. Rather, rapid dimerization occurred, yielding bis-coumarins **3** and bis-pyrones **4** in ∼50% yield. We removed methyl carbamate from the reaction to confirm that it was not needed for the dimerization to proceed. Following successful dimer formation without methyl carbamate, we then increased the ratio of aryl aldehyde:trapped enol to 1:2 to reflect the mole ratio of the dimer product and were able to synthesize a library of dimers in good (72–96%) yield for the *ortho/para* substituted aldehydes **3a**–**h**, **4a**–**c**, **4e**–**h** and poor yield (24%) for the novel *meta*-fluoro substituted aldehyde **4i** (Scheme 1).

**Scheme 1:**
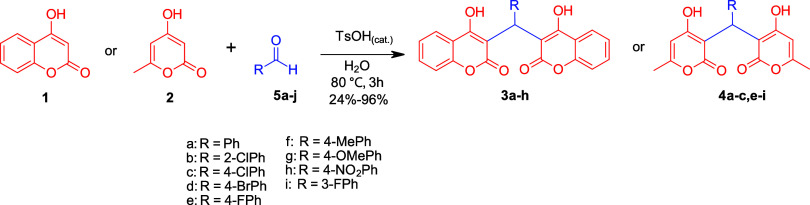
Optimized Synthesis of Dimers Using TsOH in Water at 80 °C for 3 h

Similar dimerizations have been observed by other groups seeking to perform the Biginelli reaction on trapped enols. Harichandran et al.^24^ isolated coumarin dimers when attempting the traditional Biginelli reaction with 4-hydroxycoumarin, benzaldehyde, urea, and Amberlite IRA-400 Cl resin. Koumpoura et al.^25^ isolated naphthoquinone dimers with 2-hydroxy-1,4-naphthoquinone (lawsone), 4-chlorobenzaldehyde, and urea with various acid catalysts. Mechanistically, these dimerizations occur via the tandem Knoevenagel-Michael reaction (Scheme 2). Our calculations indicate that in this acid-catalyzed system with pyrone, the transition state energy of the first step of the Knovenagel reaction is quite facile (−58 kJ/mol). Thus, using these trapped enols in other multicomponent reactions may be a challenge due to the rapid dimerization and may explain some of the discrepancies in the literature.

**Scheme 2:**
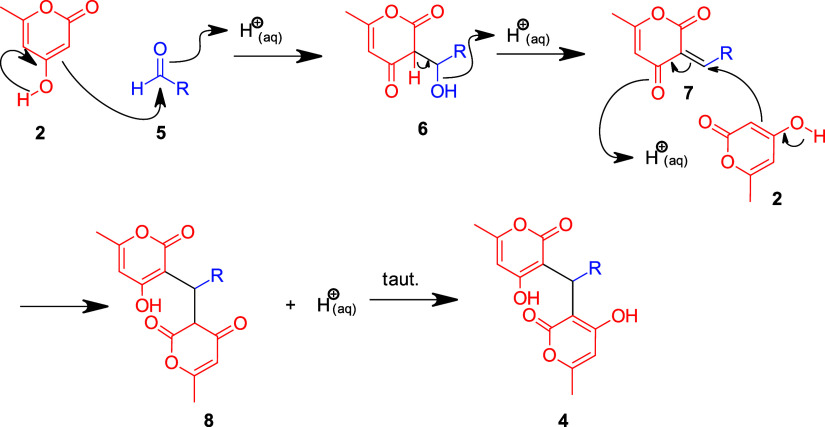
Proposed Mechanism of the Acid-Catalyzed Tandem Knoevenagel-Michael Reaction to Form the Dimers

Our optimized synthesis of dimers is a green method. It uses water as solvent, an organic acid catalyst (TsOH), and requires only filtration for isolation hence no extraction or chromatography. A number of green chemistry metrics quantifies the “greenness” of the process and a representative set of calculations was done. This multicomponent reaction is atom economical (96%) as it allows for the synthesis of these complex dimers from simple starting materials by providing the most waste-free and environmentally benign combinations. The reaction mass efficiency (88%) indicates that it is both atom economical but also high yielding. The E-factor^26^ (44.5) is relatively low for small molecule synthesis which often range from 50 to 400. Finally, the process mass intensity (45.6) reflects a highly environmentally process when these often range 170–360 in industrial processes.^27^

While the bis-coumarins **3** are well established in the literature, and have been synthesized in a variety of methods, the characterization data is not extensive. Only 13% of the literature references for the bis-coumarins (SciFinder structure search 7/1/2025) included NMR data, and few have full spectra included in their Supporting Information. Reports of the synthesis of bis-pyrones **4** exist, but they are sparse, and there are multiple instances where the characterization data is missing, incomplete, or incorrect. Table 1 summarizes the substrate scope, yield, and new characterization data that we have generated for the bis-pyrones **4** and full details are available in the Supporting Information including spectra. Of note, this is the first report of **4i**.

**Table 1:** Bis-pyrone Dimer Characterization

product	R	new characterization data	yield
**4a**	Ph	^1^H and ^13^C NMR at full scale, FTIR, HRMS	84–90%
**4b**	2-Cl-Ph	HRMS	76–84%
**4c**	4-Cl-Ph	^1^H and ^13^C NMR at full scale, FTIR, HRMS	80–84%
**4e**	4-F-Ph	^1^H and ^13^C NMR, FTIR, HRMS	87–91%
**4f**	4-CH_3_–Ph	^1^H and ^13^C NMR, FTIR, HRMS	74–86%
**4g**	4-CH_3_O-Ph	^1^H and ^13^C NMR at full scale, FTIR, HRMS	79–83%
**4h**	4-NO_2_–Ph	^1^H and ^13^C NMR, FTIR, HRMS	72–86%
**4i**	3-F-Ph	^1^H and ^13^C NMR, FTIR, HRMS	24%

Our method for bis-pyrone **4** synthesis has many advantages over published ones in that it does not use heavy metal catalysts,^28,29^ or stoichiometric or excess catalysts.^30–34^ It also does not require column chromatography^30^ or use of organic solvents for purification.^29,31–34^ In fact, it does not require any organic solvents for workup or in the reaction.^35–37^ Our yields are generally higher than the others reported for the *ortho/para*-substituted bis-pyrones **4b**–**h** (72–96%) and this is the first report of a *meta*-substituted bis-pyrone **4i** (24%).

The photophysical properties of bis-coumarins **3** have been studied,^24^ but those of bis-pyrones **4** have not. Thus, the synthesized compounds **4a**–**4i** were dissolved in acetonitrile (ACN) and *N,N-*dimethylformamide (DMF) at 10^–5^ M and their UV–visible absorption spectra were recorded. Based on the λ_max_ the absorption spectra, emission spectra were recorded. The absorption maxima of the compounds for **4a**–**4i** were found to be in a range of 280–316 nm in ACN and a narrower range of 281–293 nm in DMF (Figures S1–S16). The maximum emission wavelength (λ_em_) exhibited by these compounds ranges from 348 to 460 nm in ACN when they are excited at their absorption (λ_max_) and 338 to 400 nm when in DMF (Figures S17–S22). The photophysical characteristics such as absorption (λ_max_), emission (λ_max_), and Stokes shift (Dυ̅) are given in Table 2. Generally, the emission wavelengths were observed at a lower wavelength in nonpolar solvent (ACN) compared to in polar solvent (DMF). The fluorine-containing compounds **4e** and **4i** had the highest wavelength for absorption λ_max_ and the lowest Stokes shifts. Contrary to what has been reported with bis-coumarins **3,** ^24,38^ the largest Stokes shifts for bis-pyrones **4** were seen in ACN. The solvatochromism of these compounds can likely be attributed to the differences in hydrogen bonding of the excited state of the molecules and the solvent.^38^

**Table 2:** Photophysical Properties of Bis-pyrones **4a**–**4i**

compound	solvent	Abs λ_max_ (nm)	emission λ_max_ (nm)	Stokes shift (Dυ̅ (cm^–1^)
**4a**	ACN	288	382	8397
	DMF	292	390	8550
**4b**	ACN	290	385	8509
	DMF	292	390	8580
**4c**	ACN	285	445	12,616
	DMF	282	385	9550
**4e**	ACN	316	380	5330
	DMF	285	386	9181
**4f**	ACN	280	430	12,458
	DMF	281	400	10,590
**4g**	ACN	291	460	12,684
	DMF	283	390	9630
**4h**	ACN	283	400	10,336
	DMF	282	395	10,140
**4i**	ACN	309	348	3627
	DMF	290	338	4900

Another interesting structural feature is that both the pyrone and coumarin dimers display atropisomerism as observed in the NMR spectra in different solvents and temperatures. The atropisomerism of the bis-coumarins is known,^39^ and the effects are mostly acutely seen in the OH peaks. In the ^1^H NMR in CDCl_3_, two OH peaks at ∼11 ppm are typically observed, but in DMSO-*d*_6_ those peaks collapse to one broad OH peak instead. The peaks for the coumarin rings also coalesce into the predicted splitting pattern in DMSO-*d*_6_, rather than the extra splitting seen in CDCl_3_ in both in the ^1^H and ^13^C NMRs. Figure 3 (right) shows the solvent effects for **3c.**

**Figure 3:**
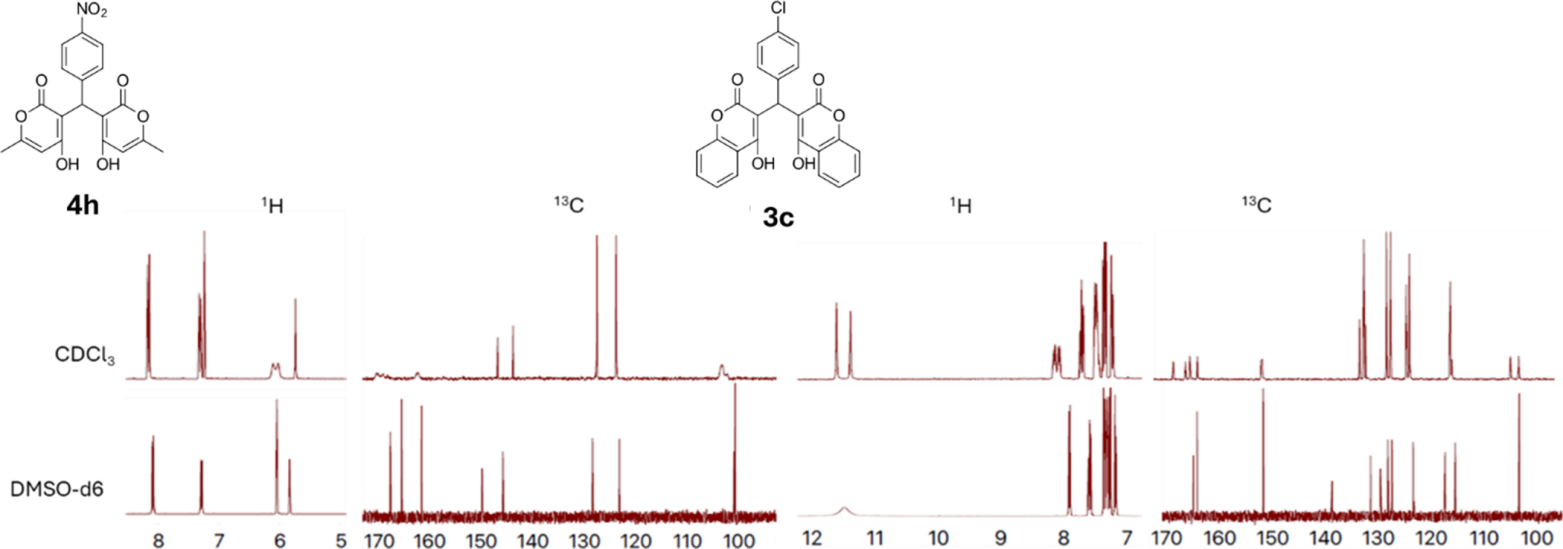
Solvent effects on the NMR spectra of **4h** and **3c**.

For the previously unreported atropisomerism of the bis-pyrones, there is generally a peak-broadening of the alkene CHs at ∼6 ppm in the ^1^H NMR. For the ones with electron-withdrawing substituents (**4c**, **4e**, **4h**), this peak is split into two in CDCl_3_, but is not baseline resolved. In the ^13^C NMR, we observed similar peak broadening for peaks of the pyrone rings. However, in DMSO-*d*_6_ (**4h**), these peaks narrow and/or collapse back into one peak in both the ^1^H and ^13^C NMRs. Figure 3 (left) shows the differences in these solvent effects for **4h** in both ^1^H and ^13^C. Additional NMR spectra for the atropisomers are found in the Supporting Information (Figures S23–S29). The existence of these atropisomers may be part of the reason that others in the literature have struggled to accurately characterize these compounds.

In order to further determine the structural difference between the atropisomers, the lowest energy conformation state of **4h** was determined by Spartan Student Edition, using MMF conformational analysis around the pyrone C1–C2-methine C3-arylC4 torsional angle (Figure 4 and Table S2). The lowest energy conformation (relative energy = −35 kJ/mol) has the two pyrone rings hydrogen bonded from one OH to the C=O of the other ring. The lowest energy conformation is asymmetrical with regards to the two rings and would explain the two sets of peaks for the rings in CDCl_3_ where there is no possibility of hydrogen bonding with the solvent. This proposed structure matches the hydrogen bonding seen in the X-ray crystal structure^23^ for bis-coumarins. We were able to crystallize **4h** and obtain a single crystal X-ray structure to confirm a strong interaction between the C=O and OH of 1.727 Å (Figure 4 and CIF file in SI). In DMSO-*d*_6_, however, solvation effects would allow for hydrogen bonding to the solvent, favoring the higher energy local minima and making the two rings indistinct via NMR.

**Figure 4:**
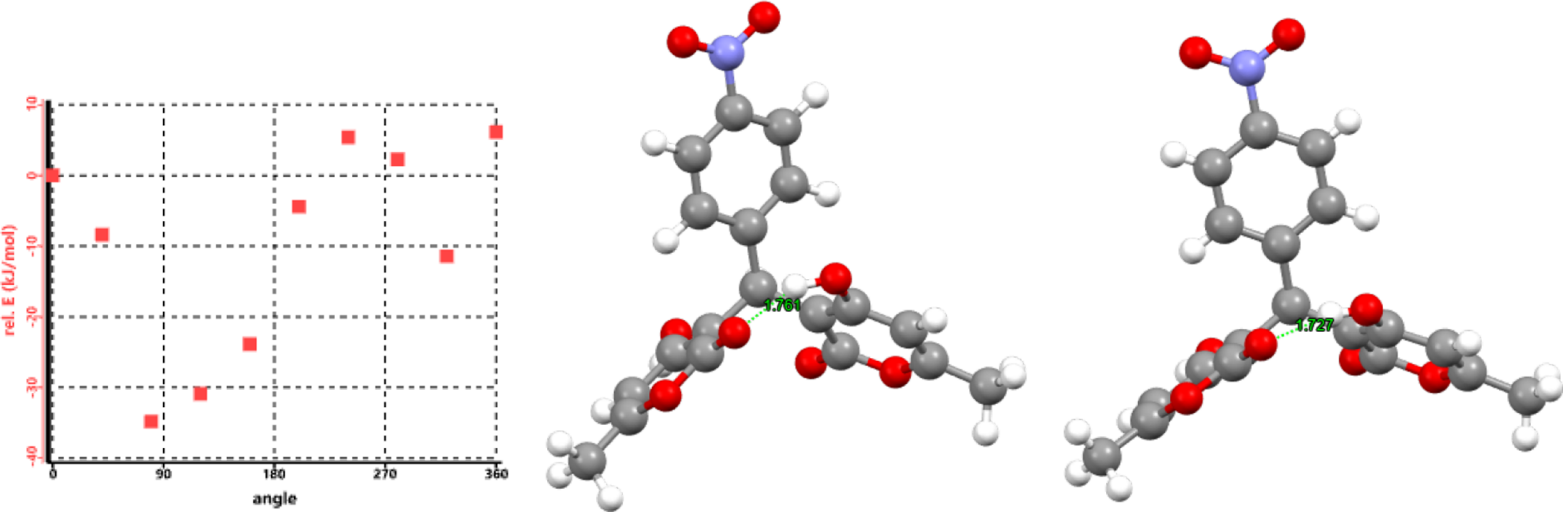
Structural analysis of **4h**. The graph on the left shows molecular modeling of the rotation of one pyrone ring to the methine CH as torsional angle versus relative energy. The predicted lowest energy conformation (80°) is shown in the center and the X-ray crystal structure is on the right, both with the hydrogen bond from C=O to OH displayed in green lines (1.761 and 1.727 Å, respectively).

Next, variable temperature ^1^H NMR was conducted on **4h** in CDCl_3_ (Figure 5). At 50 °C the OH and pyrone peaks began to coalesce toward the appearance of the DMSO-*d*_6_ spectrum. This confirms our hypothesis that the effects seen in NMR are from conformations, as the energy in the system increased enough to allow rotation around the pyrone-methine CH bond.

**Figure 5:**
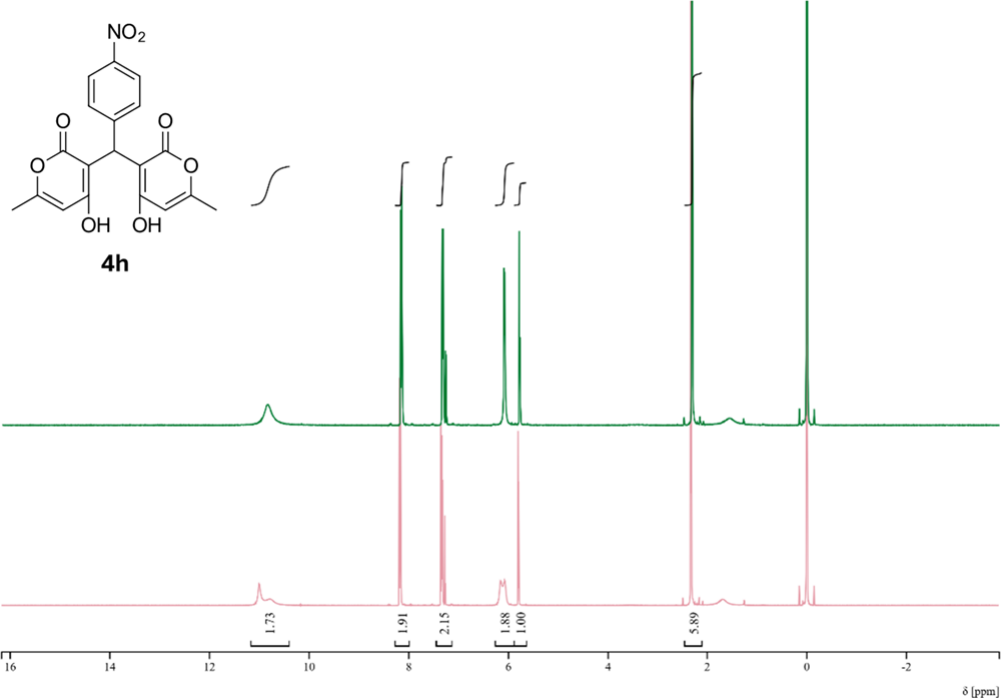
^1^H VT-NMR of **4h** in CDCl_3_. Room temperature (red) and 50 °C (green).

We also tested a subset of the synthesized dimers (**3c**, **3e**, **4c**, **4f**) for acetylcholinesterase (AchE) inhibition activity, as pyrone- and coumarin-containing compounds have demonstrated anti-Alzheimer’s activity (Figure 1). Unfortunately, the tested compounds had low inhibition (0–12%) at the 10^–4^ M concentration tested (Table 3). The coumarin dimers had slightly higher inhibition than the pyrone dimers, but this subset did not have high activity.

**Table 3:** Acetylcholinesterase Inhibition Activity

	% inhibition
control 1 (no enzyme, *n* = 2)	100%
control 2 (no inhibitor, *n* = 2))	0%
physostigmine (known AChE inhibitor, *n* = 3)	100%
**4c** (*n* = 2)	0%
**4f** (*n* = 2)	4%
**3c** (*n* = 4)	6%
**3e** (*n* = 2)	12%

## Conclusions

In summary, a highly efficient, environmentally conscious dimerization reaction was described. A number of reactions involving 4-hydroxycoumarin **1**/4-hydroxy-6-methyl-2-pyrone **2** and nine aryl aldehydes were performed in water with benign catalysts resulting in high yields of bis-coumarins **3** and bis-pyrones **4**. Through this efficient procedure, a diverse molecular library was created, a green synthesis was optimized, and the full characterization including photophysical properties was reported. The atropisomerism of these dimers was explored via molecular modeling, variable temperature NMR, and variable solvent NMR. These compounds and their derivatives are now more accessible and their important various biological activities will be further developed and advanced.

## Methods

### Synthesis

All starting materials were received from commercial vendors and used as-is without any further purification. Products obtained via this procedure were characterized using ^1^H and ^13^C NMR spectroscopy, IR spectroscopy, high-resolution mass spectrometry, and melting point.

In a typical procedure, arylaldehyde (1 mmol), trapped enol (4-hydroxycoumarin or 4-hydroxy-6-methyl-2-pyrone, 2 mmol), and para-toluenesulfonic acid (PTSA, 0.1 mmol) were suspended in DI water. The reaction was heated for three hours at 80 °C. The solid was vacuum filtered and washed with DI water to yield the white solid products.

### AChe Inhibition Assay

Method followed from Sigma-Aldrich MAK 324 Acetylcholinesterase Inhibitor Screening Kit. Briefly, for the no-enzyme control, assay buffer (45 μL) and ultrapure water (5 μL) were added, and after 15 min reaction mix (150 μL) was added. For the no-inhibitor control, prepped AChE solution (45 μL) and ultrapure water (5 μL) were added, and after 15 min reaction mix (150 μL) was added. For the positive control (known inhibitor phystostigmine), prepped AChE solution (45 μL) and test solution (5 μL of 10^–2^ M) were added, and after 15 min reaction mix (150 μL) was added. For the test wells, prepped AChE solution (45 μL) and test solution (5 μL of 10^–2^ M) were added, and after 15 min reaction mix (150 μL) was added. Absorbance was read at 412 nm and control 2 was set to 100% activity/0% inhibition. Activity/inhibition was reported as averages of number of runs.

## Experimental Section


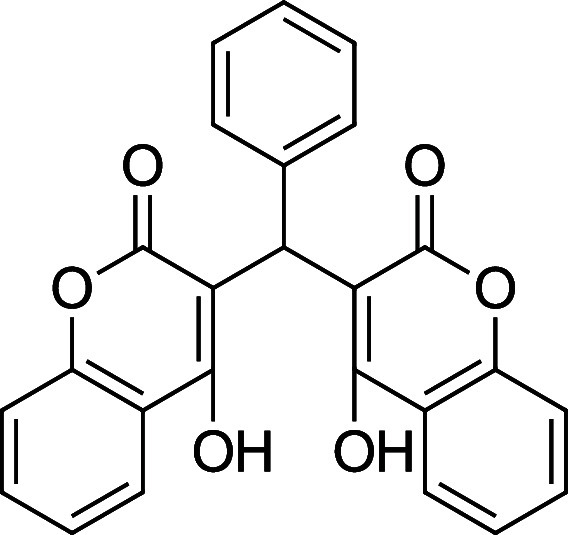


### **3a**. 3,3′-(Phenylmethylene)­bis­(4-hydroxy-2H-chromen-2-one)

Average yield: 0.380 g (90–94%);^1^H NMR (300 MHz, CDCl_3_) δ 11.54 (s, 1H), 11.31 (s, 1H), 8.08–7.99 (m, 2H), 7.63 (ddd, *J* = 8.5, 7.2, 1.7 Hz, 2H), 7.43–7.21 (m, 9H), 6.10 (s, 1H) ppm; Mp: 224–226 °C; ^13^C NMR (75 MHz, CDCl_3_) δ 169.3, 166.9, 165.8, 164.6, 152.5, 152.3, 135.2, 132.9, 128.7, 126.9, 126.5, 124.9, 124.4, 116.9, 116.7, 116.5, 105.6, 103.9, 36.2 ppm; HRMS (ESI^+^) *m*/*z*: [M + Na]^+^ calcd 435.0839, found 435.0823. IR (neat): 3070 (OH), 1652 (C=O), 1602 (C=C) cm^–1^.
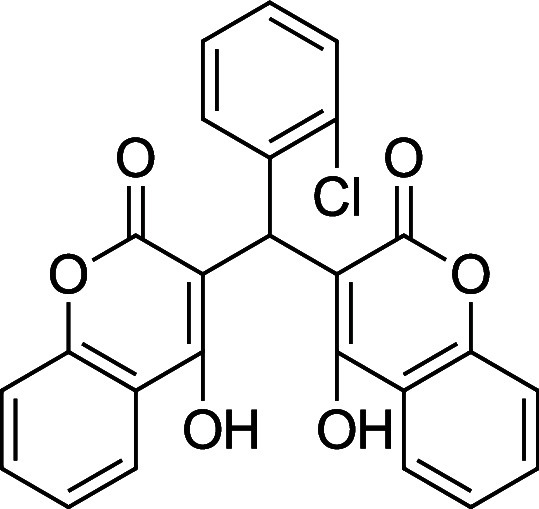


### **3b**. 3,3′-((2-Chlorophenyl)­methylene) bis­(4-hydroxy-2H-chromen-2-one)

Average yield: 0.407 g (89–93%);^1^H NMR (300 MHz, CDCl_3_) δ 11.54 (s, 1H), 11.31­(s, 1H), 8.03 (dd, *J* = 8.7, 7.2 Hz, 2H), 7.64 (td, *J* = 7.8, 1.5 Hz, 2H), 7.44–7.36 (m, 6H), 7.1 (dd, *J* = 8.7, 1.2 Hz, 2H), 6.01 (s,1H) ppm; Mp: 201–203 °C; ^13^C NMR (75 MHz, CDCl_3_) δ 168.8, 167.2, 165.1, 164.6, 152.4, 152.3, 133.5, 132.9, 130.9, 129.3, 128.7, 126.8, 125.0, 124.5, 116.7,105.7, 104.4, 35.7 ppm; HRMS (ESI^–^) *m*/*z*: [M-1]^−^ calcd 445.0484, found 445.0489. IR (neat): 3064 (OH), 1647 (C=O), 1561 (C=C), 737 (C–Cl) cm^–1^.
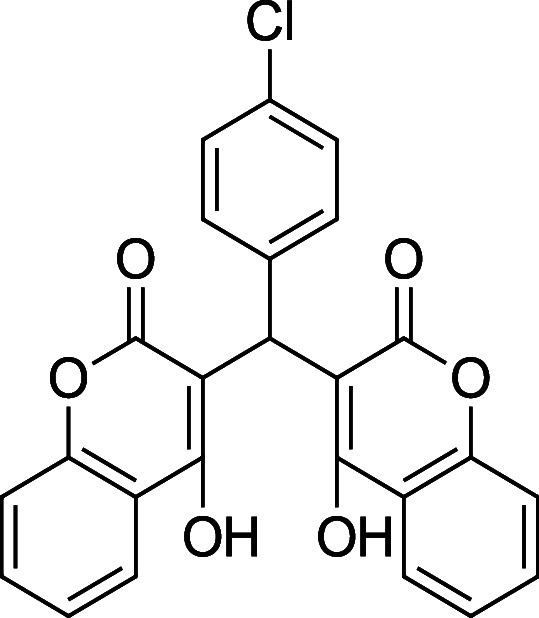


### **3c**. 3,3′-((4-Chlorophenyl)­methylene)­bis­(4-hydroxy-2H-chromen-2-one)

Average yield: 0.401 g (87–93%);^1^H NMR (300 MHz, CDCl_3_) δ 11.54 (s, 1H), 11.32 (s, 1H), 8.04 (dd, *J* = 7.8 Hz, 2H), 7.64 (td, *J* = 7.8, 1.5 Hz, 2H), 7.43–7.36 (m, 4H), 7.31–7.27 (m, 2H), 7.16 (d, *J* = 7.8 Hz, 2H), 6.04 (s,1H) ppm; ^1^H NMR (400 MHz, DMSO-*d*_6_) δ 11.49 (s, 2H), 7.91 (dd, *J* = 8.0, 1.2 Hz, 2H), 7.61–7.57 (m, 2H), 7.38–7.26 (m, 6H), 7.20–7.17 (m, 2H), 6.34 (s, 1H) ppm. Mp: 255–258 °C; ^13^C NMR (75 MHz, CDCl_3_) δ 169.2, 166.9, 166.1, 164.7, 152.6, 152.3, 133.9, 133.1, 132.7, 128.8, 128.0, 125.1, 124.5, 116.7, 105.3, 103.7, 35.9 ppm; ^13^C NMR (100 MHz, DMSO-*d*_6_) δ 165.4, 164.7, 152.3, 139.3, 132.0, 130.2, 128.7, 128.0, 124.0, 123.8, 117.9, 116.0, 104.0, 35.7 ppm; HRMS (ESI^–^) *m*/*z*: [M-1]^−^ calcd 445.0484, found 445.0464. IR (neat): 3064 (OH), 1663 (C=O), 1560 (C=C), 801 (C–Cl) cm^–1^.
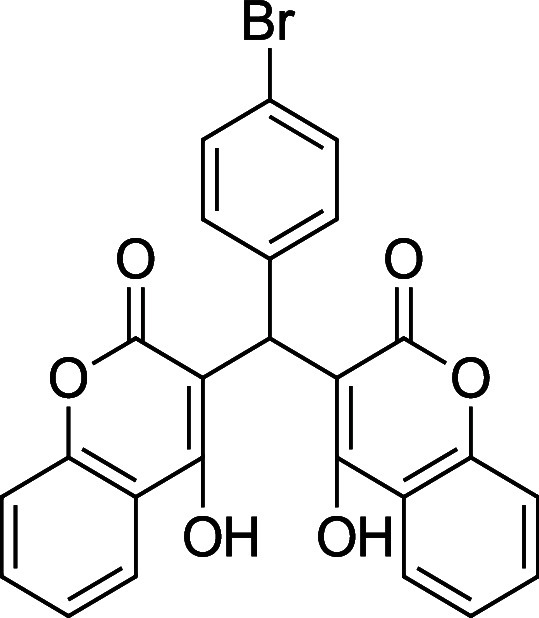


### **3d**. 3,3′-((4-Bromophenyl) methylene) bis­(4-hydroxy-2H-chromen-2-one)

Average yield: 0.403 g (79–85%); ^1^H NMR (300 MHz, CDCl_3_) δ 11.54 (s, 1H), 11.31 (s,1H), 8.03 (dd, *J* = 7.8 Hz, 2H), 7.64 (td, *J* = 7.8, 1.5 Hz, 2H), 7.46–7.36 (m, 6H), 7.10 (dd, *J* = 8.7, 1.2 Hz, 2H), 6.01 (s, 1H) ppm; Mp: 265–267 °C; ^13^C NMR (75 MHz, CDCl_3_) δ 169.3, 166.9, 166.1, 164.7, 152.6, 152.3, 134.5, 133.1, 131.8, 128.4, 125.1, 124.5, 120.9, 116.8, 116.4, 105.2, 103.7, 35.9 ppm; HRMS (ESI^–^) *m*/*z*: [M-1]^−^ calcd 488.9979, found 488.9998. IR (neat): 3064 (OH), 1659 (C=O), 1559 (C=C), 662 (C–Br) cm^–1^.
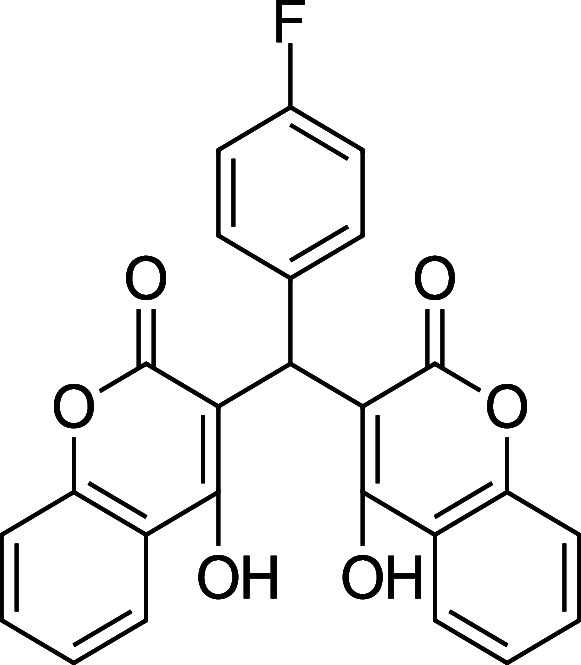


### **3e**. 3,3′-((4-Fluorophenyl) methylene) bis­(4-hydroxy-2H-chromen-2-one)

Average yield: 0.362 g (81–87%);^1^H NMR (300 MHz, CDCl_3_) δ 11.54 (s, 1H), 11.32 (s,1H), 8.03 (dd, *J* = 7.5 Hz, 2H), 7.63 (td, *J* = 7.8, 1.5 Hz, 2H), 7.41 (d, *J* = 8.1 Hz, 4H), 7.21–7.16 (m, 2H), 7.00 (t, *J* = 8.7 Hz, 2H), 6.05 (s, 1H) ppm; Mp: 206–208 °C; ^13^C NMR (75 MHz, CDCl_3_) δ 169.2, 166.9, 166.0, 164.6, 161.8 (d, ^1^ *J*_C–F_ = 244 Hz), 152.6, 152.3, 133.1, 130.9, 130.9, 128.3, 128.2, 125.0, 124.4, 116.9, 116.7, 116.4, 115.7, 115.4, 105.5, 104.0, 35.7 ppm; HRMS (ESI^–^) *m*/*z*: [M-1]^−^ calcd 429.0780, found 429.0773. 445.0484, found 445.0489. IR (neat): 3050 (OH), 1666 (C=O), 1558 (C=C), 1183 (C–F) cm^–1^.
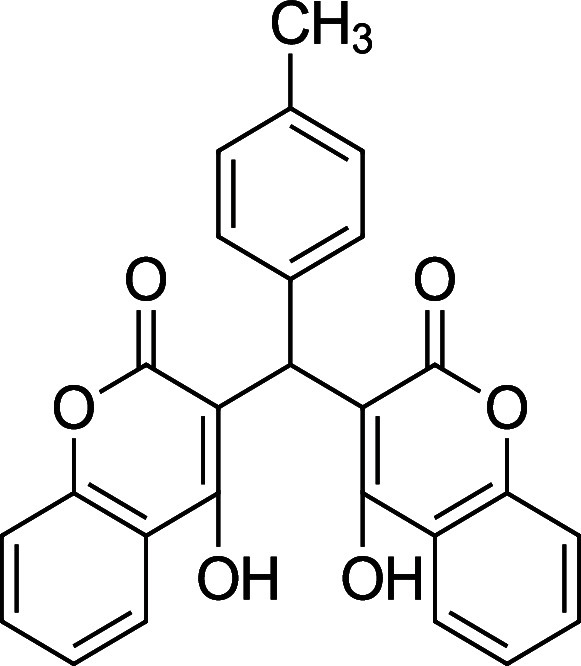


### **3f**. 3,3′-((*p*-Tolylmethylene) methylene) bis­(4-hydroxy-2H-chromen-2-one)

Average yield: 0.341 g (78–82%); ^1^H NMR (300 MHz, CDCl_3_) δ 11.51 (s, 1H), 11.29 (s, 1H), 8.08 (dd, *J* = 7.8 Hz, 2H), 7.62 (td, *J* = 7.8,1.5 Hz, 2H), 7.41 (d, *J* = 8.4 Hz, 4H), 7.12 (dd, *J* = 8.7, 2.1 Hz, 4H), 6.06 (d, *J* = 0.9 Hz, 1H), 2.34 (s, 3H) ppm; Mp: 256–260 °C; ^13^C NMR (75 MHz, CDCl_3_) δ 169.4, 167.0 165.8, 164.6, 152.6, 152.4, 136.6, 132.9, 132.1, 129.4, 126.4, 125.0, 124.5, 116.7, 105.8, 104.2, 35.9, 21.1 ppm; HRMS (ESI^–^) *m*/*z*: [M-1]^−^ calcd 425.1031, found 425.1047. IR (neat): 3016 (OH), 2608 (sp3 CH), 1661 (C=O), 1563 (C=C) cm^–1^.
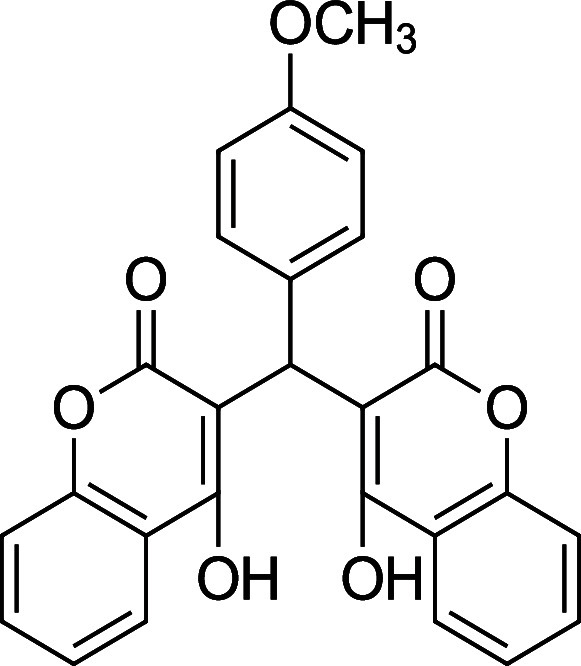


### **3g**. 3,3′-((4-Methoxyphenyl) methylene) bis­(4-hydroxy-2H-chromen-2-one)

Average yield: 0.393 g (85–93%); ^1^H NMR (300 MHz, CDCl_3_) δ 11.50 (s, 1H), 11.29 (s,1H), 8.05–8.01 (m, 2H), 7.62 (td, *J* = 7.8, 1.8 Hz, 2H), 7.42–7.39 (m, 4H), 7.12 (dd, *J* = 8.8, 1.2 Hz, 2H), 6.85 (dt, *J* = 8.7, 2.7 Hz, 2H), 6.05 (s, 1H), 3.80 (s, 3H) ppm; Mp: 240–243 °C; ^13^C NMR (75 MHz, CDCl_3_) δ 169.3, 166.9, 165.7, 164.6, 152.6, 152.3, 132.9, 127.7, 127.0, 124.9, 124.4, 117.0, 116.7, 116.5, 114.1, 105.8, 104.3, 55.3, 35.6 ppm; HRMS (ESI^–^) *m*/*z*: [M-1]^−^ calcd 441.0980, found 441.0993. IR (neat): 3064 (OH), 1660 (C=O), 1561 (C=C), 1257 (C–O) cm^–1^.
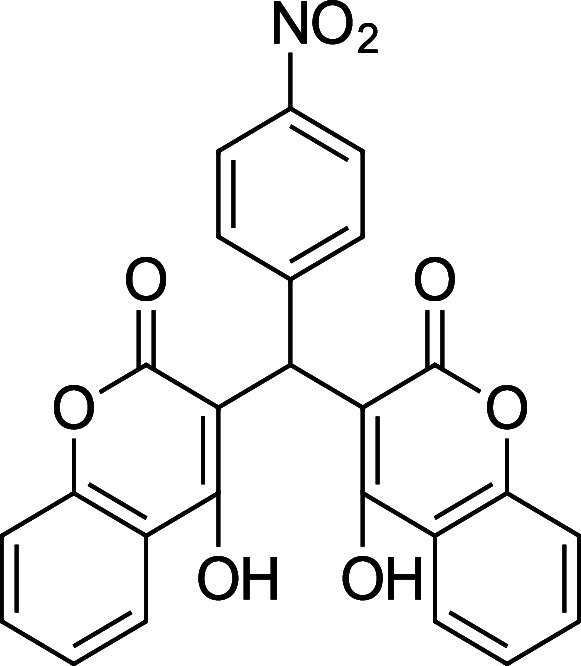


### **3h**. 3,3′-((4-Nitrophenyl)­methylene)­bis­(4-hydroxy-2H-chromen-2-one)

Average yield: 0.437 g (95–97%); ^1^H NMR (300 MHz, CDCl_3_) δ 11.57 (s, 1H), 11.38 (s, 1H), 8.22–8.17 (m, 2H), 8.10 (dd, *J* = 8.3, 1.7 Hz, 1H), 8.01 (dd, *J* = 8.4, 1.5 Hz, 1H), 7.67 (td, *J* = 8.1, 0.6 Hz, 2H), 7.46–7.39 (m, 6H), 6.12 (s, 1H) ppm; ^1^H NMR (400 MHz, DMSO-*d*_6_): δ 11.26 (bs, 2H, OH), 8.09 (dt, *J* = 8.8, 2.2 Hz, 2H, Ar–H), 7.89 (dd, *J* = 7.8, 1.4 Hz, 4H, Ar–H), 7.60–7.56 (m, 2H, Ar–H), 7.49–7.42 (m, 2H, Ar–H), 7.35 (dd, *J* = 8.4, 0.8 Hz, 2H Ar–H), 7.32–7.28 (m, 2H, Ar–H), 6.41 (s, 1H, CH) ppm; Mp: 244–246 °C; ^13^C NMR (75 MHz, CDCl_3_) δ 169.1, 167.0, 152.6, 152.4, 146.9, 143.4, 133.4, 127.6, 125.3, 125.2, 124.5, 123.9, 116.8, 116.8, 116.7, 116.3, 104.8, 103.3, 36.6 ppm; ^13^C NMR (100 MHz, DMSO-*d*_6_): δ 166.7, 164.9, 152.9, 150.4, 146.0, 132.3, 128.6, 124.5, 124.0, 123.7, 118.9, 116.4, 103.8, 37.2 ppm; HRMS (ESI^–^) *m*/*z*: [M-1]^−^ calcd 456.0725, found 456.0730. IR (neat): 3064 (OH), 1661 (C=O), 1561 (C=C), 1349 (NO_2_) cm^–1^.
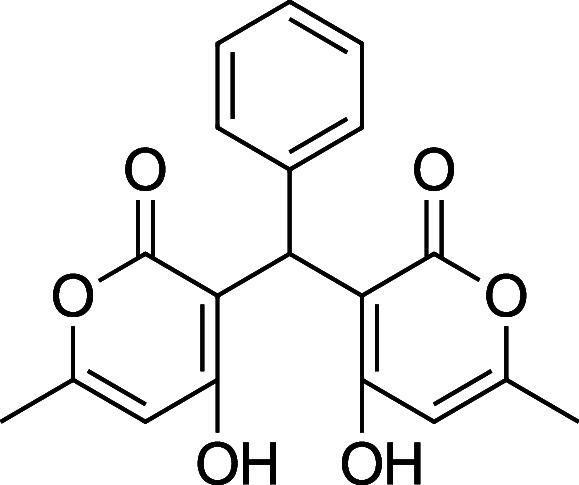


### **4a**. 3,3′-(Phenylmethylene)­bis­(4-hydroxy-6-methyl-2H-pyran-2-one)

Average yield: 0.297 g (84–90%); ^1^H NMR (300 MHz, CDCl_3_) δ 10.93 (s, 1H), 10.73 (s, 1H), 7.34–7.28 (m, 2H), 7.22–7.21 (m, 1H), 7.18–7.14 (m, 2H), 6.08**–**6.06 (m, 2H), 5.75 (s, 1H), 2.30 (s, 3H), 2.29 (s, 3H) ppm; Mp: 209–211 °C; ^13^C NMR (75 MHz, CDCl_3_) δ 169.9, 169.1, 161.7, 135.5, 128.5, 126.7, 126.5, 103.8, 103.2, 34.8, 19.7 ppm; HRMS (ESI^+^) *m*/*z*: [M + Na]^+^ calcd 363.0839, found 363.0835. IR (neat): 3138 (OH), 1667 (C=O), 1562 (C=C) cm^–1^.
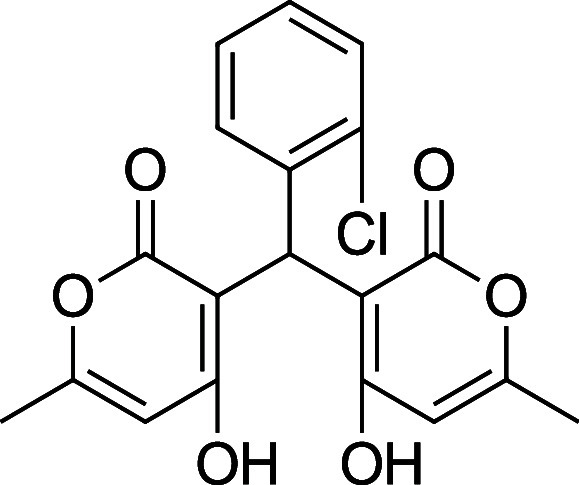


### **4b**. 3,3′-(2-Chlorophenyl) methylene) bis­(4-hydroxy-6-methyl-2H-pyran-2-one)

Average yield: 0.299 g (76–84%); ^1^H NMR (300 MHz, CDCl_3_) δ 11.15 (s, 2H), 7.40–7.31 (m, 2H), 7.26–7.16 (m, 2H), 6.09 (s, 2H), 5.91 (s, 1H), 2.27 (s, 6H) ppm; Mp: 155–158 °C; ^13^C NMR (75 MHz, CDCl_3_) δ 169.6, 169.1, 161.7, 134.6, 133.5, 130.6, 129.3, 128.5, 126.8, 103.4, 34.2,19.8 ppm; HRMS (ESI^+^) *m*/*z*: [M + Na]^+^ calcd 397.0449, found 397.0449. IR (neat): 3057 (OH), 1669 (C=O), 1573 (C=C), 701 (C–Cl) cm^–1^.
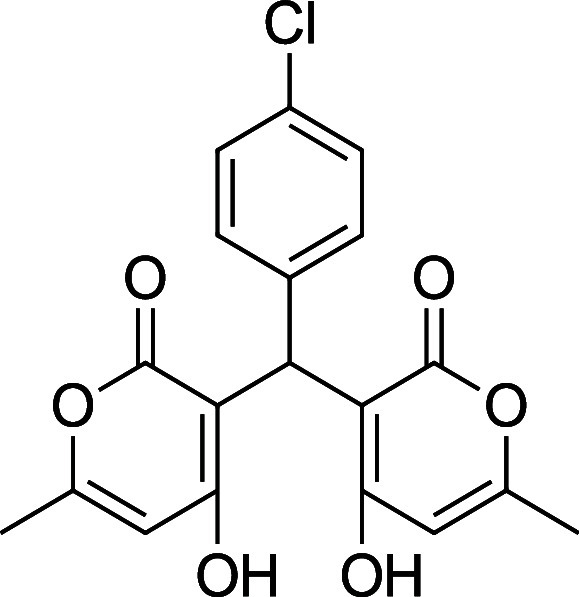


### **4c**. 3,3′-(4-Chlorophenyl) methylene) bis­(4-hydroxy-6-methyl-2H-pyran-2-one)

Average yield: 0.307 g (80–84%); ^1^H NMR (300 MHz, CDCl_3_) δ 10.93 (s, 1H), 10.72 (s, 1H), 7.30–7.26 (m, 2H), 7.08 (dd, *J* = 7.8, 2.1 Hz, 2H), 6.10 (s, 1H), 6.03 (s, 1H), 5.69 (s, 1H), 2.30 (s, 3H), 2.29 (s,3H) ppm; Mp: 202–204 °C; ^13^C NMR (75 MHz, CDCl_3_) δ 170.2, 169.1, 161.8, 134.2, 132.5, 128.7, 128.0, 103.4, 103.1, 34.4,19.8 ppm; HRMS (ESI^+^) *m*/*z*: [M + Na]^+^ calcd 397.0449, found 397.0435. IR (neat): 3095 (OH), 1675 (C=O), 1567 (C=C), 691 (C–Cl) cm^–1^.
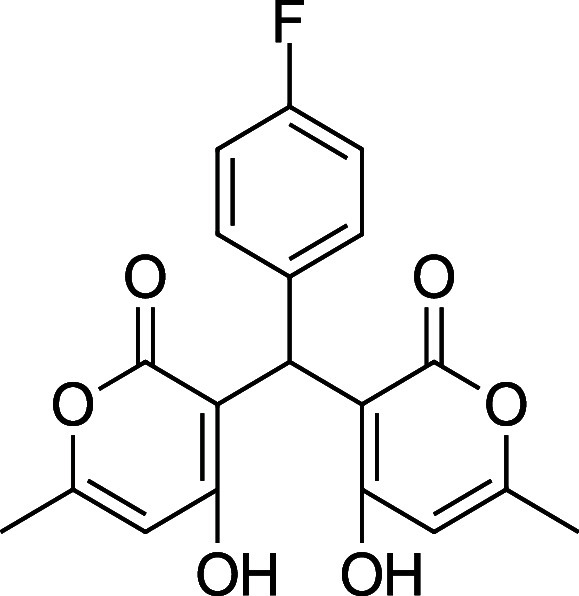


### **4e**. 3,3′-(4-Fluorophenyl) methylene) bis­(4-hydroxy-6-methyl-2H-pyran-2-one)

Average yield: 0.319 g (87–91%); ^1^H NMR (300 MHz, CDCl_3_) δ 10.94 (s, 2H), 7.14–7.09 (m, 2H), 7.02–6.96 (m, 2H), 6.07 (s, 1H), 6.05 (s, 1H), 5.71 (s, 1H), 2.29 (s, 3H), 2.29 (s, 3H) ppm; Mp: 215–218 °C; ^13^C NMR (75 MHz, CDCl_3_) δ 170.1, 161.9, 161.1 (d, ^1^ *J*_C–F_= 243 Hz), 160.0, 131.2, 131.2, 128.2, 128.1, 115.5, 115.2, 103.3, 34.3,19.8 ppm; HRMS (ESI^+^) *m*/*z*: [M + Na]^+^ calcd 381.0745, found 381.0751. IR (neat): 3094 (OH), 1675 (C=O), 1564 (C=C), 1157 (C–F) cm^–1^.
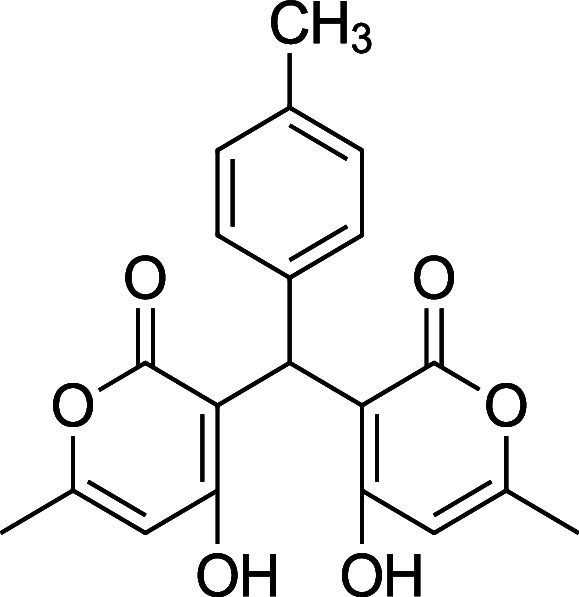


### **4f**. 3,3′-(p-Tolylmethylene) bis­(4-hydroxy-6-methyl-2H-pyran-2-one)

Average yield: 0.283 g (74–86%); ^1^H NMR (300 MHz, CDCl_3_) δ 10.88 (s, 2H), 7.11 (d, *J* = 8.0 Hz, 2H), 7.02 (d, *J* = 7.4 Hz, 2H), 6.05 (s, 2H), 5.72 (s, 1H), 2.32 (s, 3H), 2.29 (s, 3H) ppm; Mp: 178–181 °C; ^13^C NMR (75 MHz, CDCl_3_) δ 170.0, 169.9, 161.7, 136.3, 132.4, 129.3, 126.4, 103.1, 34.5, 21.0, 19.7 ppm; HRMS (ESI^+^) *m*/*z*: [M + Na]^+^ calcd 377.0996, found 377.0988. IR (neat): 3093 (OH), 1675 (C=O), 1561 (C=C) cm^–1^.
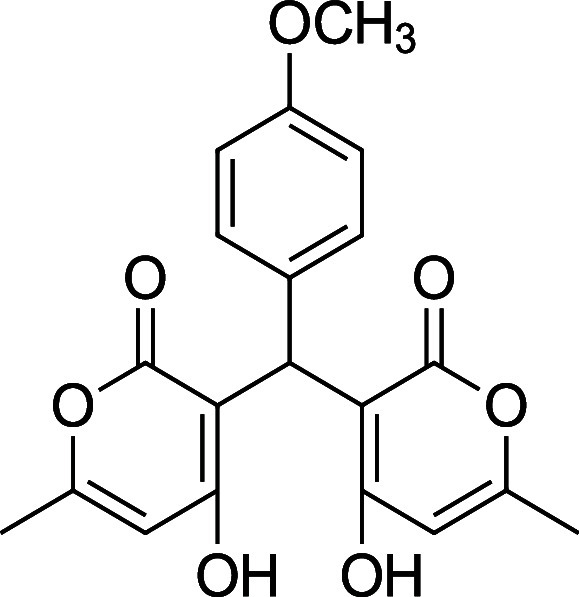


### **4g**. 3,3′-(4-Methoxy) methylene) bis­(4-hydroxy-6-methyl-2H-pyran-2-one)

Average yield: 0.301 g (79–83%); ^1^H NMR (300 MHz, CDCl_3_) δ 10.92 (s, 2H), 7.06 (dd, *J* = 9.0, 1.2 Hz, 2H), 6.83 (dd, *J* = 6.9, 2.2 Hz, 2H), 6.05 (s, 2H), 5.70 (s, 1H), 3.79 (s, 3H), 2.28 (s, 3H), 2.28 (s, 3H) ppm; Mp: 167–169 °C; ^13^C NMR (75 MHz, CDCl_3_) δ 169.5, 161.6, 158.3, 127.6, 127.3, 113.9, 103.2, 55.3, 34.1, 19.7 ppm; HRMS (ESI^+^) *m*/*z*: [M + Na]^+^ calcd 393.0945, found 393.0945. IR (neat): 3131 (OH), 1678 (C=O), 1561 (C=C), 1245 (C–O) cm^–1^.
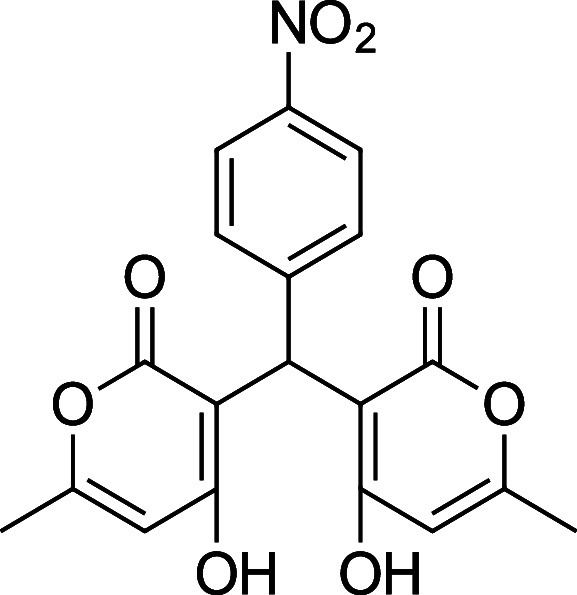


### **4h**. 3,3′-(4-Nitrophenyl) methylene) bis­(4-hydroxy-6-methyl-2H-pyran-2-one)

Average yield: 0.305 g (72–86%); ^1^H NMR (300 MHz, CDCl_3_) δ 10.98 (s, 2H), 8.19–8.14 (m, 2H), 7.33 (dd, *J* = 9.1, 1.2 Hz, 1H), 6.15 (s, 1H), 6.06 (s, 1H), 5.78 (s, 1H), 2.32 (s, 3H), 2.31 (s, 3H) ppm; ^1^H NMR (400 MHz, DMSO-*d*_6_) δ 8.09 (d, *J* = 8.8 Hz, 2H), 7.29 (dd, *J* = 9.2, 0.8 Hz, 2H), 6.05 (s, 1H), 6.05 (s, 1H), 5.84 (s, 1H), 2.19 (s, 6H) ppm. Mp: 221–224 °C; ^13^C NMR (75 MHz, CDCl_3_) δ 170.2, 162.2, 146.8, 143.8, 127.6, 123.8, 103.3, 35.2, 19.9 ppm; ^13^C NMR (100 MHz, DMSO-*d*_6_) δ 167.6, 165.2, 161.3, 149.6, 145.6, 128.3, 123.1, 100.9, 100.7, 35.4, 19.2 ppm; HRMS (ESI^+^) *m*/*z*: [M + Na]^+^ calcd 408.0690, found 408.0684. IR (neat): 3057 (OH), 1672 (C=O), 1561 (NO_2_), 1346 (NO_2_) cm^–1^.
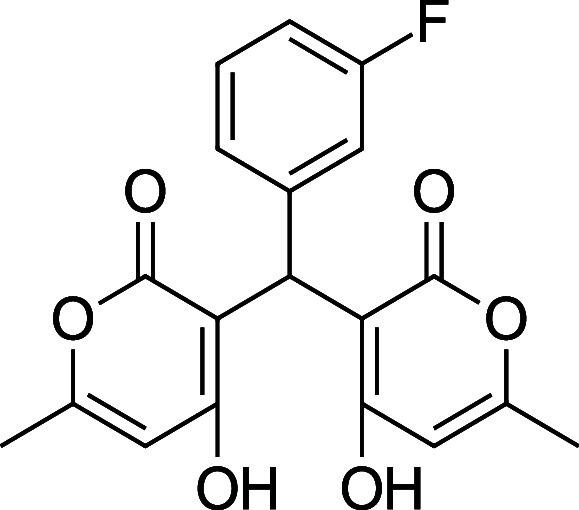


### **4i**. 3,3′-(3-Fluorophenyl) methylene) bis­(4-hydroxy-6-methyl-2H-pyran-2-one)

Yield: 0.146 g (24%) ^1^H NMR (300 MHz, CDCl_3_) δ 11.08 (s, 2H), 7.26 (td, *J* = 8.0, 6.2 Hz, 1H), 6.95–6.91 (m, 2H), 6.91 (d, *J* = 2.5 Hz, 1H), 6.88–6.85 (m, 1H), 6.09 (s, 2H), 5.77 (s, 1H), 2.29 (s, 3H); ^1^H NMR (400 MHz, DMSO-*d*_6_) δ 11.72 (s, 2H), 7.30–7.24 (m, 1H), 7.00–6.95 (m, 1H), 6.86 (d, *J* = 8.0 Hz, 1H), 6.78 (d, *J* = 8.0 Hz, 1H), 6.08 (s, 2H), 5.90 (s, 1H), 2.20 (s, 3H) ppm. Mp: 217.6–220.3 °C d (yellow) ^13^C NMR (100 MHz, CDCl_3_, 50 °C) δ 169.6, 163.3 (d,^1^ *J*_C–F_ = 244 Hz) 161.9, 138.7 (d,^2^ *J*_C–F_ = 23 Hz), 129.9 (d,^3^ *J*_C–F_ = 8 Hz), 122.2 (d,^4^ *J*_C–F_ = 3 Hz), 113.9 (d,^2^ *J*_C–F_ = 20 Hz), 113.7, 113.6, 103.2, 34.8, 19.6 ppm; HRMS (ESI^+^) *m*/*z*: [M + Na]^+^ calcd 381.07451, found 381.0742. IR (neat): 3057 (OH), 1664 (C=O), 1213 (C–F) cm^–1^.

## Supplementary Material





## Notes

^1^H NMR, ^13^C NMR, FTIR, HRMS spectra for all compounds; various spectroscopic results for compounds C-1 through C-3 and pyrone dimers; the table of computational values for **4h**; relevant spectra for atropoisomerism for **3c, 3h, 4h, 4i** (PDF)
The X-ray crystal structure of **4h** (CIF)

## ⊥

Currently a graduate student at Worcester Polytechnic Institute, Department of Chemistry and Biochemistry, 100 Institute Rd, Worcester, MA 01609, USA

## #

Currently a graduate student at Colorado State University, Department of Chemistry, 1301 Center Ave Mall, Chemistry B101, Ft. Collins, CO 80523-1872, USA.
