# Supplementary material for: Multicomponent Green Synthesis Involving Aryl Aldehydes and Trapped Enols: Dimerization over Cyclization
Source: ACS Omega. 2026 Jan 22;11(4):5112–21. doi: 10.1021/acsomega.5c07008 (PMC12878747; doi:10.1021/acsomega.5c07008)
Supplement: Supplementary file 1 [file ao5c07008_si_002.pdf]

Multicomponent Green Synthesis Involving Aryl Aldehydes and Trapped Enols: Dimerization over Cyclization

Sarah K. Zingales,[ab] McKenna Gibson,[b1] Julio Tapia-Hernandez,[c] Kendall Jenkins[c2], Mitchell Munzing,[c] Grace Dickerson,[c] Selena Speikers,[c] David J. Frazer,[a] Clifford W. Padgett,[d] Michael T. Wentzel[c]*

[a]United States Coast Guard Academy, Department of Chemical and Environmental Sciences, 31 Mohegan Ave Pkwy, New London, CT 06320, USA

[b]University of Saint Joseph, Department of Chemistry, 1678 Asylum Ave, West Hartford, CT 06117, USA

[c]Augsburg University, Department of Chemistry, 2211 Riverside Ave, Minneapolis, MN 55454

[d]Georgia Southern University, Department of Chemistry and Biochemistry, 11935 Abercorn St, Savannah, GA 31419, USA

[1] currently a graduate student at Worcester Polytechnic Institute Department of Chemistry and Biochemistry 100 Institute Rd, Worcester, MA 01609, USA
[2] currently a graduate student at Colorado State University Department of Chemistry 1301 Center Ave Mall, Chemistry B101, Ft. Collins, CO 80523-1872, USAh

SI Table of Contents
Page

Table S-1. Acetylcholinesterase inhibition activity 6
Figure S-1. Computational results of mechanistic pathways. 7
Figure S-2. 1H NMR of reaction mixture including **P-8** aldol (red asterisk) and E-**P-8**. 8
Figure S-3. ^{1}H NMR of representative open and closed pyrone dimers **P-8** and E-**P-8**. ..9
Table S-2. Bis-pyrone dimers characterization 9
Table S-3. Calculated energy and distance between the OH and C=O of the two pyrone rings with respect to rotation of one pyrone ring for **P-1**. 12
FigureS-4. ^{1}H NMR spectrum of **C-1** in $CDCl_3$. 13
FigureS-5. ^{13}C NMR spectrum of **C-1** in $CDCl_3$. 14
FigureS-6. HRMS results of **C-1**. 15
FigureS-7. HRMS results of **C-1** spectrum. 16
FigureS-8. HRMS results of **C-1** peak list. 17
FigureS-9. IR spectrum of **C-1**. 18
FigureS-10. ^{1}H NMR spectrum of **C-2** in $CDCl_3$. 19
FigureS-11. ^{13}C NMR spectrum of **C-1** in $CDCl_3$. 20
FigureS-12. HRMS results of **C-2** 21
FigureS-13. HRMS results of **C-2** spectrum. 22
FigureS-14. HRMS results of **C-2** peak list. 23
FigureS-15. IR spectrum of **C-2**. 24
FigureS-16. ^{1}H NMR spectrum of **C-3** in CDCl3. 25
FigureS-17. 13C NMR spectrum of **C-3** in CDCl3. 26
FigureS-18. DMSO-d_6 ^{1}H NMR, ^{13}C NMR spectra of **C-3**. 27
FigureS-19. DMSO-d_6 ^{1}H NMR, ^{13}C NMR spectra of **C-3**. 28
FigureS-20. HRMS results of **C-3** 29
FigureS-21. HRMS results of **C-3** spectrum. 30
FigureS-22. HRMS results of **C-3** peak list. 31
FigureS-23. Figure S-8. IR spectrum of **C-3**. 32
FigureS-24. ^{1}H NMR spectrum of **C-4** in $CDCl_3$ 33
FigureS-25. ^{13}C NMR spectrum of **C-4** in $CDCl_3$. 34
FigureS-26. HRMS results of **C-4** 35

FigureS-27. HRMS results of **C-4** spectrum. 36
FigureS-28. HRMS results of **C-4** peak list. 37
FigureS-29. IR spectrum of **C-4**. 38
FigureS-30. ^{1}H NMR spectrum of **C-5** in $CDCl_3$. 39
FigureS-31. ^{13}C NMR spectrum of **C-5** in $CDCl_3$. 40
FigureS-32. HRMS results of **C-5** 41
FigureS-33. HRMS results of **C-5** spectrum. 42
FigureS-34. HRMS results of **C-5** peak list. 43
FigureS-35. IR spectrum of **C-5**. 44
FigureS-36. ^{1}H NMR spectrum of **C-6** in $CDCl_3$. 45
FigureS-37. ^{13}C NMR spectrum of **C-6** in $CDCl_3$. 46
FigureS-38. HRMS results of **C-6** 47
FigureS-39. HRMS results of **C-6** spectrum. 48
FigureS-40. HRMS results of **C-6** peak list. 49
FigureS-41. IR spectrum of **C-6**. 50
FigureS-42. ^{1}H NMR spectrum of **C-7** in $CDCl_3$. 51
FigureS-43. ^{13}C NMR spectrum of **C-7** in $CDCl_3$. 52
FigureS-45. HRMS results of **C-7** spectrum. 54
FigureS-46. HRMS results of **C-7** peak list. 55
FigureS-47. IR spectrum of **C-7**. 56
FigureS-48. ^{1}H NMR spectrum of **C-8** in $CDCl_3$. 57
FigureS-49. ^{13}C NMR spectrum of **C-8** in $CDCl_3$. 58
FigureS-50. DMSO-d_6 1H NMR 59
FigureS-51. DMSO-d_6 ^{13}C NMR spectra of **C-8** 60
FigureS-52. HRMS results of **C-8** 61
FigureS-53. HRMS results of **C-8** spectrum. 62
FigureS-54. HRMS results of **C-8** peak list. 63
FigureS-55. IR spectrum of **C-8**. 64
FigureS-56. ^{1}H NMR spectrum of **P-1** in $CDCl_3$. 65
FigureS-57. ^{13}C NMR spectrum of **P-1** in $CDCl_3$ 66
FigureS-59. HRMS results of **P-1** spectrum 68

FigureS-60. HRMS results of **P-1** peak list. 69
FigureS-61. IR spectrum of **P-1**. 70
FigureS-62. ^{1}H NMR spectrum of **P-2** in $CDCl_3$. 71
FigureS-63. ^{13}C NMR spectrum of **P-2** in $CDCl_3$....72
FigureS-64. HRMS results of **P-2** 73
FigureS-65. HRMS results of **P-2** spectrum....74
FigureS-66. HRMS results of **P-2** peak list. 75
FigureS-67. IR spectrum of **P-2**. 76
FigureS-68. ^{1}H NMR spectrum of **P-3** in $CDCl_3$. 77
FigureS-69. ^{13}C NMR spectrum of **P-3** in $CDCl_3$....78
FigureS-70. HRMS results of **P-3** 79
FigureS-71. HRMS results of **P-3** spectrum....80
FigureS-72. HRMS results of **P-3** peak list. 81
FigureS-73. IR spectrum of **P-3** 82
FigureS-74. ^{1}H NMR spectrum of **P-4** in $CDCl_3$. 83
FigureS-75. ^{13}C NMR spectrum of **P-4** in $CDCl_3$....84
FigureS-76. HRMS results of **P-4** 85
FigureS-77. HRMS results of **P-4** spectrum....86
FigureS-78. HRMS results of **C-1** peak list. 87
FigureS-79. IR spectrum of **P-3**. 88
FigureS-80. ^{1}H NMR spectrum of **P-5** in $CDCl_3$. 89
FigureS-81. ^{13}C NMR spectrum of **P-5** in $CDCl_3$....90
FigureS-82. HRMS results of **P-5** 91
FigureS-83. HRMS results of **P-5** spectrum....92
FigureS-84. HRMS results of **P-5** peak list. 93
FigureS-85. IR spectrum of **P-5**. 94
FigureS-86. ^{1}H NMR spectrum of **P-6** in $CDCl_3$. 95
FigureS-87. ^{13}C NMR spectrum of **P-6** in $CDCl_3$....96
FigureS-88. HRMS results of **P-6** 97
FigureS-89. HRMS results of **P-6** spectrum....98
FigureS-90. HRMS results of **P-6** peak list. 99

FigureS-91. IR spectrum of **P-6**. 100
FigureS-92. ^{1}H NMR spectrum of **P-7** in $CDCl_3$. 101
FigureS-93. ^{13}C NMR spectrum of **P-7** in $CDCl_3$ 102
FigureS-94. DMSO-d_6 1H NMR spectra of **P-7** 103
FigureS-95. DMSO-d_6 13C NMR spectra of **P-7** 104
FigureS-96. HRMS results of **P-7** 105
FigureS-97. HRMS results of **P-7** spectrum 106
FigureS-98. HRMS results of **P-7** peak list. 107
FigureS-99. IR spectrum of **P-7**. 108
FigureS-100. ^{1}H NMR spectrum of **P-8** in $CDCl_3$. 109
FigureS-101. DMSO-d_6 ^{1}H NMR spectra of **P-9** 110
FigureS-102. DMSO-d_6 ^{13}C NMR spectra of **P-9** 111
FigureS-103. HRMS results of **P-9** 112
FigureS-104. HRMS results of **P-9** spectrum.

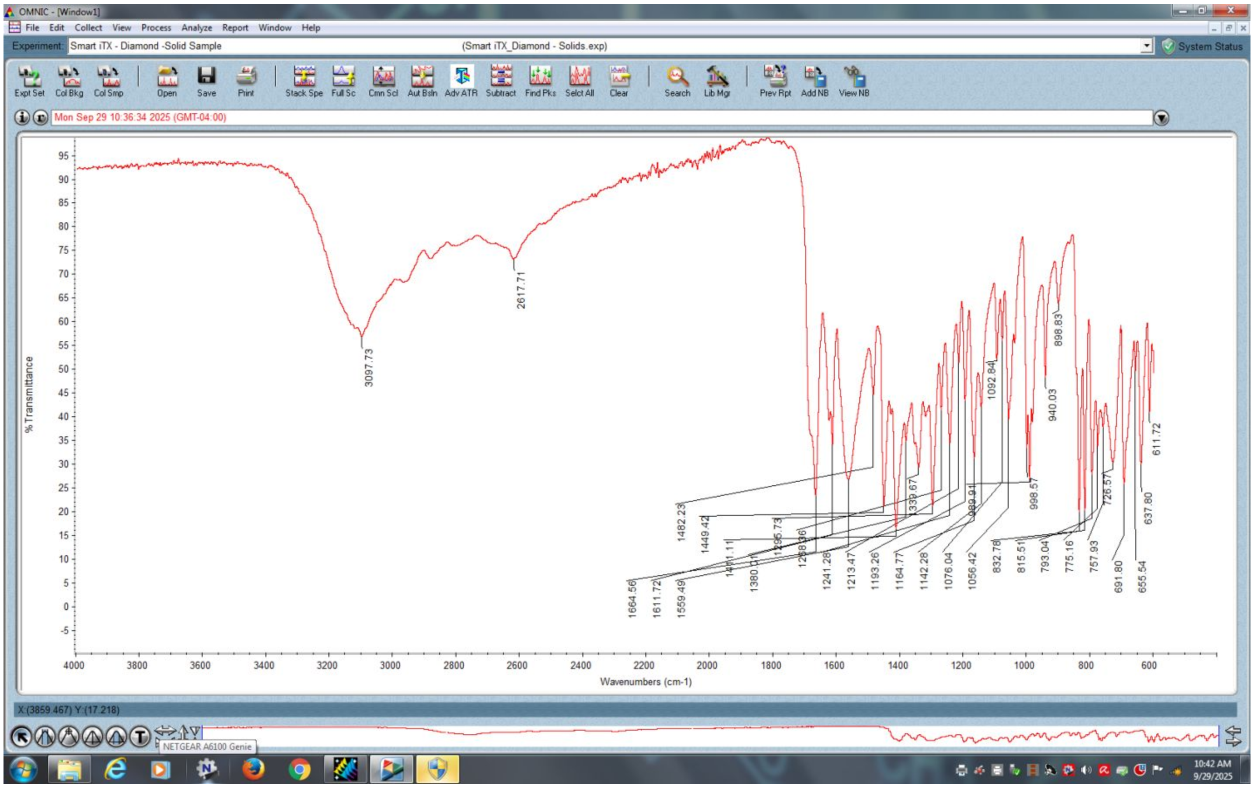

....................112
....................113
FigureS-105. IR spectrum of **P-7** 113
References: 113

Table S-1. Acetylcholinesterase inhibition activity. Method followed from Sigma Aldrich MAK 324 Acetylcholinesterase Inhibitor Screening Kit. Briefly, for the no-enzyme control, assay buffer (45 μL) and ultrapure water (5 μL) were added, and after 15 minutes reaction mix (150 μL) was added. For the no-inhibitor control, prepped AChE solution (45 μL) and ultrapure water (5 μL) were added, and after 15 minutes reaction mix (150 μL) was added. For the positive control (known inhibitor phystostigmine), prepped AChE solution (45 μL) and test solution (5 μL of 10^{-2} M) were added, and after 15 minutes reaction mix (150 μL) was added. For the test wells, prepped AChE solution (45 μL) and test solution (5 μL of 10^{-2} M) were added, and after 15 minutes reaction mix (150 μL) was added. Absorbance was read at 412 nm and control 2 was set to 100% activity/0% inhibition. Activity/inhibition was reported as averages of number of runs.

	% inhibition
Control 1 (no enzyme, n = 2)	100%
Control 2 (no inhibitor, n = 2))	0%
Phytostigmine (n = 3)	100%
**P-3** (n = 2)	0%
**C-3** (n = 4)	6%

**P-5** (n =2)	4%
**C-6** (n =2)	12%

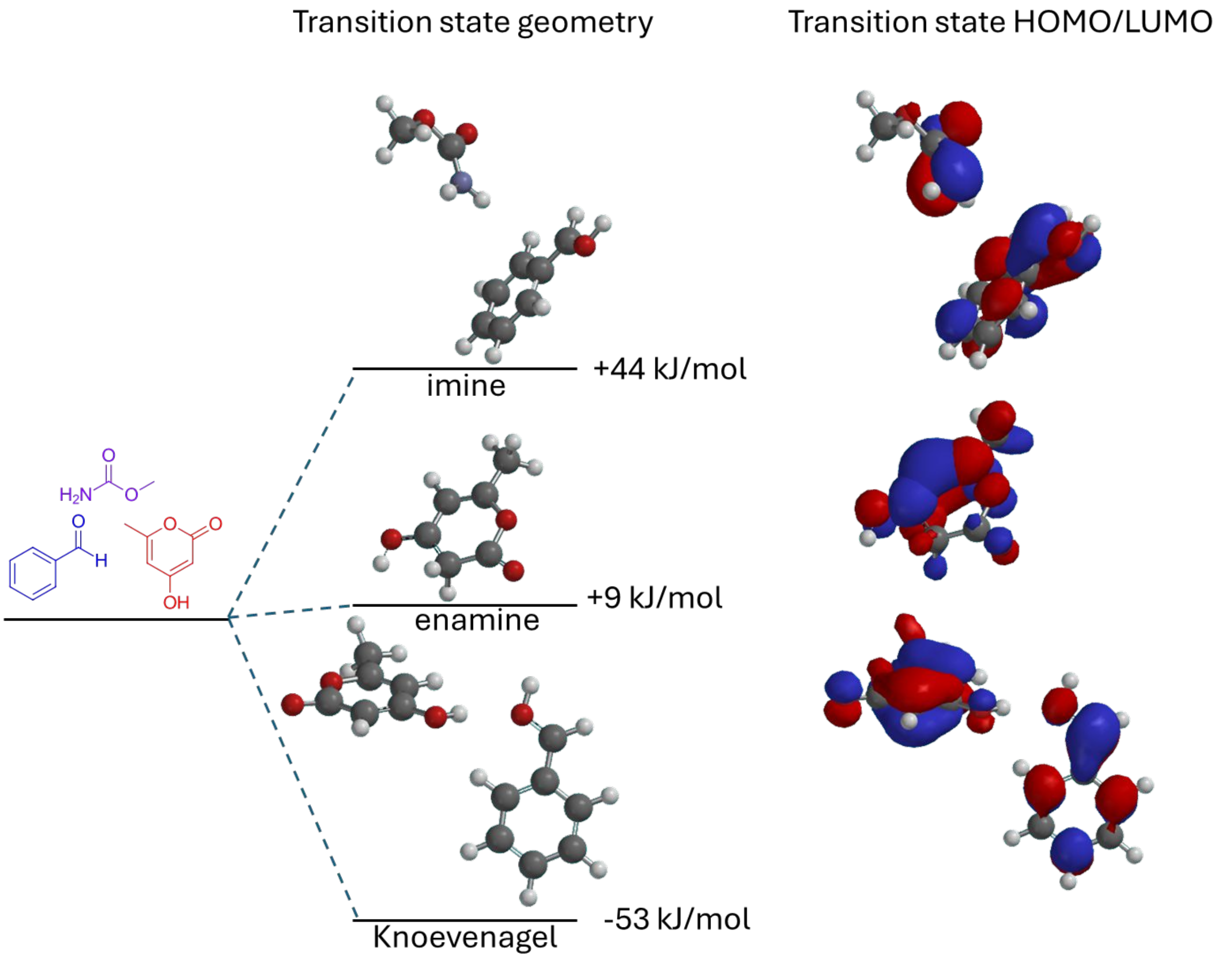

Figure S-1. Computational results of mechanistic pathways. Spartan student v9 was used to calculate transition state geometries, heat of formation, and HOMO/LUMO orbitals.

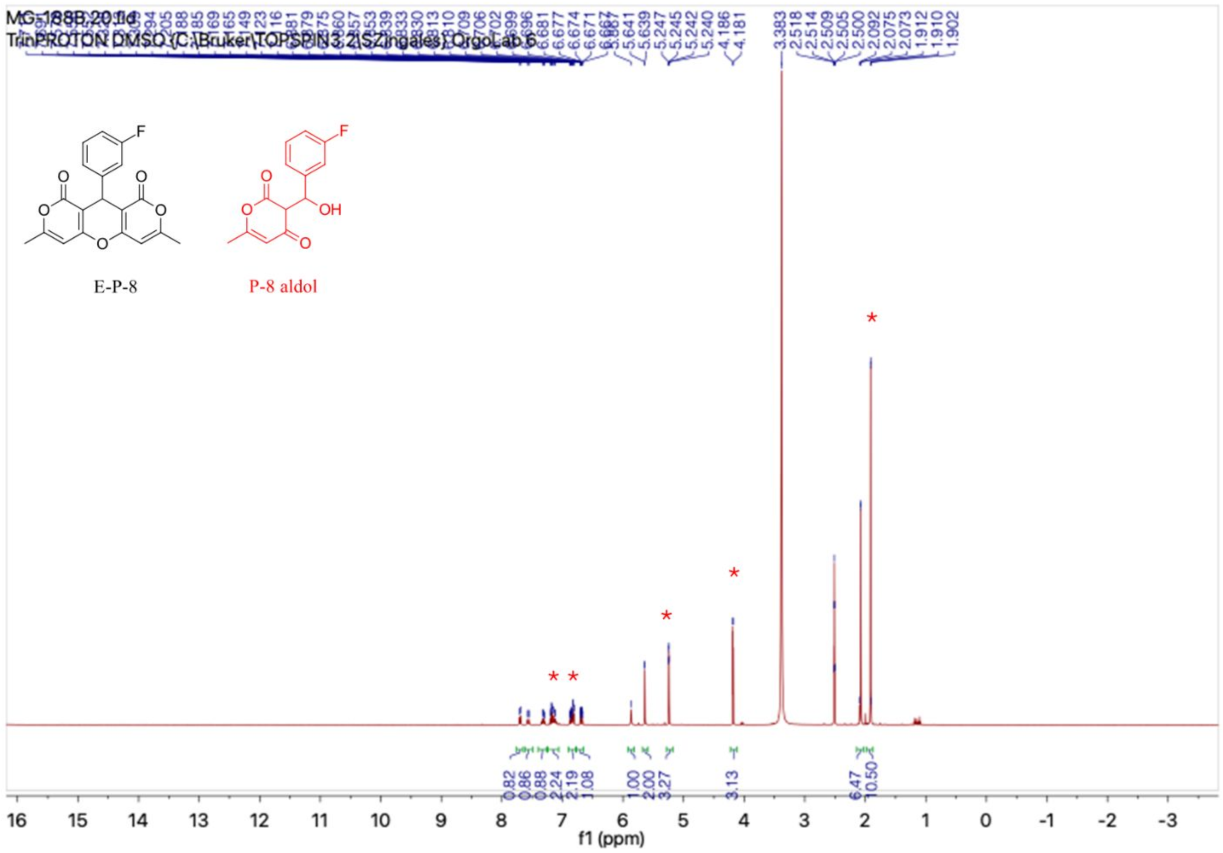

Figure S-2. 1H NMR of reaction mixture including P-8 aldol (red asterisk) and E-P-8.

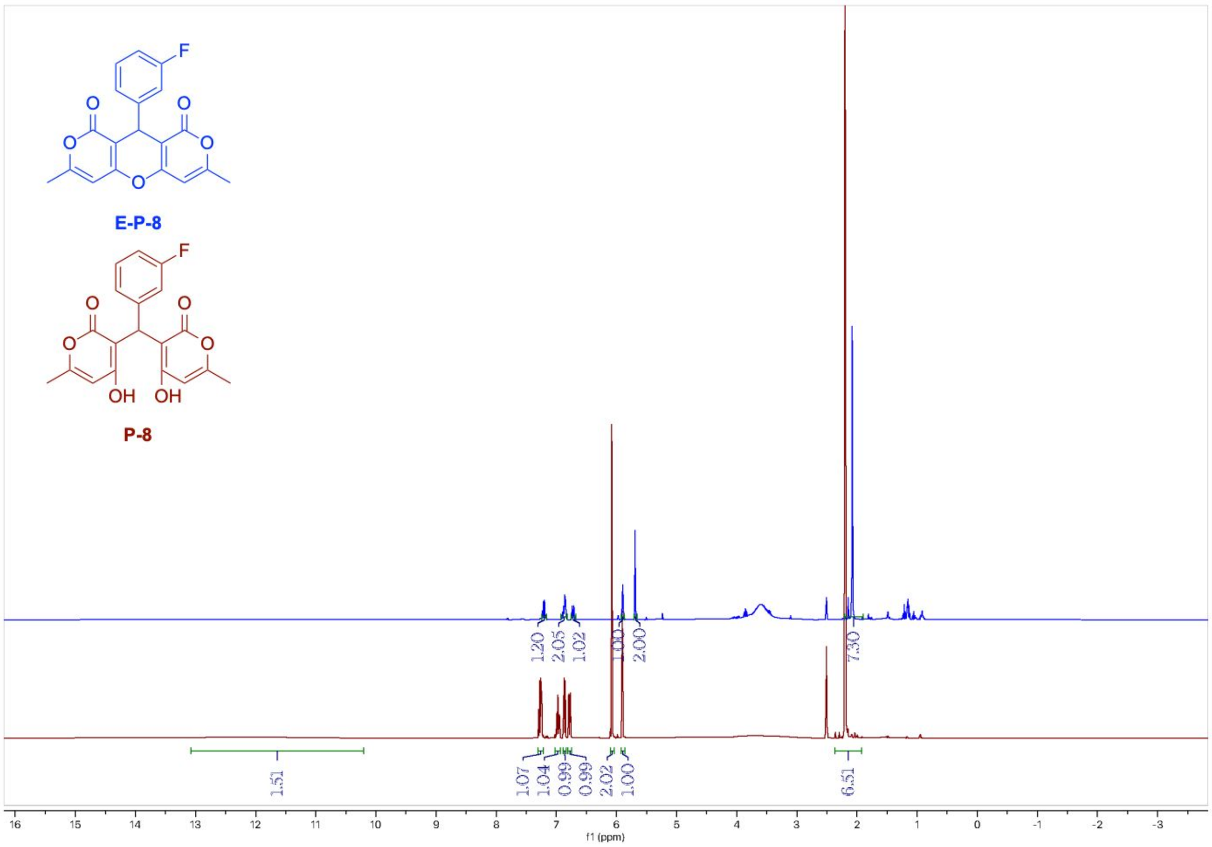

Figure S-3. ^{1}H NMR of representative open and closed pyrone dimers P-8 and E-P-8. P-8 was heated for 3 hours in toluene to convert to **E-P-8**. Stacked ^{1}H NMR of open and closed ring pyrone dimers **P-8** 3,3'-(3-fluorophenyl) methylene) bis(4-hydroxy-6-methyl-2H-pyran-2-one) and **E-P-8** 7-(3-fluorophenyl)-7,14b-dihydro-4a*H*-pyrano[3,2-*c*;5,6-*c′*]dichromene-6,8-dione. Red (bottom) is the typical open dimer as evidenced by the two OH peaks ~11 ppm and the 2:1 ratio of alkene:methine CH. Blue (top) is the closed/epoxy dimer as evidenced by lack of OHs and the 1:2 ratio of methine:alkene CH which has been shifted slightly downfield.

Table S-2. Bis-pyrone dimers characterization

Product	R	Comparison to reference methods	New characterization data
**P-1**	Ph	[1]stoichiometric acid catalyst, 16 h, silica gel purification, 55% yield (^{1}H NMR missing OH peaks, no SI ^{13}C spectrum, incorrect mp)<br>[2]excess base, heavy metal catalyst, excess starting materials, toluene solvent, silica gel purification, 51% yield (^{1}H NMR impure, missing OH – 1:2 ratio)<br>[3]reaction in organic solvent, chlorinated solvent extraction (no SI spectra)<br>[4]ionic liquid, alcohol for purification (no SI spectra)<br>[5]stoichiometric acid catalyst and organic reagent, organic solvent, chlorinated solvent reaction (no SI spectra)<br>[6]excess acid, 8 hour heating, alcohol for purification 54% yield (no SI spectra)<br>[7]excess urea, metal catalyst, alcohol for purification 65% yield (no SI spectra)	^{1}H and ^{13}C NMR at full scale, FTIR, HRMS
**P-2**	2-Cl-Ph	[8]excess SDS, 6 hours, DMF for purification (no SI spectra)<br>[9]complex catalysts, alcohol for purification	HRMS
**P-3**	4-Cl-Ph	[2]excess base, heavy metal catalyst, excess starting materials, toluene solvent, silica gel purification, 70% yield (^{1}H NMR impure, missing OH)<br>[3]ionic liquid, alcohol for purification (no SI spectra)<br>[4]stoichiometric acid catalyst and organic reagent, organic solvent, chlorinated solvent reaction, (only ^{1}H, no SI spectra)<br>[5]excess acid, 8 hour heating, alcohol for purification, 71% yield (no SI spectra)<br>[22] [6]excess SDS, 10 hours, DMF for purification (no SI spectra)	^{1}H and ^{13}C NMR at full scale, FTIR, HRMS
**P-4**	4-F-Ph	[4]ionic liquid, alcohol for purification (no SI spectra)	^{1}H and ^{13}C NMR, FTIR, HRMS

**P-5**	4-CH_3-Ph	[4]ionic liquid, alcohol for purification (no SI spectra)<br>[8]excess SDS, 12 hours, DMF for purification (no SI spectra)	^{1}H and ^{13}C NMR, FTIR, HRMS
**P-6**	4-CH_3O-Ph	[2]excess base, heavy metal catalyst, excess starting materials, toluene solvent, silica gel purification, 60% yield (^{1}H NMR impure, missing OH)<br>[4]ionic liquid, alcohol for purification (no SI spectra)<br>[5]stoichiometric acid catalyst and organic reagent, organic solvent, chlorinated solvent reaction, (only ^{1}H, no SI spectra)<br>[6]excess acid, 18 hour heating, alcohol for purification 46% yield (no SI spectra)<br>[8]excess SDS, 8 hours, DMF for purification (no SI spectra)<br>[10]synthetic ionic liquid, organic solvent for extraction, alcohol for purification (no SI spectra)	^{1}H and ^{13}C NMR at full scale, FTIR, HRMS
**P-7**	4-NO_2-Ph	[4]ionic liquid, alcohol for purification (no SI spectra)<br>[5]stoichiometric acid catalyst and organic reagent, organic solvent, chlorinated solvent reaction, (only ^{1}H, no SI spectra)<br>[8]excess SDS, 7 hours, DMF for purification (no SI spectra)<br>[10]synthetic ionic liquid, organic solvent for extraction, alcohol for purification	^{1}H and ^{13}C NMR, FTIR, HRMS
**P-8**	3-F-Ph	Not synthesized before	^{1}H and ^{13}C NMR, FTIR, HRMS

Table S-3. Calculated energy and distance between the OH and C=O of the two pyrone rings with respect to rotation of one pyrone ring for P-1.

	Energy (kJ/mol)	Constraint(Con1) °	Distance(H25,O4)	Distance(O3,H17)
M0001	33.03	0.00	4.170	6.255
M0002	-14.80	40.00	3.582	5.992
M0003	-23.71	80.00	3.832	6.219
M0004	-20.76	120.00	4.181	6.600
M0005	11.55	160.00	6.282	5.942
M0006	3.41	200.00	5.847	1.990
M0007	-12.76	240.00	5.274	1.662
M0008	-14.58	280.00	4.910	1.621
M0009	-33.40	320.00	1.847	1.761
M0010	-24.66	360.00	3.422	2.501

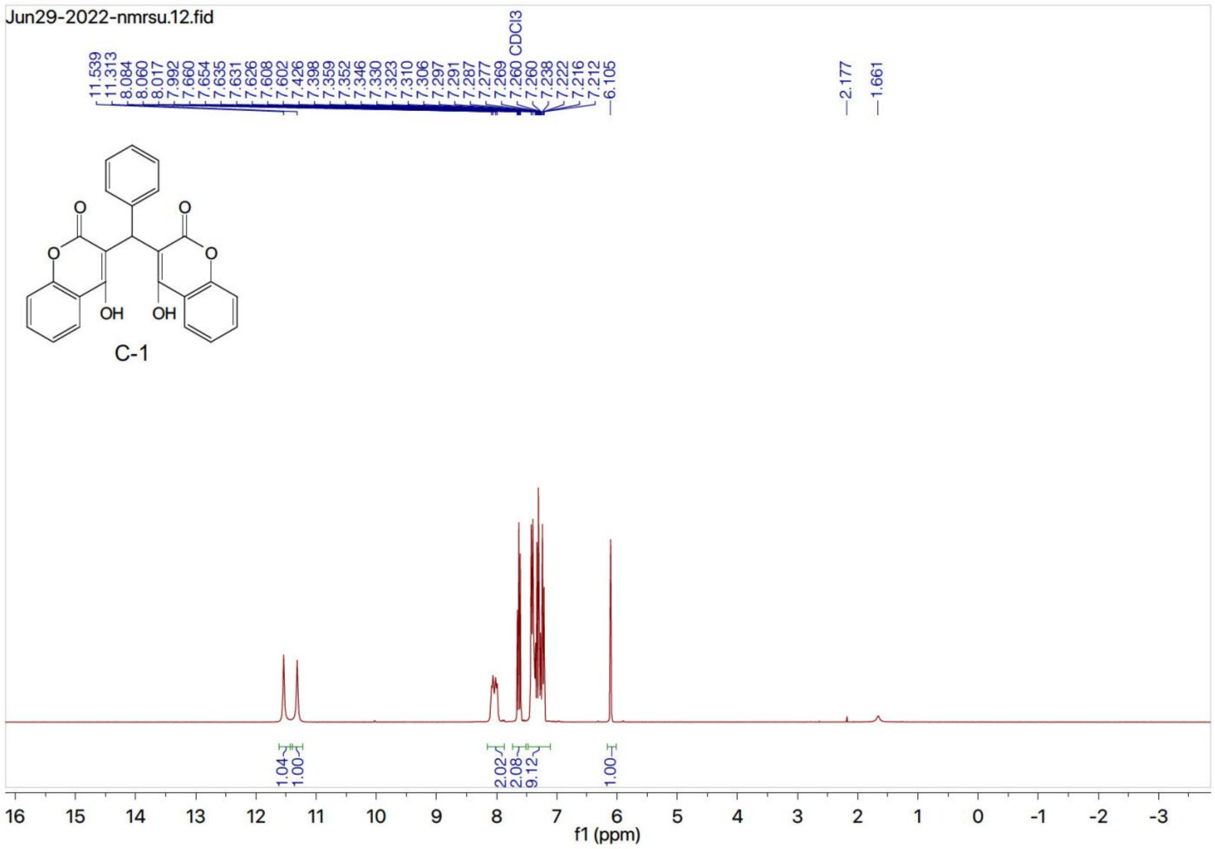

FigureS-4. ^{1}H NMR spectrum of C-1 in $CDCl_3$.

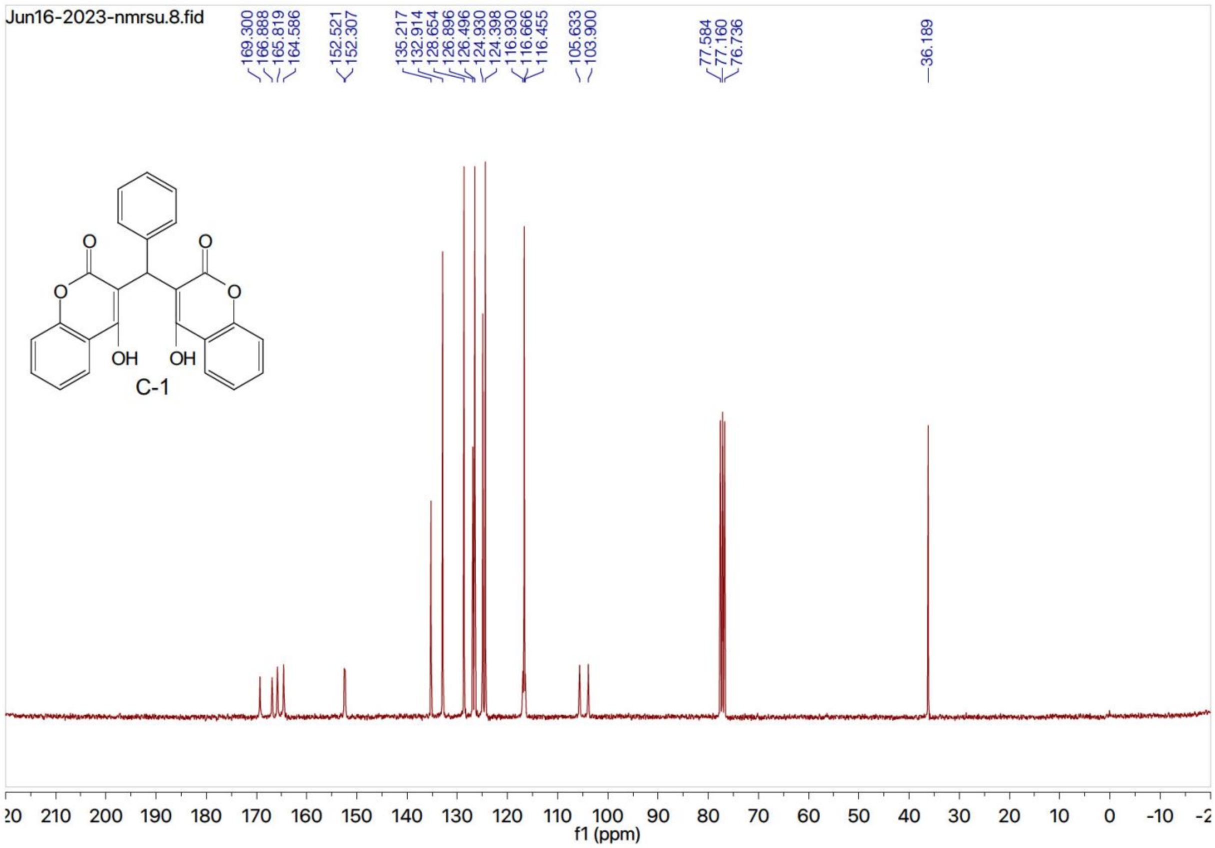

FigureS-5. ^{13}C NMR spectrum of C-1 in $CDCl_3$.

U of M

University of Minnesota Department of Chemistry
Mass Spectrometry Service Laboratory
email: chmmslab@umn.edu

Submit Sample To: Mass Spectrometry Facility
207 Pleasant St. SE
Minneapolis, MN 55455
Phone: (612)-625-8099
FAX : (612)-626-7541

Name: Julio Tapia	Phon[redacted] Date: 6/12/2023
Email: tapiahjs @ augsburg.edu Email Results? (Y) / N	FAX: FAX Results? Y / N
P.I./Advisor: Michael Wentzel	U of M Budget #
Company/University: Augsburg University	P.O.# (For non-U of M Clients) Augsburg University Chemistry Department
Shipping Address:	Billing Address: 2211 Riverside Ave, Minneapolis, MN 55454
Sample Label: **C1**	Molecular Weight: 412.40
Structural Formula or Sample Composition:	Molecular Formula: $C_{25}H_{16}O_6$
	Melting/Boiling Point:
	Solubility:
	Thermal Stability:
	Toxicity:
	Reactivity:

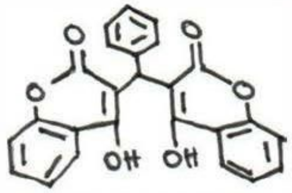

Chromatography Conditions:

Special Sample Considerations:

Analysis Requested

	EI	CI	MALDI	ESI
Low Resolution Nominal Mass				
High Resolution Accurate Mass				✓
+Ve				
-Ve				
GCMS				
LCMS				

Instrument Used		Conditions Used
Finnigan MAT 95		Source Temp:
Bruker Reflex III		Acc. Voltage:
Bruker BioTOF II		Resolution:
Waters Triple Quad		Scan Range:
Waters Synapt G2		Gas Used:

Operator Comments

POS mode, MeOH solvent
PEG400 calibrant
M+Na theoretical 435.0839
observed 435.0823
error 3.66 ppm

Log #:	Analyst:	Analysis Date:	Analyses Run:	Total Cost:

130193
C2
oesi
madeline honig
6/15/2023 3:00:03 PM

FigureS-6. HRMS results of C-1.

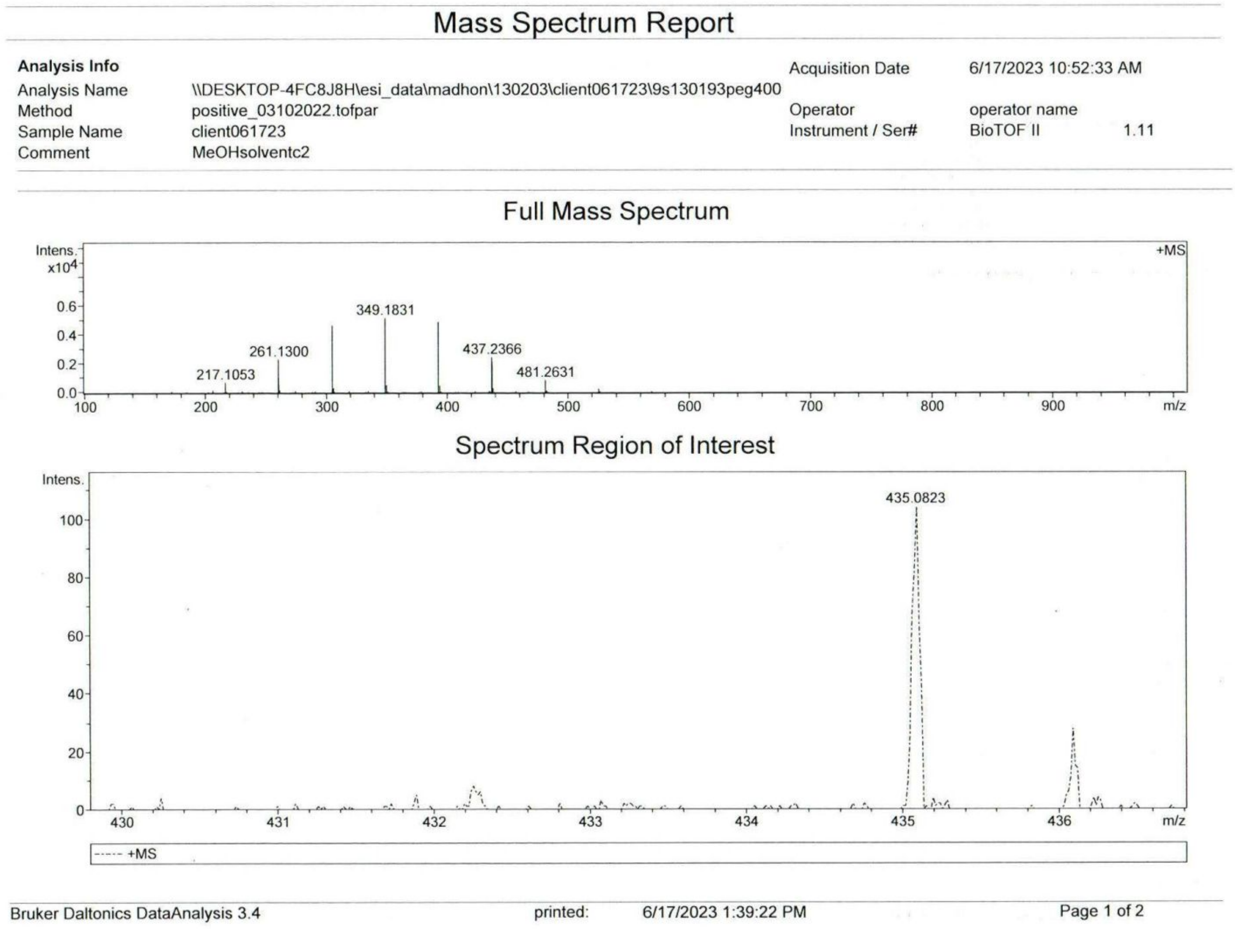

FigureS-7. HRMS results of C-1 spectrum.

Mass Spectrum Report

Elemental Composition Report

Generate Molecular Formula Parameter

Formula, min.	C0H16Na1O6						
Formula, max.	C25H16O6Na						
Measured m/z	435.082	Tolerance	10	ppm		Charge	1
Check Valence	no	Minimum	0			Maximum	0
Nirogen Rule	no	Electron Configuration		both			
Filter H/C Ratio	no	Minimum	0			Maximum	3
Estimate Carbon	yes						

Sum Formula	Sigma	m/z	Err [ppm]	Mean Err [ppm]	Err [mDa]	rdb	N Rule	e⁻
C 25 H 16 Na 1 O 6	0.161	435.0839	3.66	3.59	1.59	17.50	ok	even

Mass Spectrum Peak List

#	m/z	Area	Res.	S/N
1	207.0010	4	10303	5.5
2	217.1053	22	7143	20.5
3	231.1205	3	11496	3.1
4	261.1300	81	8133	58.1
5	262.1339	6	7753	4.0
6	275.1439	4	9710	3.0
7	305.1568	177	8446	136.6
8	306.1598	13	7902	9.3
9	335.1671	5	8142	2.9
10	349.1831	248	7889	137.4
11	350.1855	23	8360	13.8
12	393.2096	235	8488	142.1
13	394.2117	23	8732	14.7
14	435.0823	6	7561	4.6
15	437.2366	134	8741	110.5
16	438.2394	15	7620	10.8
17	481.2631	46	8948	60.2
18	525.2868	13	6273	21.7

Bruker Daltonics DataAnalysis 3.4 printed: 6/17/2023 1:39:22 PM Page 2 of 2

FigureS-8. HRMS results of C-1 peak list.

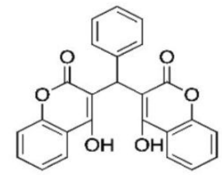

C-1

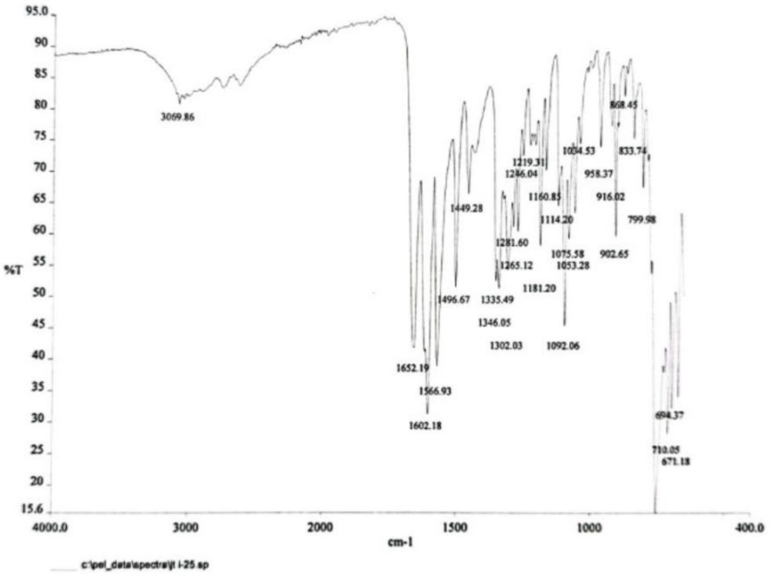

FigureS-9. IR spectrum of C-1.

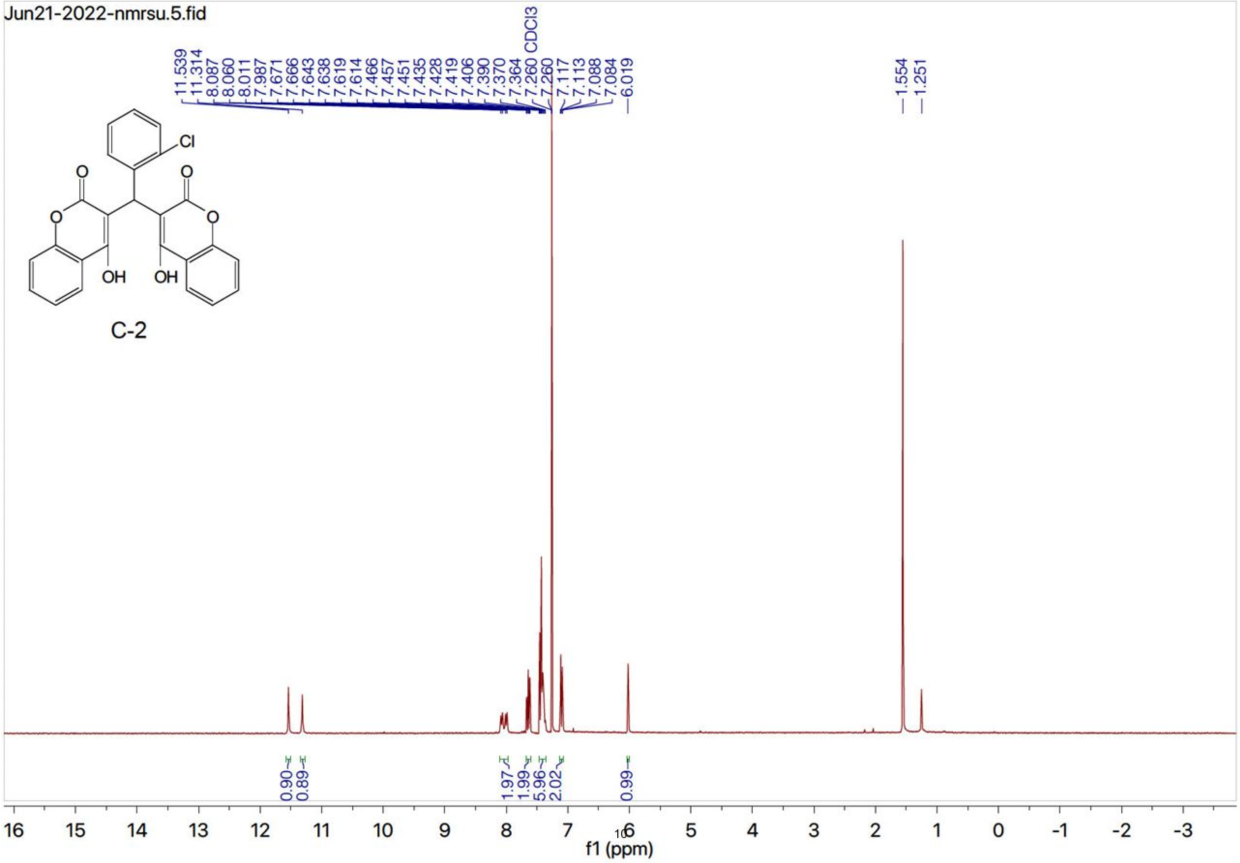

FigureS-10. ^{1}H NMR spectrum of C-2 in $CDCl_3$.

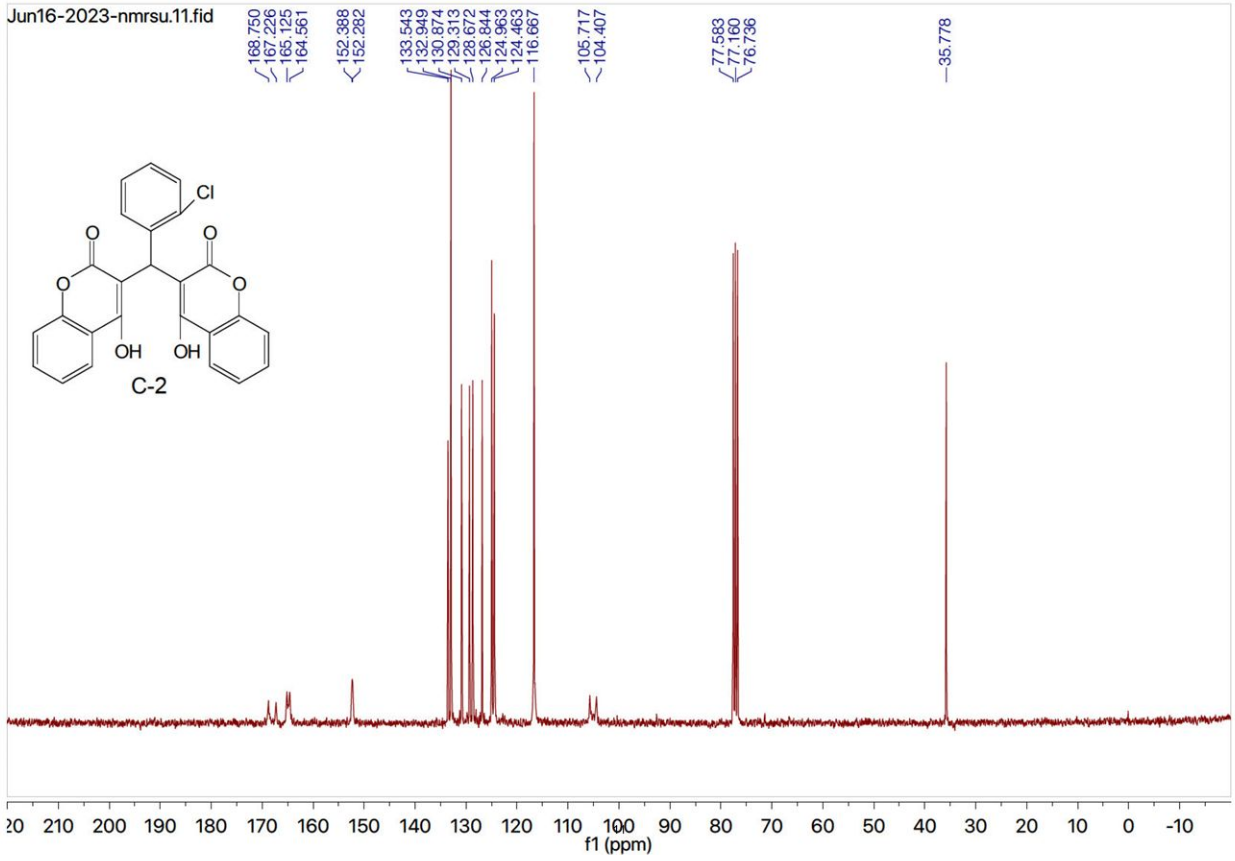

FigureS-11. ^{13}C NMR spectrum of C-1 in $CDCl_3$.

U of M
University of Minnesota Department of Chemistry
Mass Spectrometry Service Laboratory
email: chmmslab@umn.edu

Submit Sample To: Mass Spectrometry Facility
207 Pleasant St. SE
Minneapolis, MN 55455
Phone: (612)-625-8099
FAX : (612)-626-7541

Name: Julio Tapia	Phone: [redacted] Date: 6/12/2023
Email: tapiahjs@augsburg.edu Email Results? Y / N	FAX: FAX Results? Y / N
P.I./Advisor: Michael Wentzel	U of M Budget #
Company/University: Augsburg University	P.O.# (For non-U of M Clients) Augsburg University Chemistry Department
Shipping Address:	Billing Address: 2211 Riverside Ave, Minneapolis, MN 55454
Sample Label: C2	Molecular Weight: 446.84
Structural Formula or Sample Composition:	Molecular Formula: $C_{25}H_{15}ClO_6$
	Melting/Boiling Point:
	Solubility:
	Thermal Stability:
	Toxicity:
	Reactivity:

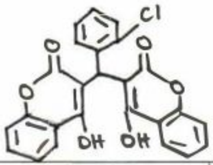

Chromatography Conditions:

Special Sample Considerations:

Analysis Requested

	EI	CI	MALDI	ESI
Low Resolution Nominal Mass				
High Resolution Accurate Mass				✓
+Ve				
-Ve				
GCMS				
LCMS				

Instrument Used		Conditions Used
Finnigan MAT 95		Source Temp:
Bruker Reflex III		Acc. Voltage:
Bruker BioTOF II		Resolution:
Waters Triple Quad		Scan Range:
Waters Synapt G2		Gas Used:

Operator Comments
Neg mode, MeOH/DCM solvent
Na-TFA calibrant
M−1 theoretical 445.0484
observed 445.0489
error -0.94 ppm

Log #:	Analyst:	Analysis Date:	Analyses Run:	Total Cost:

130188
C4
oesi
madeline honig
6/15/2023 2:40:15 PM

12

FigureS-12. HRMS results of C-2

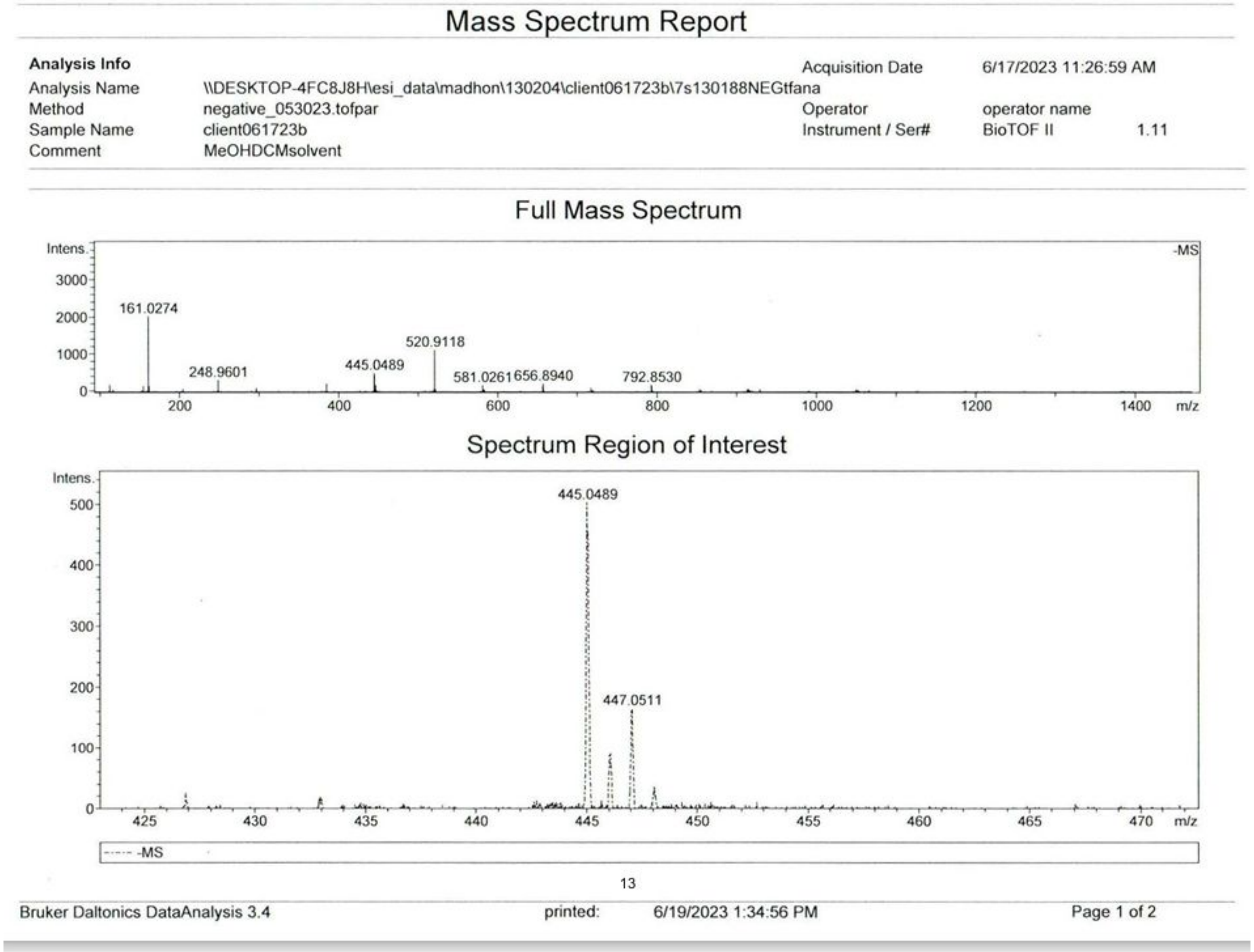

FigureS-13. HRMS results of C-2 spectrum.

Mass Spectrum Report

Elemental Composition Report

Generate Molecular Formula Parameter

Formula, min.	C23H14Cl1O6						
Formula, max.	C25H14ClO6						
Measured m/z	445.049	Tolerance	15	ppm		Charge	-1
Check Valence	no	Minimum	0			Maximum	0
Nirogen Rule	no	Electron Configuration		both			
Filter H/C Ratio	no	Minimum	0			Maximum	3
Estimate Carbon	yes						

Sum Formula	Sigma	m/z	Err [ppm]	Mean Err [ppm]	Err [mDa]	rdb	N Rule	e⁻
C 25 H 14 Cl 1 O 6	0.131	445.0484	-0.94	-3.14	-0.42	18.50	ok	even

Mass Spectrum Peak List

14

#	m/z	Area	Res.	S/N
1	112.9861	6	3394	6.5
2	154.9724	6	3644	5.9
3	161.0274	96	3458	83.9
4	162.0308	6	4055	5.8
5	248.9601	23	3361	25.6
6	384.9307	23	3499	15.2
7	445.0489	64	3672	32.3
8	447.0511	20	3980	10.7
9	520.9118	151	4173	86.6
10	581.0261	27	3159	13.5
11	656.8940	37	4133	16.6
12	792.8530	30	4436	15.9

Bruker Daltonics DataAnalysis 3.4 printed: 6/19/2023 1:34:56 PM Page 2 of 2

FigureS-14. HRMS results of C-2 peak list.

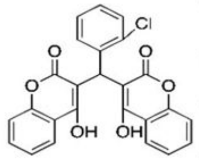

C-2

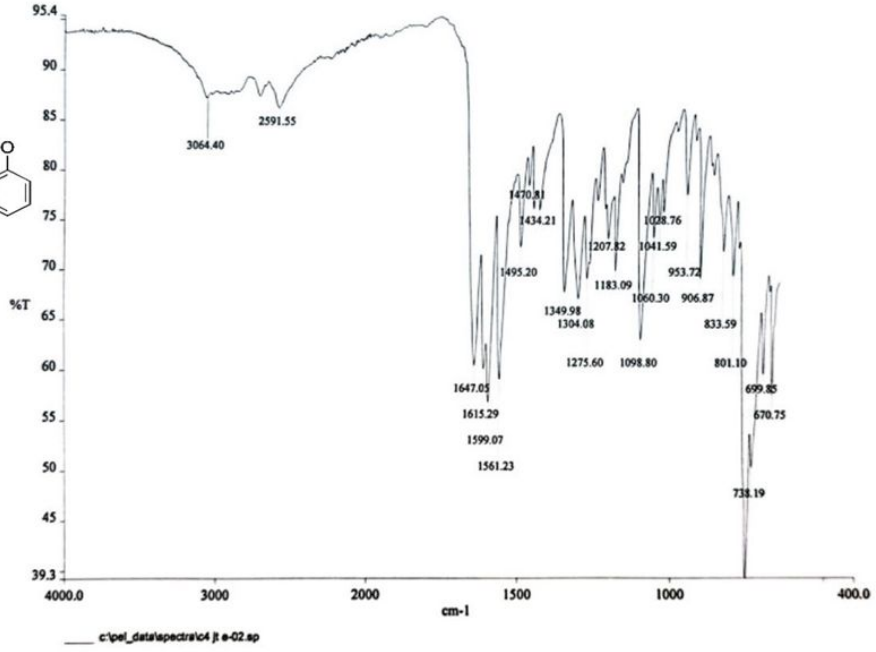

15

FigureS-15. IR spectrum of C-2.

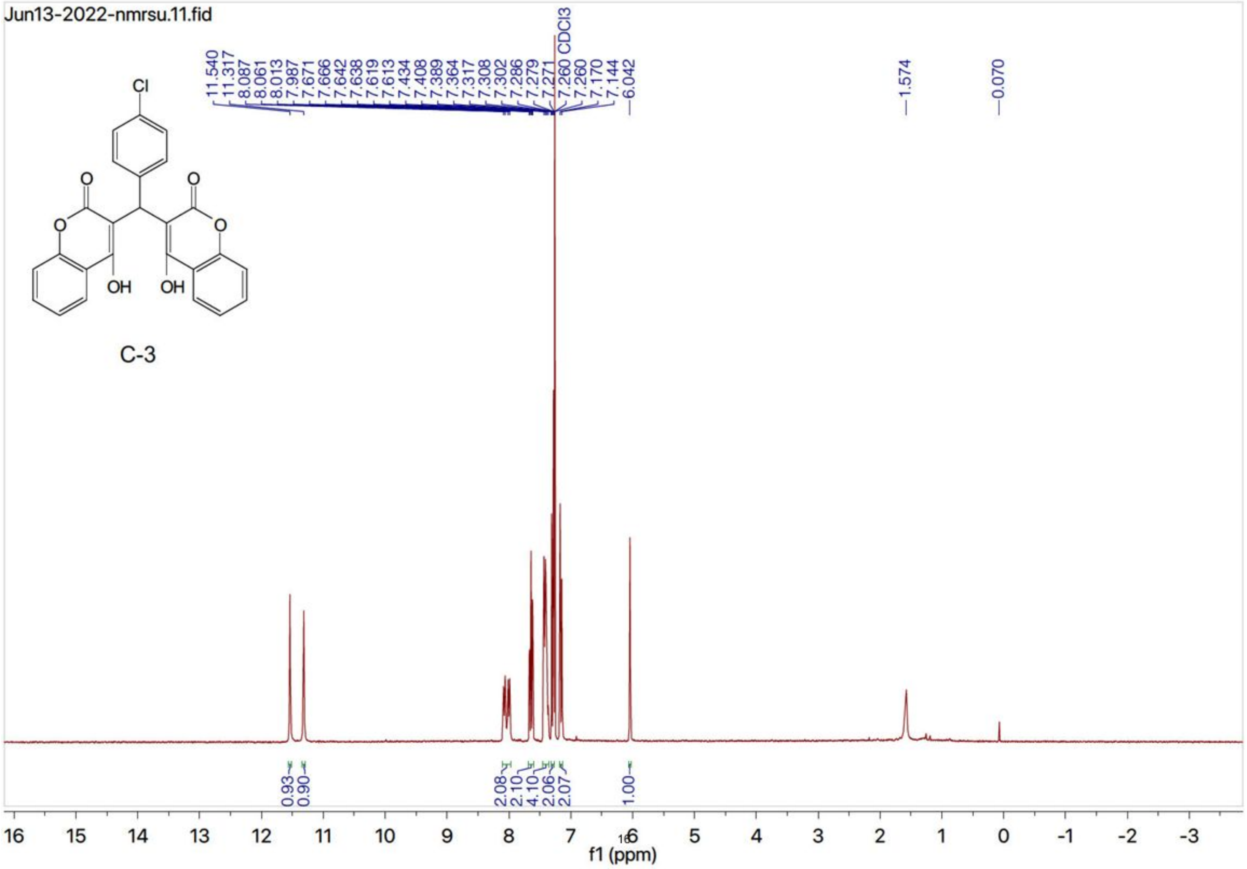

FigureS-16. ^{1}H NMR spectrum of C-3 in CDCl3.

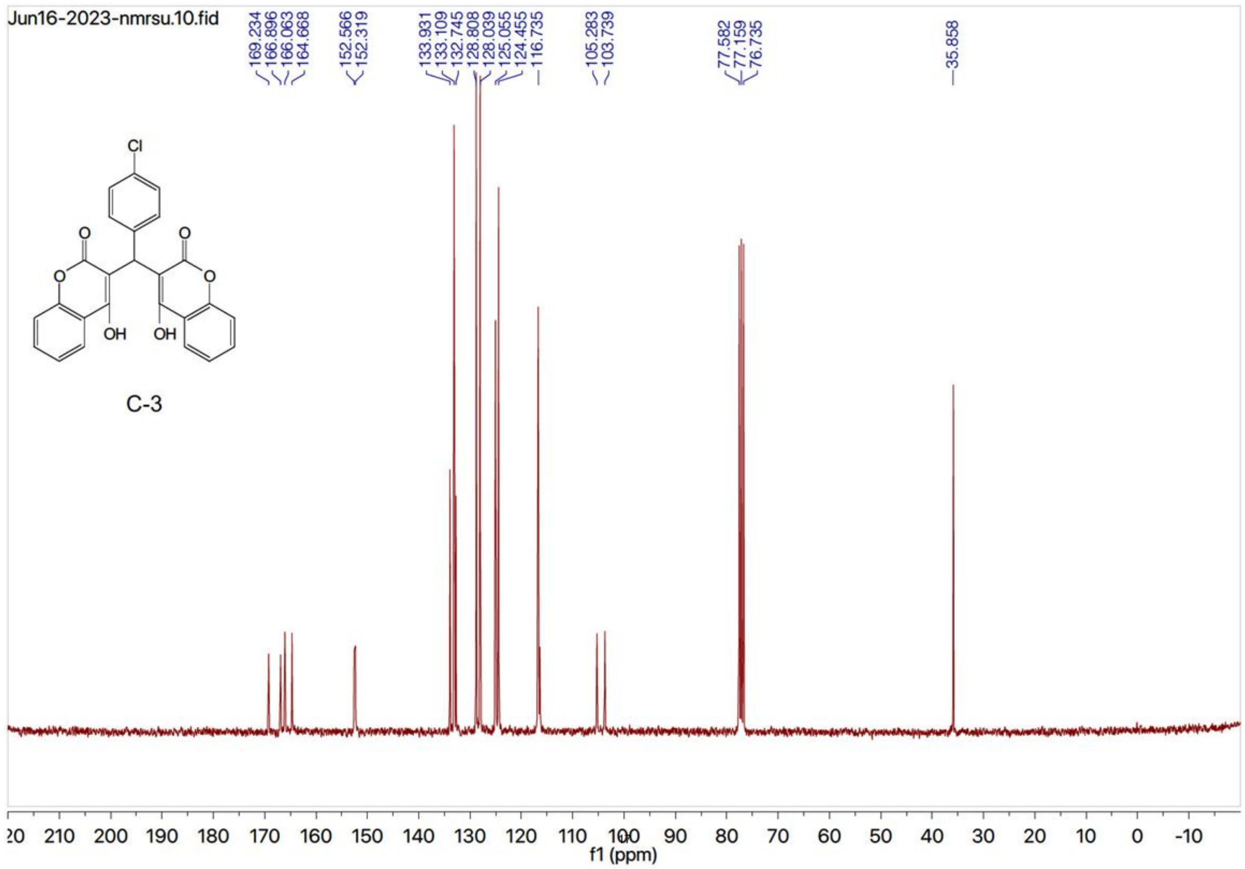

FigureS-17. 13C NMR spectrum of C-3 in CDCl3.

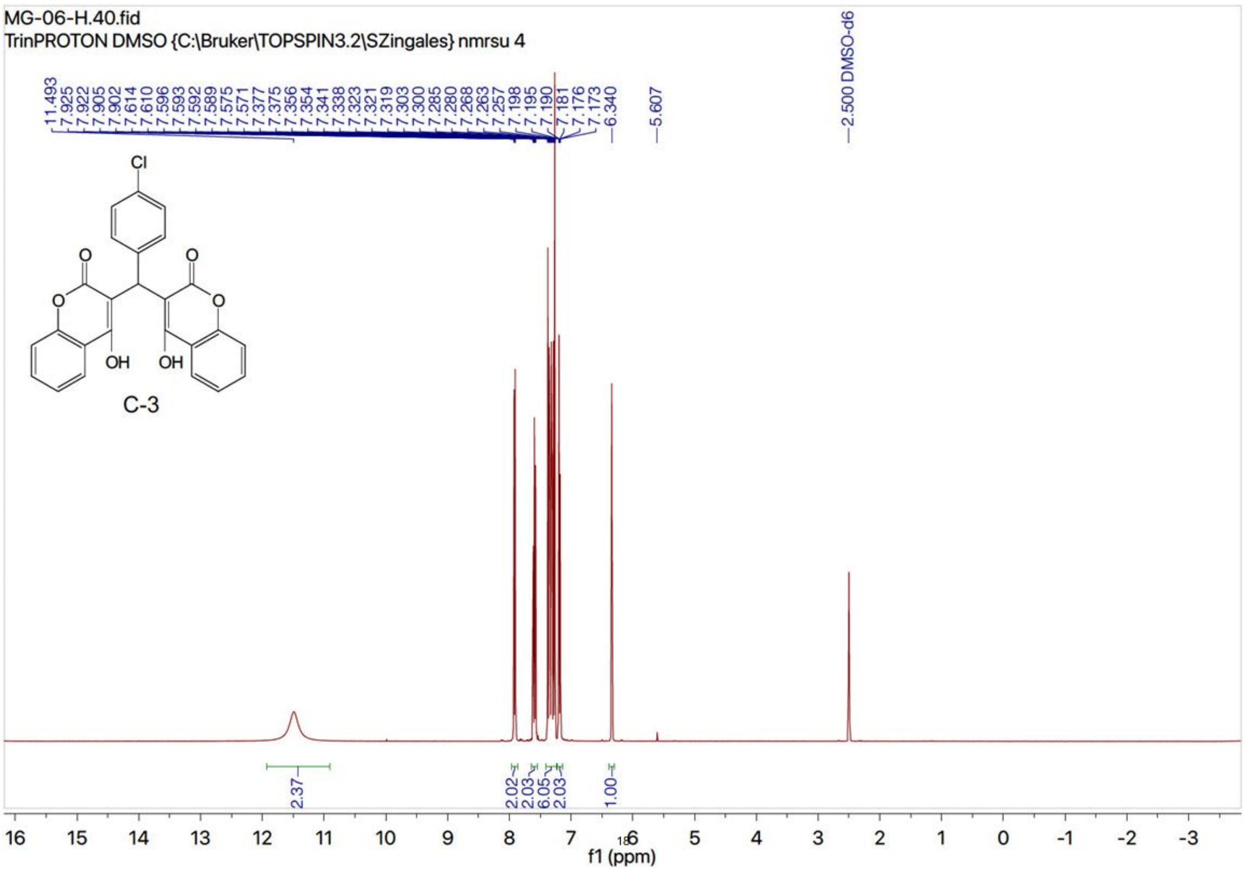

FigureS-18. DMSO-d_6 ^{1}H NMR, ^{13}C NMR spectra of C-3

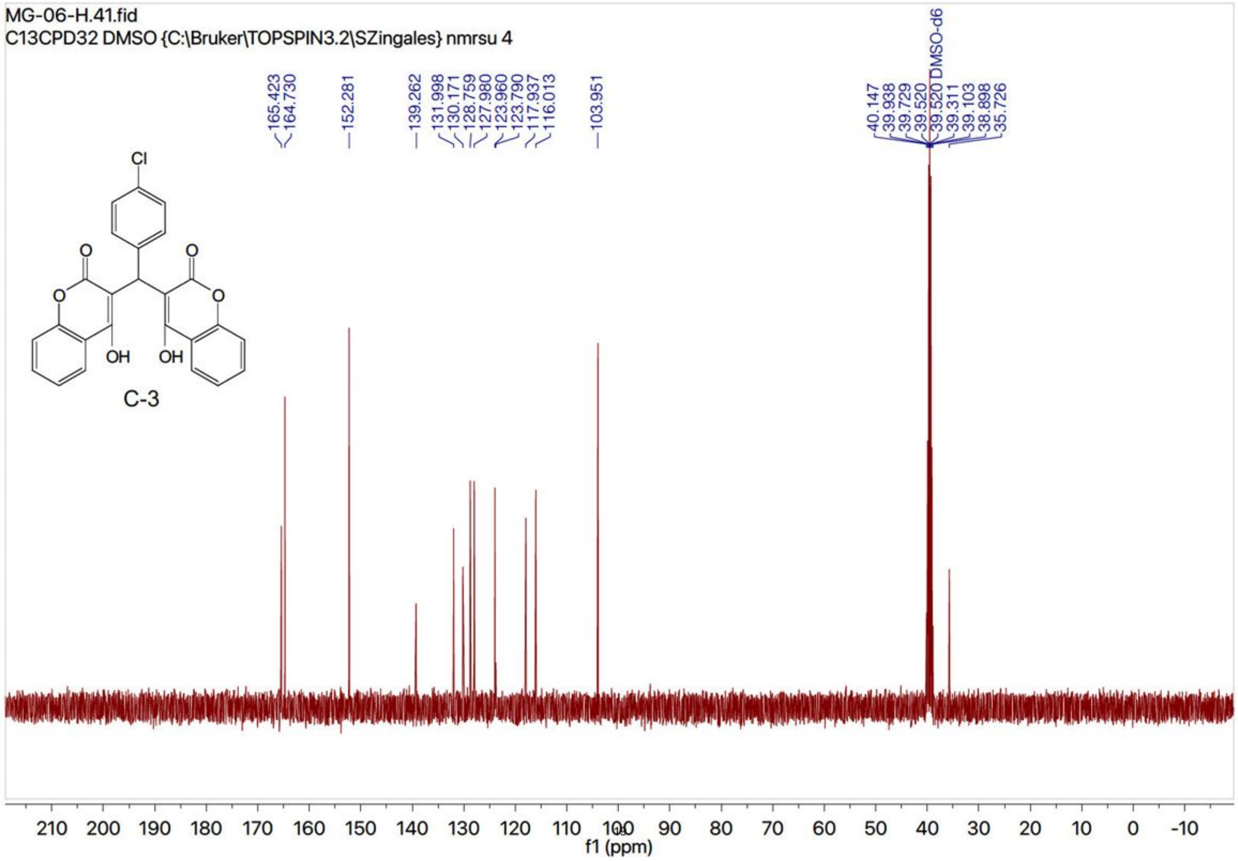

FigureS-19. DMSO-d_6 ^{1}H NMR, ^{13}C NMR spectra of C-3

U of M

University of Minnesota Department of Chemistry
Mass Spectrometry Service Laboratory
email: chmmslab@umn.edu

Submit Sample To: Mass Spectrometry Facility
207 Pleasant St. SE
Minneapolis, MN 55455
Phone: (612)-625-8099
FAX : (612)-626-7541

Name: Julio Tapia	Phone [redacted] Date: 6/12/2023
Email: tapiahjs@augsburg.edu Email Results? Y / N	FAX: FAX Results? Y / N
P.I./Advisor: Michael Wentzel	U of M Budget #
Company/University: Augsburg University	P.O.# (For non-U of M Clients) Augsburg University Chemistry Department
Shipping Address:	Billing Address: 2211 Riverside Ave, Minneapolis, MN 55454
Sample Label: C3	Molecular Weight: 446.84
Structural Formula or Sample Composition:	Molecular Formula: $C_{25}H_{15}ClO_6$
	Melting/Boiling Point:
	Solubility:
	Thermal Stability:
	Toxicity:
	Reactivity:

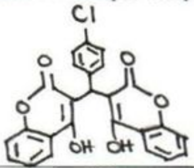

Chromatography Conditions:

Special Sample Considerations:

Analysis Requested

	EI	CI	MALDI	ESI
Low Resolution Nominal Mass				
High Resolution Accurate Mass				√
+Ve				
-Ve				
GCMS				
LCMS				

Instrument Used		Conditions Used	Operator Comments
Finnigan MAT 95		Source Temp:	Neg mode, MeOH/DCM solvent
Bruker Reflex III		Acc. Voltage:	NaTFA Neg calibrant
Bruker BioTOF II		Resolution:	M-1 theoretical 445.0484
Waters Triple Quad		Scan Range:	observed 445.0464
Waters Synapt G2		Gas Used:	error 4.47 ppm

Log #:	Analyst:	Analysis Date:	Analyses Run:	Total Cost:

130187
C3
oesi
madeline honig
6/15/2023 2:38:39 PM

20

FigureS-20. HRMS results of C-3

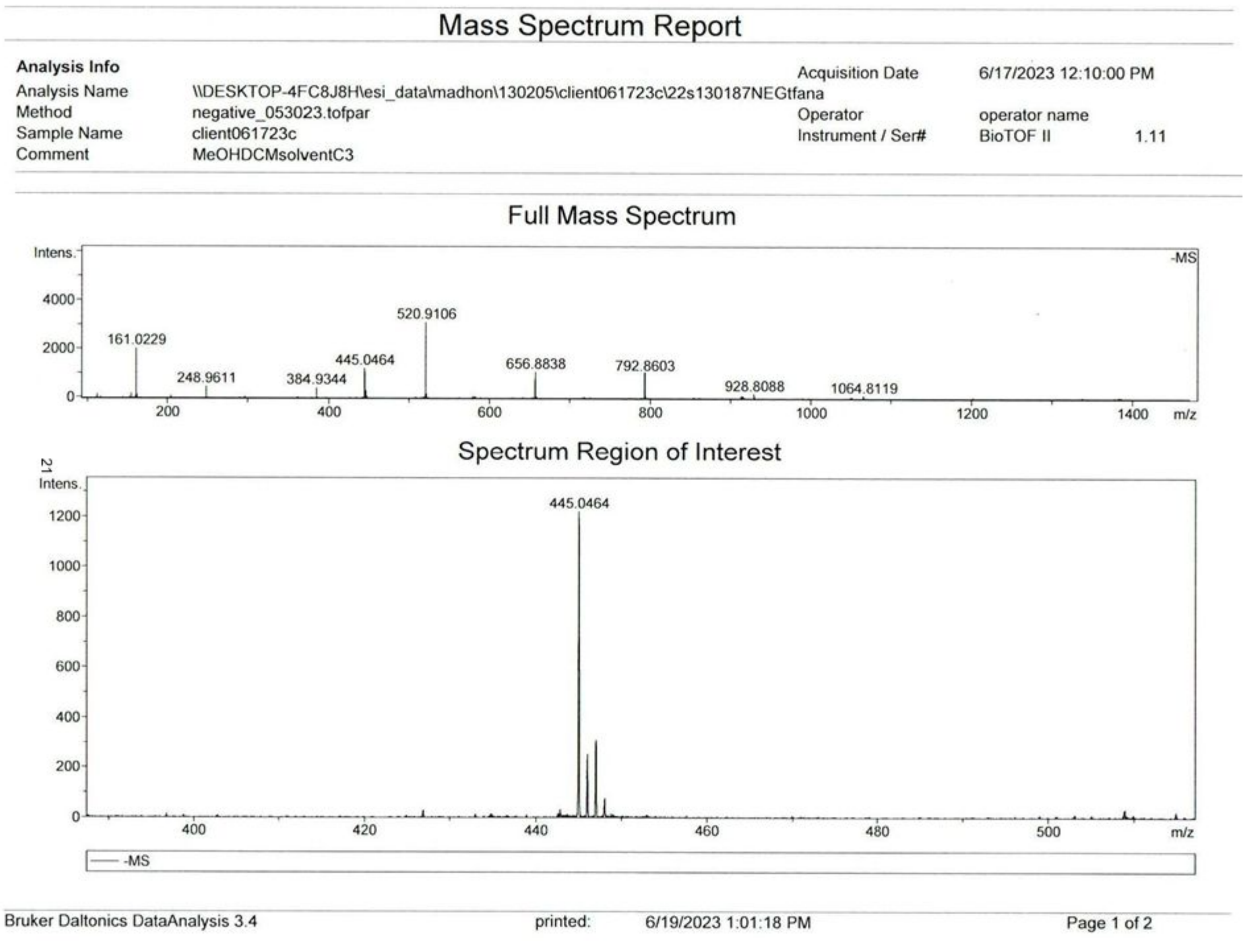

FigureS-21. HRMS results of C-3 spectrum.

Mass Spectrum Report

Elemental Composition Report

Generate Molecular Formula Parameter

Formula, min.	C13H14Cl1O6						
Formula, max.	C25H14ClO6						
Measured m/z	445.046	Tolerance	20	ppm		Charge	-1
Check Valence	no	Minimum	0			Maximum	0
Nirogen Rule	no	Electron Configuration		both			
Filter H/C Ratio	no	Minimum	0			Maximum	3
Estimate Carbon	yes						

Sum Formula	Sigma	m/z	Err [ppm]	Mean Err [ppm]	Err [mDa]	rdb	N Rule	e⁻
C 25 H 14 Cl 1 O 6	0.073	445.0484	4.47	5.29	1.99	18.50	ok	even

Mass Spectrum Peak List

22

#	m/z	Area	Res.	S/N
1	112.9854	4	4807	7.4
2	154.9742	7	4121	9.9
3	161.0229	71	4978	111.3
4	162.0278	4	5210	7.4
5	248.9611	29	4574	43.1
6	384.9344	39	4166	31.6
7	445.0464	138	4042	73.2
8	446.0489	25	4540	15.2
9	447.0428	36	3801	18.6
10	520.9106	424	4161	184.3
11	521.9148	17	4253	7.7
12	656.8838	175	4389	90.6
13	792.8603	218	3784	82.7
14	928.8088	47	3498	15.6
15	1064.8119	35	2090	10.0

Bruker Daltonics DataAnalysis 3.4 printed: 6/19/2023 1:01:18 PM Page 2 of 2

FigureS-22. HRMS results of C-3 peak list.

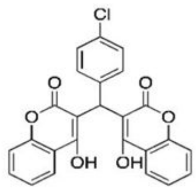

C-3

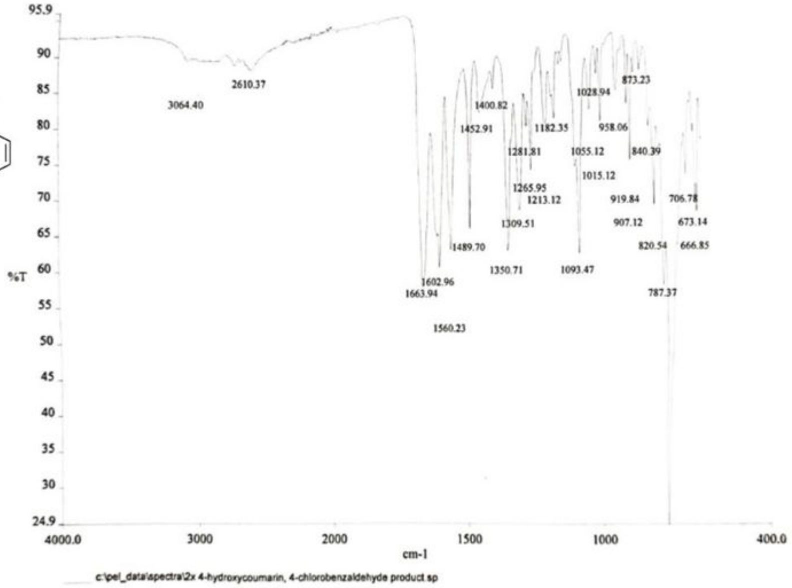

23

FigureS-23. Figure S-8. IR spectrum of C-3.

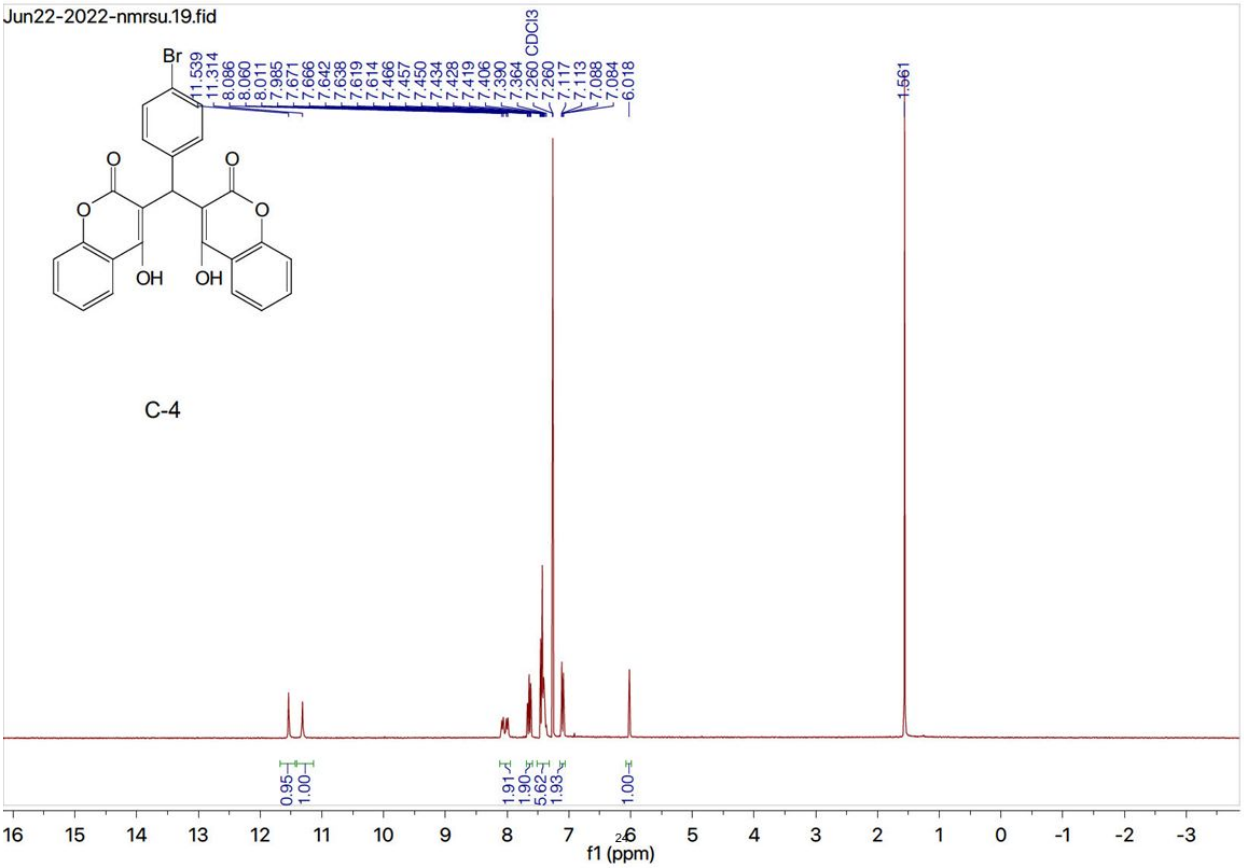

FigureS-24. ^{1}H NMR spectrum of C-4 in $CDCl_3$

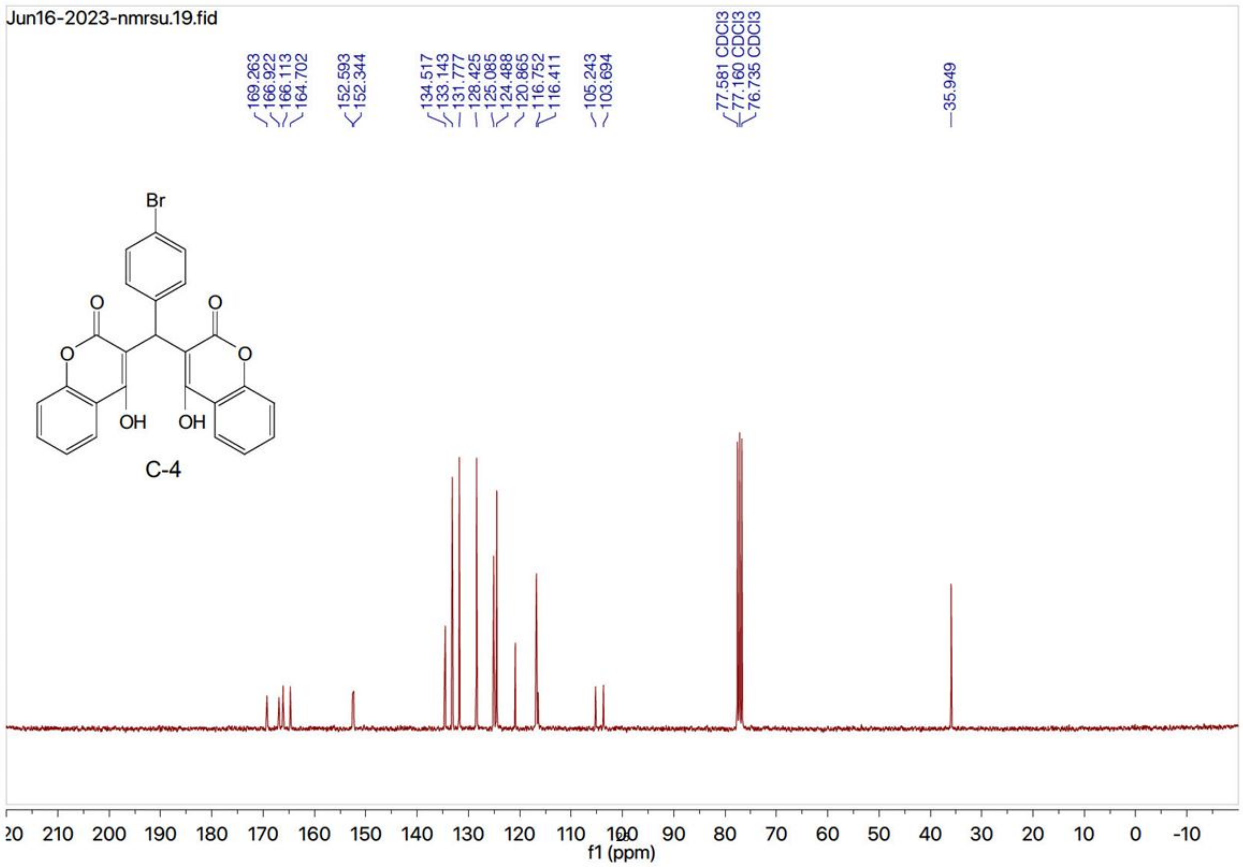

FigureS-25. ^{13}C NMR spectrum of C-4 in $CDCl_3$.

U of M

University of Minnesota Department of Chemistry
Mass Spectrometry Service Laboratory
email: chmmslab@umn.edu

Submit Sample To: Mass Spectrometry Facility
207 Pleasant St. SE
Minneapolis, MN 55455
Phone: (612)-625-8099
FAX: (612)-626-7541

Name: Julio Tapia	Phone: [redacted] Date: 6/12/2023
Email: tapiahj5@augsburg.edu Email Results? Y/N	FAX: FAX Results? Y / N
P.I./Advisor: Michael Wentzel	U of M Budget #
Company/University: Augsburg University	P.O.# (For non-U of M Clients) Augsburg University Chemistry Department
Shipping Address:	Billing Address: 2211 Riverside Ave, Minneapolis, MN 55454
Sample Label: C8 C4	Molecular Weight: 491.29
Structural Formula or Sample Composition:	Molecular Formula: $C_{25}H_{15}BrO_6$
	Melting/Boiling Point:
	Solubility:
	Thermal Stability:
	Toxicity:
	Reactivity:

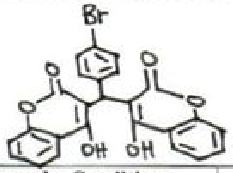

Chromatography Conditions:

Special Sample Considerations:

Analysis Requested	EI	CI	MALDI	ESI
Low Resolution Nominal Mass				
High Resolution Accurate Mass				√
+Ve				
-Ve				
GCMS				
LCMS				

Instrument Used		Conditions Used
Finnigan MAT 95		Source Temp:
Bruker Reflex III		Acc. Voltage:
Bruker BioTOF II		Resolution:
Waters Triple Quad		Scan Range:
Waters Synapt G2		Gas Used:

Operator Comments

Neg mode. MeOH/DCM solvent
Na-TFA calibrant
M-1 theoretical 455.9979
observed 455.9995
error -3.53 ppm

Log #:	Analyst:	Analysis Date:	Analyses Run:	Total Cost:

130192
C8
oesi
madeline honig
6/15/2023 2:46:25 PM

26

FigureS-26. HRMS results of C-4

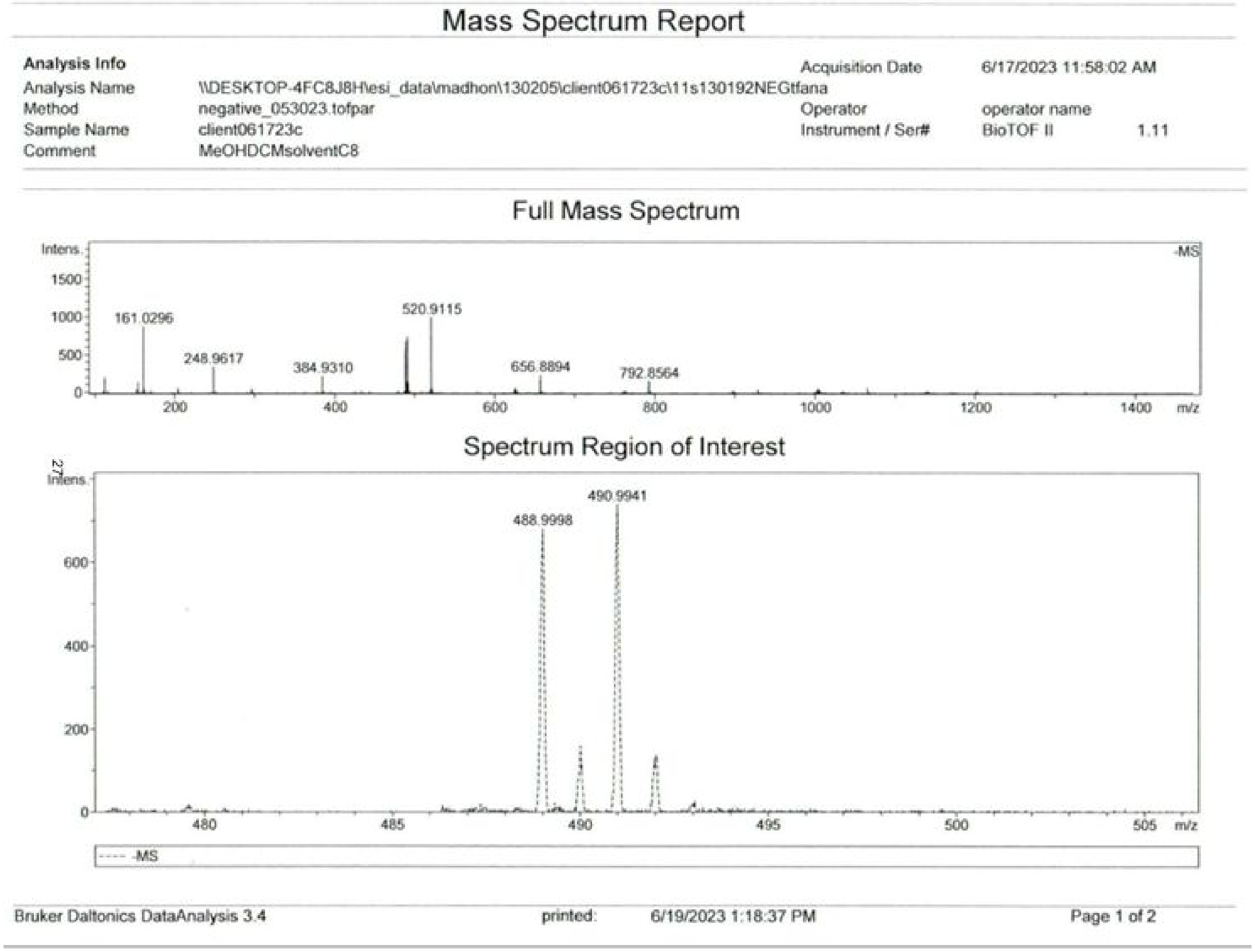

FigureS-27. HRMS results of C-4 spectrum.

Mass Spectrum Report

Elemental Composition Report

Generate Molecular Formula Parameter

Formula, min.	C15H14Br1O6						
Formula, max.	C25H14BrO6						
Measured m/z	489	Tolerance	20	ppm		Charge	-1
Check Valence	no	Minimum	0			Maximum	0
Nirogen Rule	no	Electron Configuration		both			
Filter H/C Ratio	no	Minimum	0			Maximum	3
Estimate Carbon	yes						

Sum Formula	Sigma	m/z	Err [ppm]	Mean Err [ppm]	Err [mDa]	rdb	N Rule	e⁻
C 25 H 14 Br 1 O 6	0.056	488.9979	-3.83	-1.55	-1.87	18.50	ok	even

Mass Spectrum Peak List

#	m/z	Area	Res.	S/N
1	112.9945	5	5101	10.0
2	154.9809	5	4330	7.3
3	161.0296	34	4321	46.1
4	248.9617	19	5271	26.7
5	384.9310	19	4490	24.3
6	488.9998	83	4296	46.4
7	490.0039	18	3845	10.8
8	490.9941	90	4112	49.8
9	492.0070	18	3140	9.1
10	520.9115	130	4409	64.8
11	656.8894	40	4412	20.5
12	792.8564	33	4247	13.5

Bruker Daltonics DataAnalysis 3.4 printed: 6/19/2023 1:18:37 PM Page 2 of 2

FigureS-28. HRMS results of C-4 peak list.

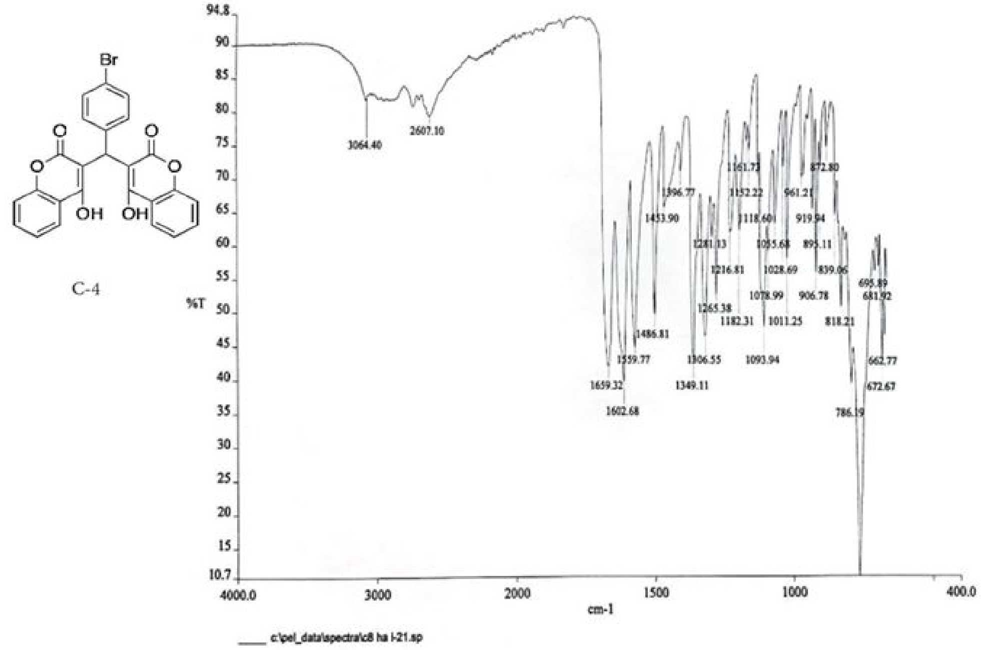

FigureS-29. IR spectrum of C-4.

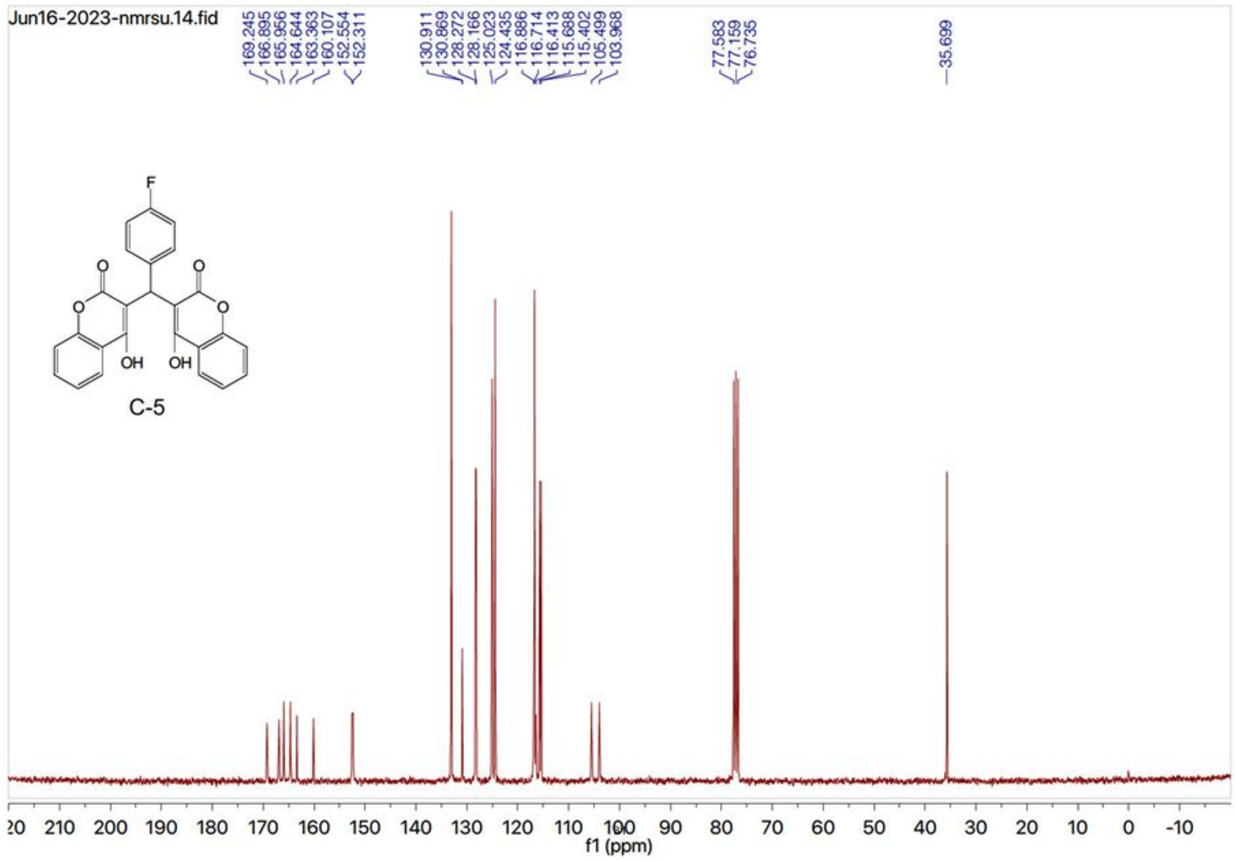

FigureS-30. ^{1}H NMR spectrum of C-5 in $CDCl_3$.

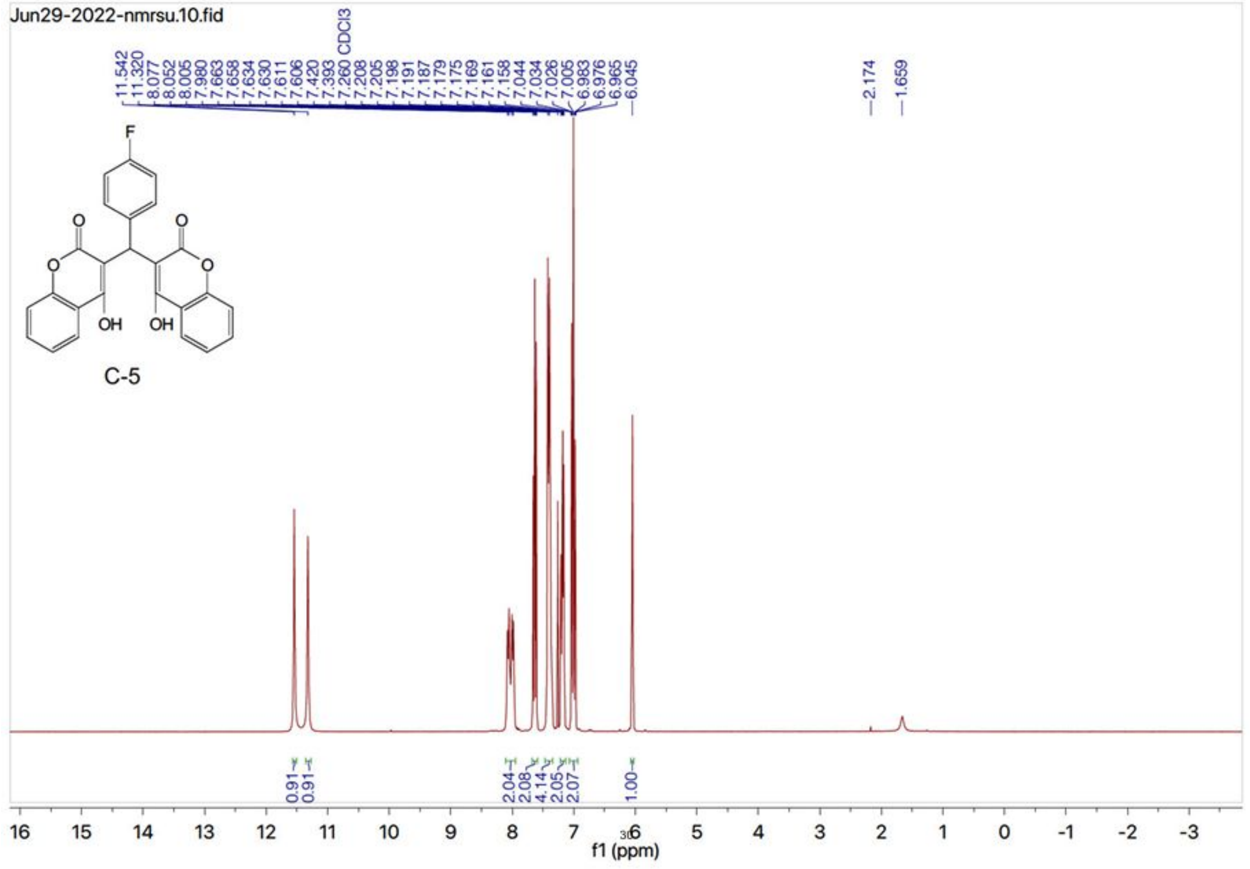

FigureS-31. **^{13}C NMR spectrum of C-5 in $CDCl_3$.**

U of M
University of Minnesota Department of Chemistry
Mass Spectrometry Service Laboratory
email: chmmslab@umn.edu

Submit Sample To: Mass Spectrometry Facility
207 Pleasant St. SE
Minneapolis, MN 55455
Phone: (612)-625-8099
FAX: (612)-626-7541

Name: Julio Tapia	Phone: [redacted] Date: 6/12/2023
Email: tapiahjs@augsburg.edu Email Results? Y / N	FAX: FAX Results? Y / N
P.I./Advisor: Michael Wentzel	U of M Budget #
Company/University: Augsburg University	P.O.# (For non-U of M Clients) Augsburg University Chemistry Department
Shipping Address:	Billing Address: 2211 Riverside Ave, Minneapolis, MN 55454
Sample Label: C5	Molecular Weight: 430.39
Structural Formula or Sample Composition:	Molecular Formula: $C_{25}H_{15}FO_6$
	Melting/Boiling Point:
	Solubility:
	Thermal Stability:
	Toxicity:
	Reactivity:

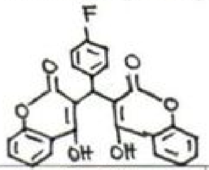

Chromatography Conditions:

Special Sample Considerations:

Analysis Requested

	EI	CI	MALDI	ESI
Low Resolution Nominal Mass				
High Resolution Accurate Mass				√
+Ve				
-Ve				
GCMS				
LCMS				

Instrument Used		Conditions Used	Operator Comments
Finnigan MAT 95		Source Temp:	NEG mode, MeOH / DCM solvent
Bruker Reflex III		Acc. Voltage:	TFA-Na calibrant
Bruker BioTOF II		Resolution:	M-1 theoretical 429.0780
Waters Triple Quad		Scan Range:	observed 429.0773
Waters Synapt G2		Gas Used:	error 1.66 ppm

Log #:	Analyst:	Analysis Date:	Analyses Run:	Total Cost:

130189
C5
oesi
madeline honig
6/15/2023 2:43:17 PM

32

FigureS-32. HRMS results of C-5

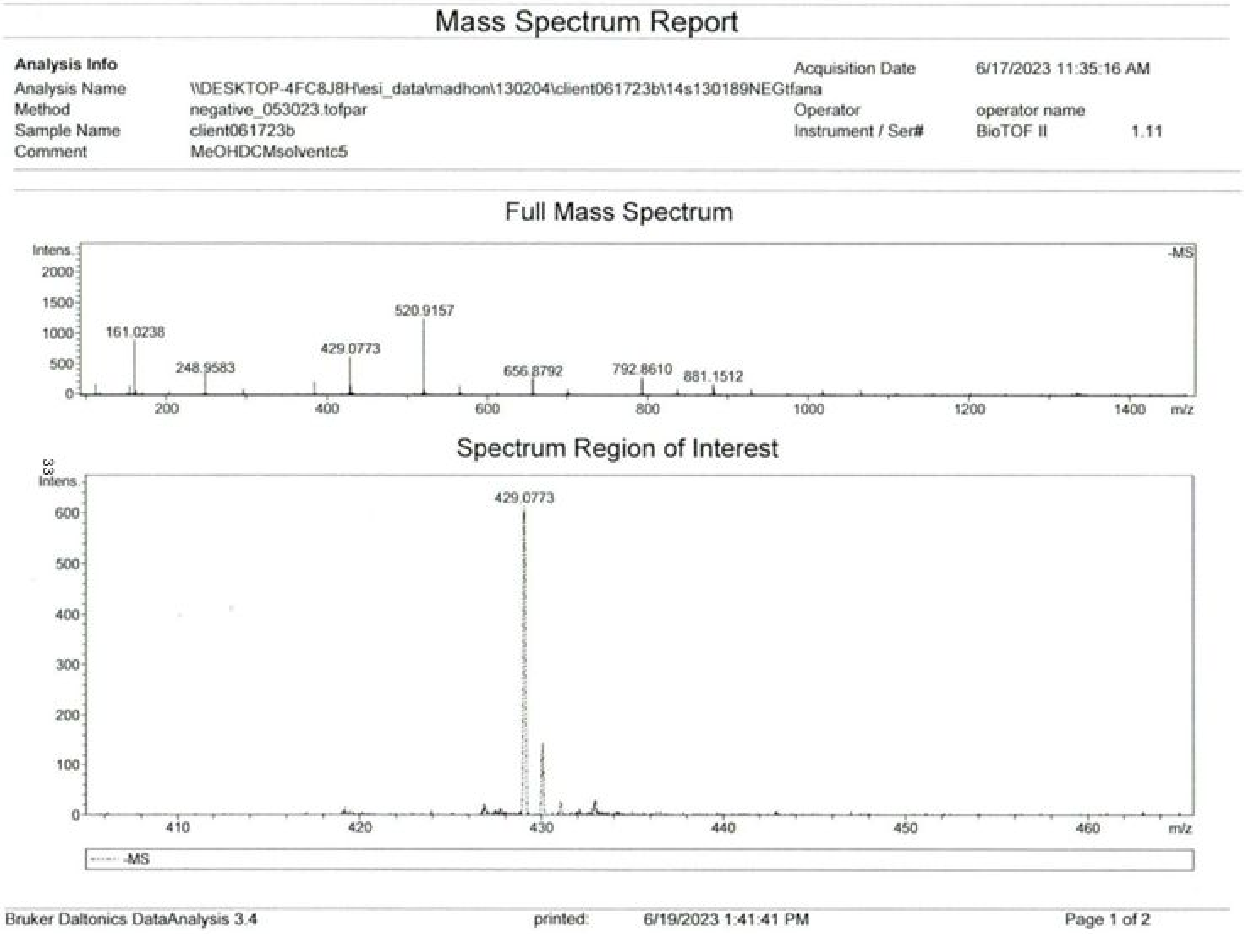

FigureS-33. HRMS results of C-5 spectrum.

Mass Spectrum Report

Elemental Composition Report

Generate Molecular Formula Parameter

Formula, min.	C15H14F1O6						
Formula, max.	C25H14FO6						
Measured m/z	429.077	Tolerance	15	ppm		Charge	-1
Check Valence	no	Minimum	0			Maximum	0
Nirogen Rule	no	Electron Configuration		both			
Filter H/C Ratio	no	Minimum	0			Maximum	3
Estimate Carbon	yes						

Sum Formula	Sigma	m/z	Err [ppm]	Mean Err [ppm]	Err [mDa]	rdb	N Rule	e⁻
C 25 H 14 F 1 O 6	0.037	429.0780	1.66	2.68	0.71	18.50	ok	even

Mass Spectrum Peak List

#	m/z	Area	Res.	S/N
1	112.9861	5	4186	9.1
2	154.9722	6	3573	8.0
3	161.0238	40	3691	49.7
4	248.9583	22	3583	21.5
5	384.9354	22	3975	14.6
6	429.0773	77	3635	36.9
7	430.0784	16	4089	8.6
8	520.9157	168	3008	96.8
9	565.0509	21	3580	12.0
10	656.8792	53	2853	17.8
11	792.8610	60	3518	27.7
12	881.1512	20	4955	13.6

Bruker Daltonics DataAnalysis 3.4 printed: 6/19/2023 1:41:41 PM Page 2 of 2

FigureS-34. HRMS results of C-5 peak list.

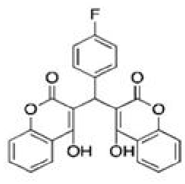

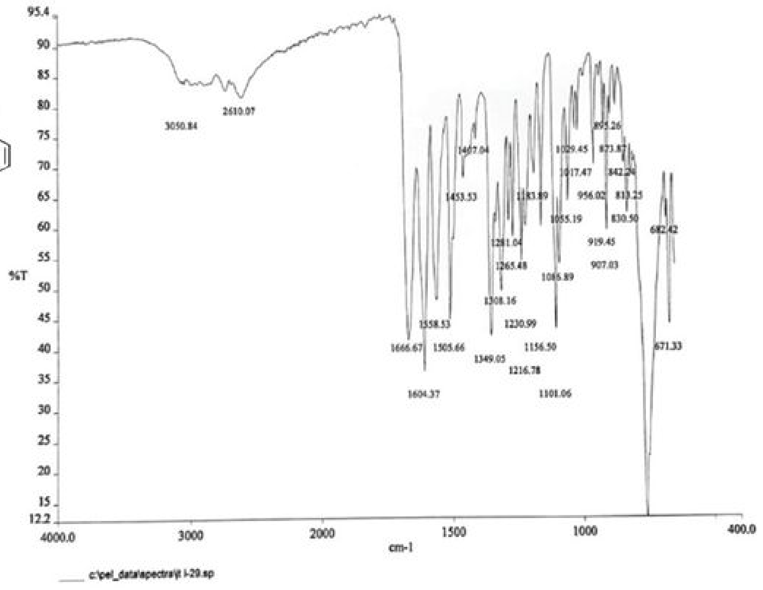

35

FigureS-35. IR spectrum of C-5.

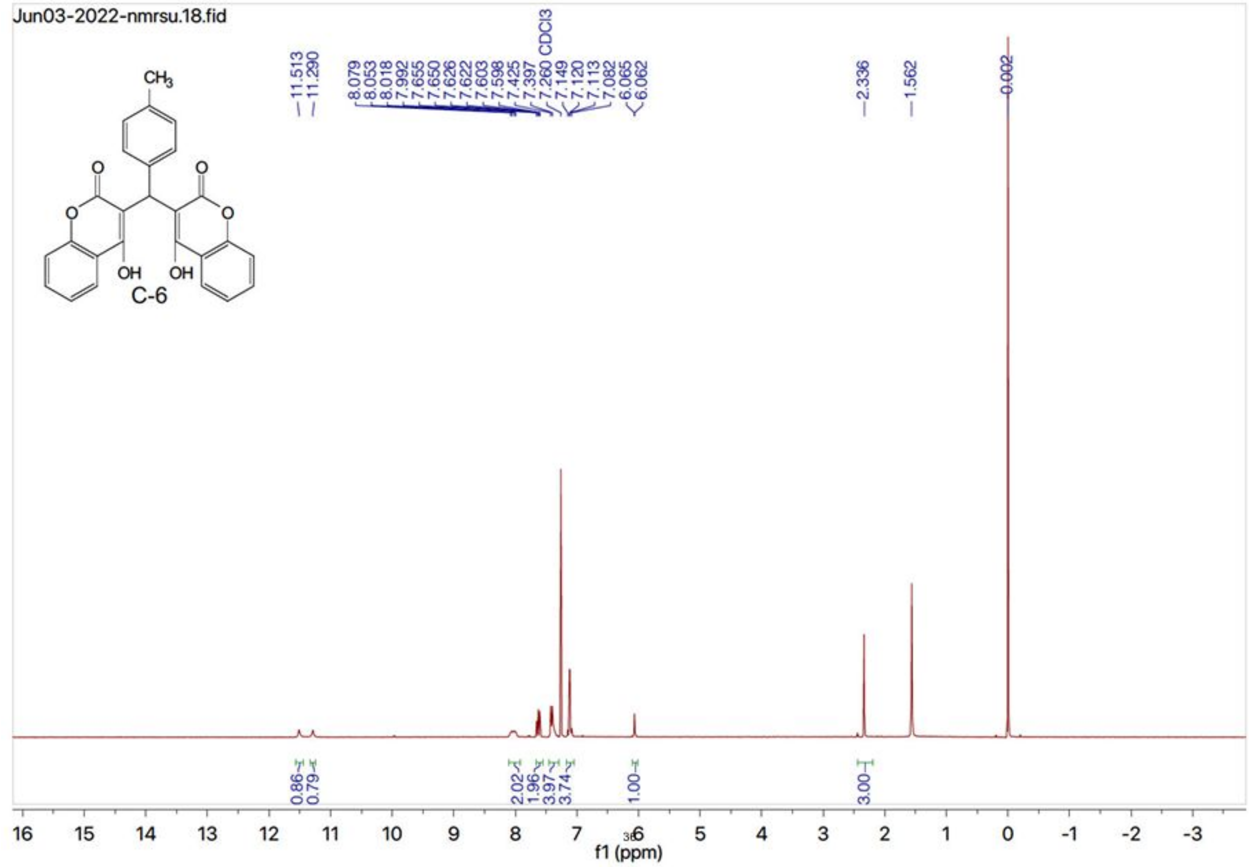

FigureS-36. ^{1}H NMR spectrum of C-6 in $CDCl_3$.

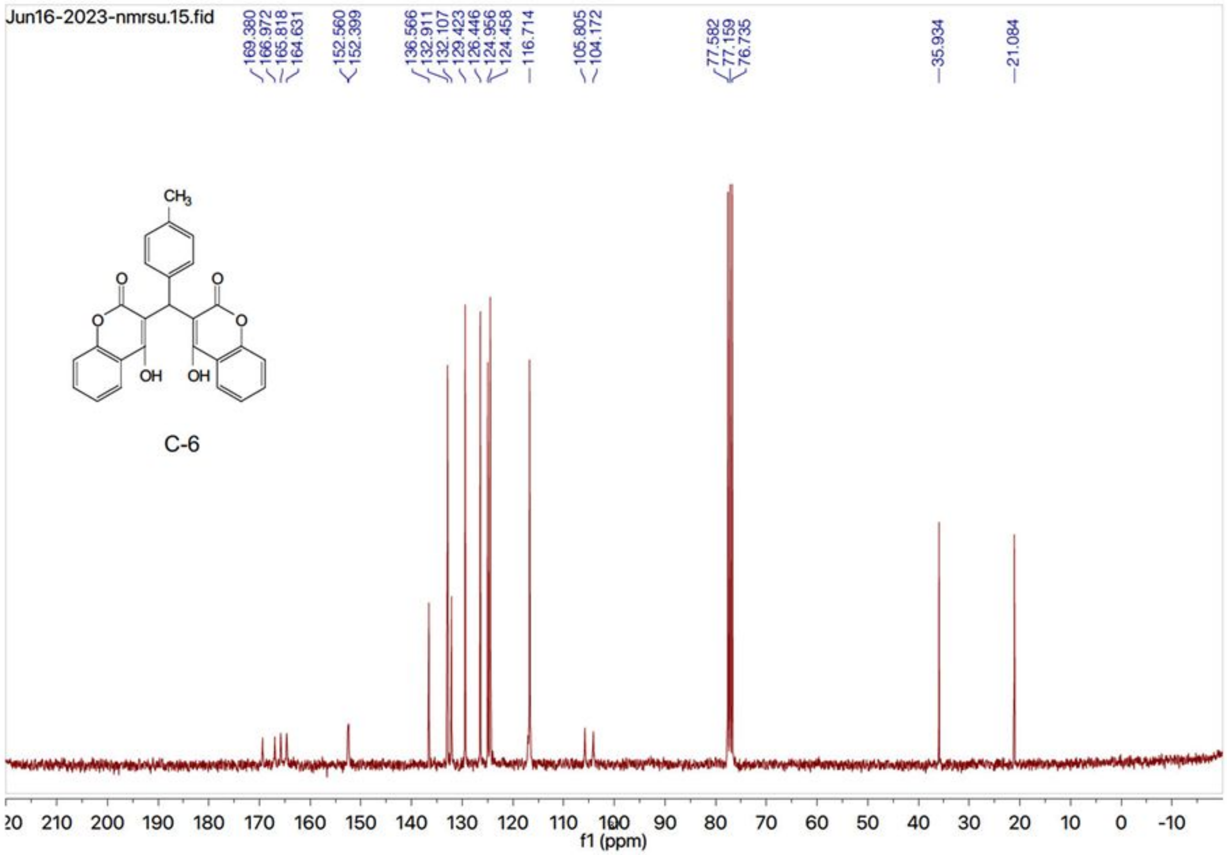

FigureS-37. ^{13}C NMR spectrum of C-6 in $CDCl_3$.

U of M

University of Minnesota Department of Chemistry
Mass Spectrometry Service Laboratory
email: chmmslab@umn.edu

Submit Sample To: Mass Spectrometry Facility
207 Pleasant St. SE
Minneapolis, MN 55455
Phone: (612)-625-8099
FAX : (612)-626-7541

Name: Julio Tapia	Phone: [redacted] Date: 6/12/2023
Email: tapiahjs@augsburg.edu Email Results? Y / N	FAX: FAX Results? Y / N
P.I./Advisor: Michael Wentzel	U of M Budget #
Company/University: Augsburg University	P.O.# (For non-U of M Clients) Augsburg University Chemistry Department
Shipping Address:	Billing Address: 2211 Riverside Ave, Minneapolis, MN 55454
Sample Label: C6	Molecular Weight: 426.42
Structural Formula or Sample Composition:	Molecular Formula: $C_{26}H_{18}O_6$
	Melting/Boiling Point:
	Solubility:
	Thermal Stability:
	Toxicity:
	Reactivity:

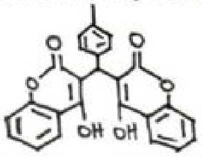

Chromatography Conditions:

Special Sample Considerations:

**Analysis Requested**	EI	CI	MALDI	ESI
Low Resolution Nominal Mass				
High Resolution Accurate Mass				✓
+Ve				
-Ve				
GCMS				
LCMS				

Instrument Used		Conditions Used
Finnigan MAT 95		Source Temp:
Bruker Reflex III		Acc. Voltage:
Bruker BioTOF II		Resolution:
Waters Triple Quad		Scan Range:
Waters Synapt G2		Gas Used:

Operator Comments
Neg mode, MeOH/DCM solvent
Na-TFA calibrant
M-1 theoretical 425.1031
observed 425.1047
error - 3.75 ppm

Log #:	Analyst:	Analysis Date:	Analyses Run:	Total Cost:

130190
C6
oesi
madeline honig
6/15/2023 2:44:14 PM

38

FigureS-38. HRMS results of C-6

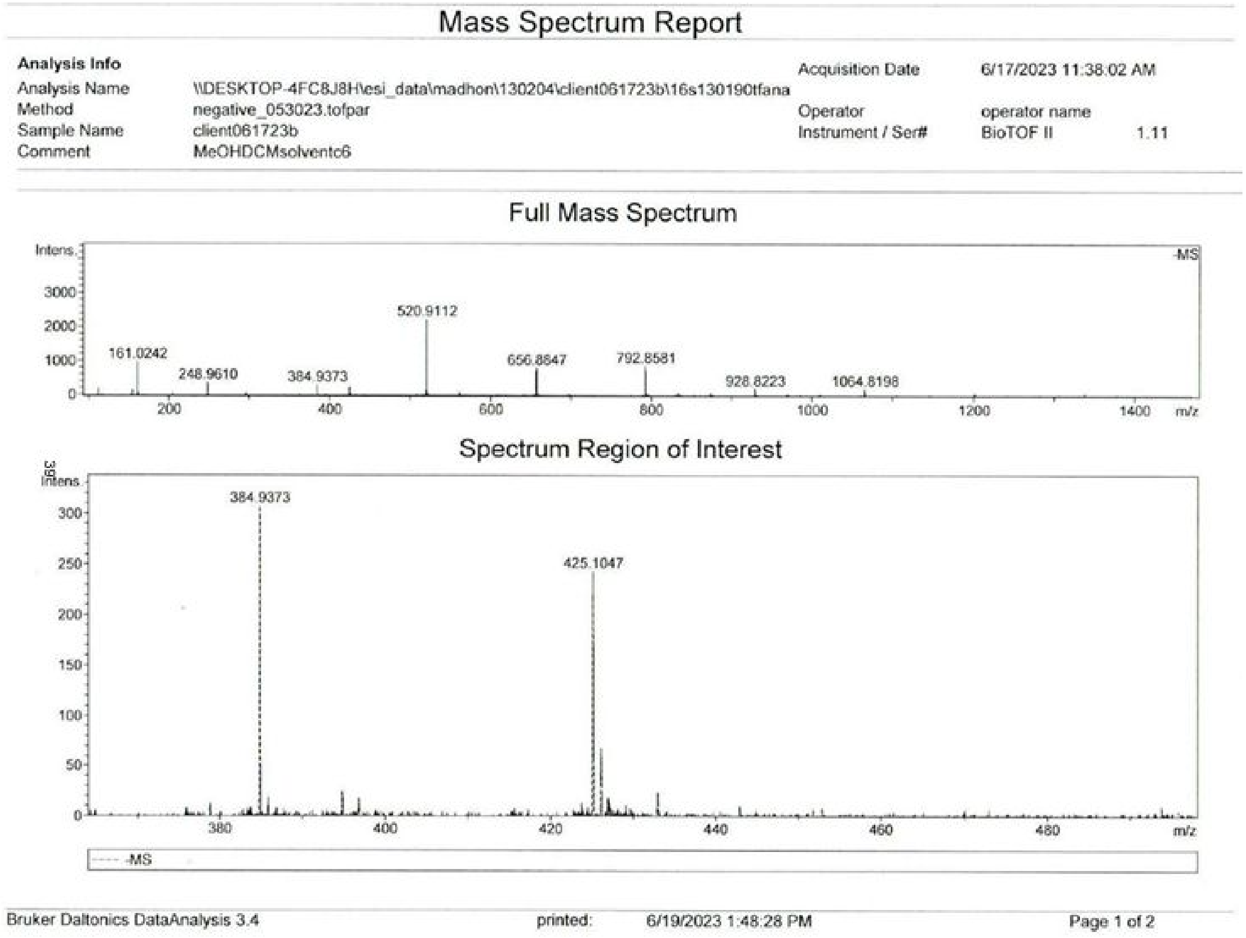

FigureS-39. HRMS results of C-6 spectrum.

Mass Spectrum Report

Elemental Composition Report

Generate Molecular Formula Parameter

Formula, min.	C0H17O6						
Formula, max.	C26H17O6						
Measured m/z	425.105	Tolerance	15	ppm		Charge	-1
Check Valence	no	Minimum	0			Maximum	0
Nirogen Rule	no	Electron Configuration		both			
Filter H/C Ratio	no	Minimum	0			Maximum	3
Estimate Carbon	yes						

Sum Formula	Sigma	m/z	Err [ppm]	Mean Err [ppm]	Err [mDa]	rdb	N Rule	e⁻
C 26 H 17 O 6	0.167	425.1031	-3.78	-3.75	-1.61	18.50	ok	even

Mass Spectrum Peak List

#	m/z	Area	Res.	S/N
1	112.9849	6	4583	9.7
2	154.9737	7	3612	7.9
3	161.0242	40	3992	50.9
4	248.9610	26	3629	45.4
5	384.9373	32	3932	24.2
6	425.1047	28	3032	16.2
7	520.9112	308	4006	132.9
8	521.9047	14	4056	6.4
9	561.0743	15	3668	8.4
10	656.8847	139	4008	53.4
11	792.8581	176	4156	59.2
12	928.8223	42	4343	17.1
13	1064.8198	40	4281	16.7

40

Bruker Daltonics DataAnalysis 3.4 printed: 6/19/2023 1:48:28 PM Page 2 of 2

FigureS-40. HRMS results of C-6 peak list.

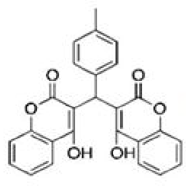

C-6

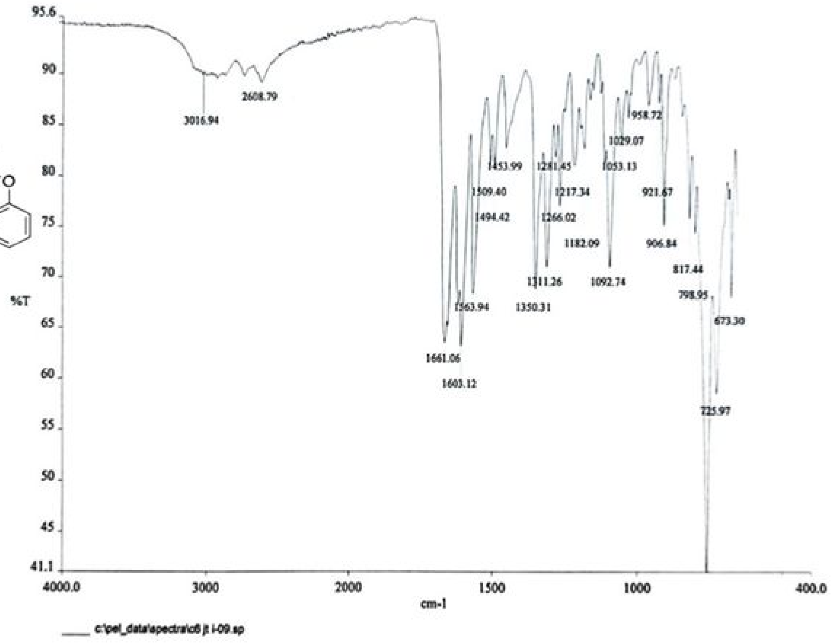

41

FigureS-41. IR spectrum of C-6.

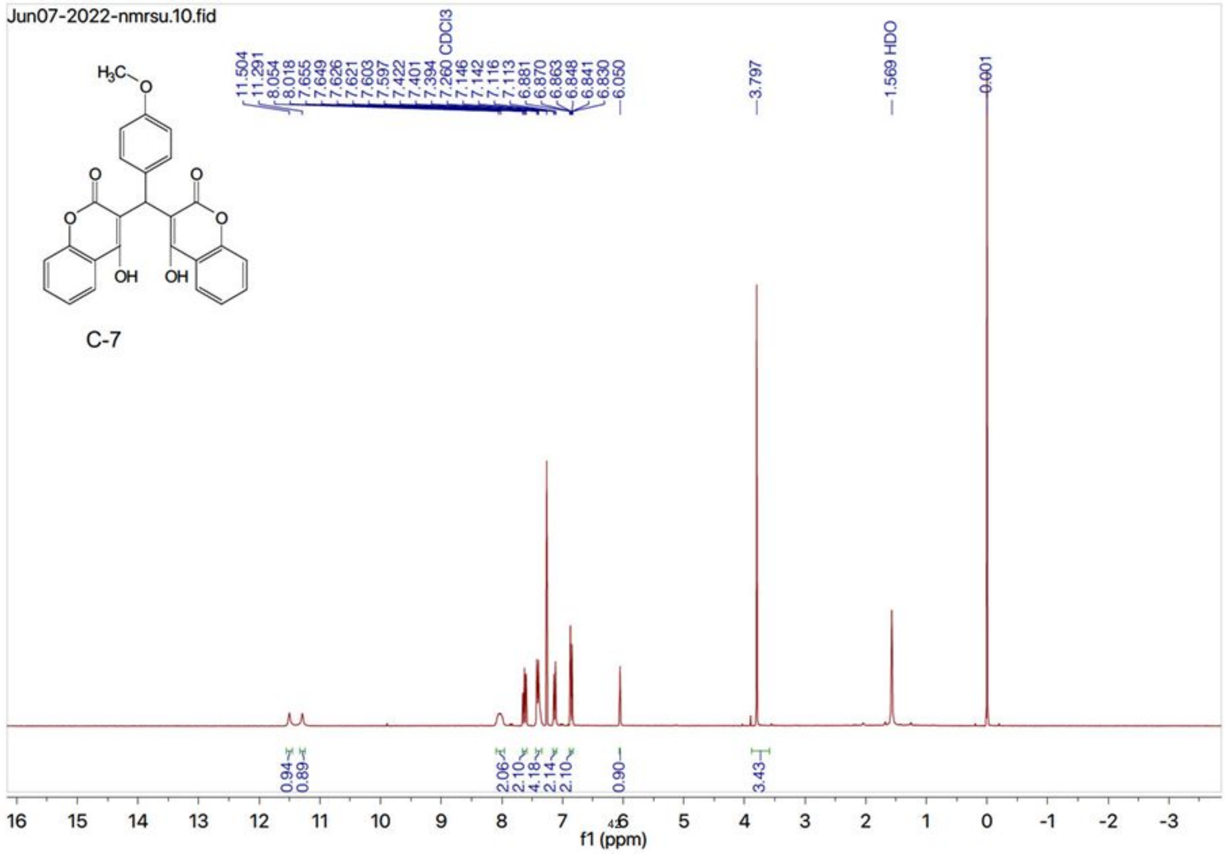

FigureS-42. ^{1}H NMR spectrum of C-7 in $CDCl_3$.

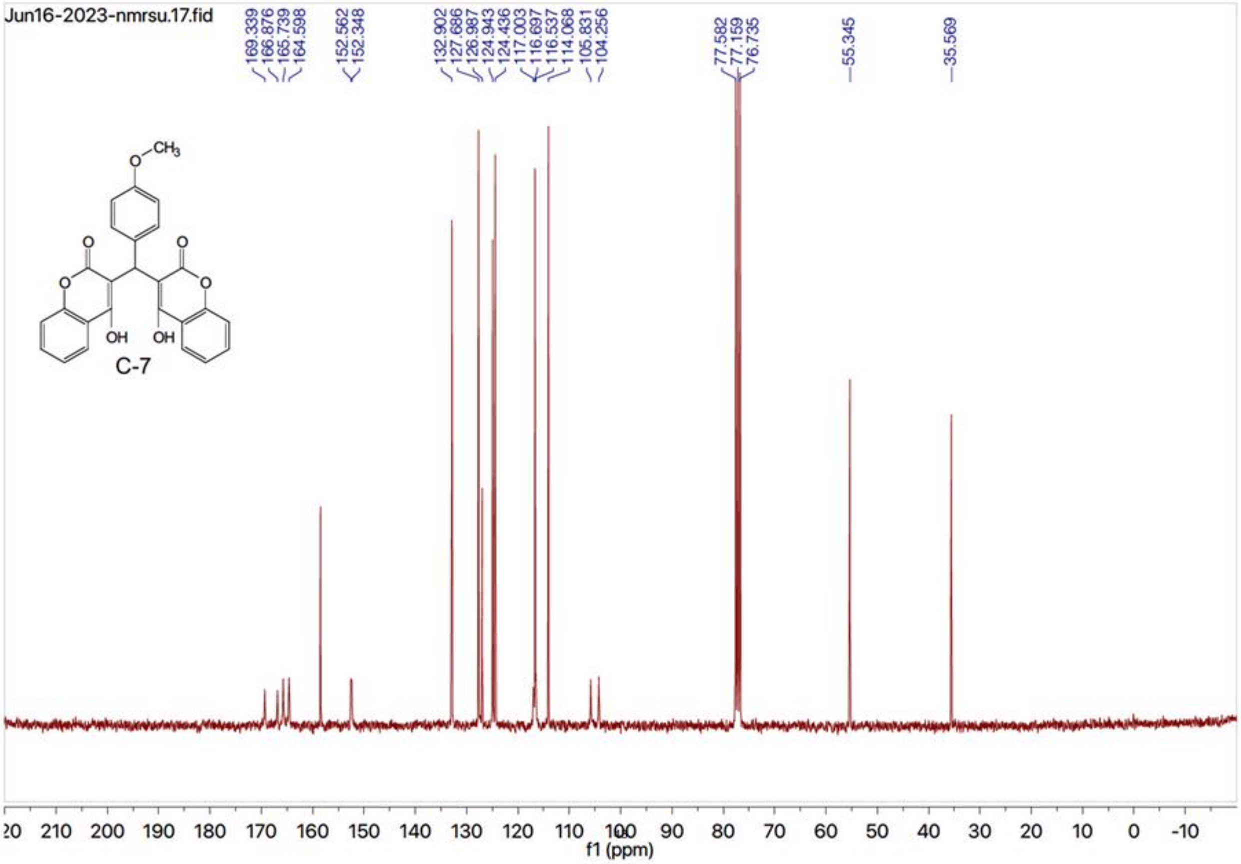

FigureS-43. ^{13}C NMR spectrum of C-7 in $CDCl_3$.

U of M
University of Minnesota Department of Chemistry
Mass Spectrometry Service Laboratory
email: chmmslab@umn.edu

Submit Sample To: Mass Spectrometry Facility
207 Pleasant St. SE
Minneapolis, MN 55455
Phone: (612)-625-8099
FAX: (612)-626-7541

Name: Julio Tapia	Phone: [redacted] Date: 6/12/2023
Email: tapiahs5 @ augsburg.edu Email Results? (Y) / N	FAX: FAX Results? Y / N
P.I./Advisor: Michael Wentzel	U of M Budget #
Company/University: Augsburg University	P.O.# (For non-U of M Clients) Augsburg University Chemistry Department
Shipping Address:	Billing Address: 2211 Riverside Ave, Minneapolis, MN 55454
Sample Label: C7	Molecular Weight: 442.42
Structural Formula or Sample Composition:	Molecular Formula: $C_{26}H_{18}O_7$
	Melting/Boiling Point:
	Solubility:
	Thermal Stability:
	Toxicity:
	Reactivity:

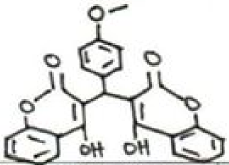

Chromatography Conditions:

Special Sample Considerations:

Analysis Requested

	EI	CI	MALDI	ESI
Low Resolution Nominal Mass				
High Resolution Accurate Mass				√
+Ve				
-Ve				
GCMS				
LCMS				

Instrument Used		Conditions Used	Operator Comments
Finnigan MAT 95		Source Temp:	NEG mode. MeOH/DCM solvent
Bruker Reflex III		Acc. Voltage:	TFA-Na calibrant
Bruker BioTOF II		Resolution:	M-1 theoretical 441.0980
Waters Triple Quad		Scan Range:	observed 441.0993
Waters Synapt G2		Gas Used:	error -3.10 ppm

Log #:	Analyst:	Analysis Date:	Analyses Run:	Total Cost:

130191
C7
oesi
madeline honig
6/15/2023 2:45:24 PM

44

FigureS-44. HRMS results of C-7

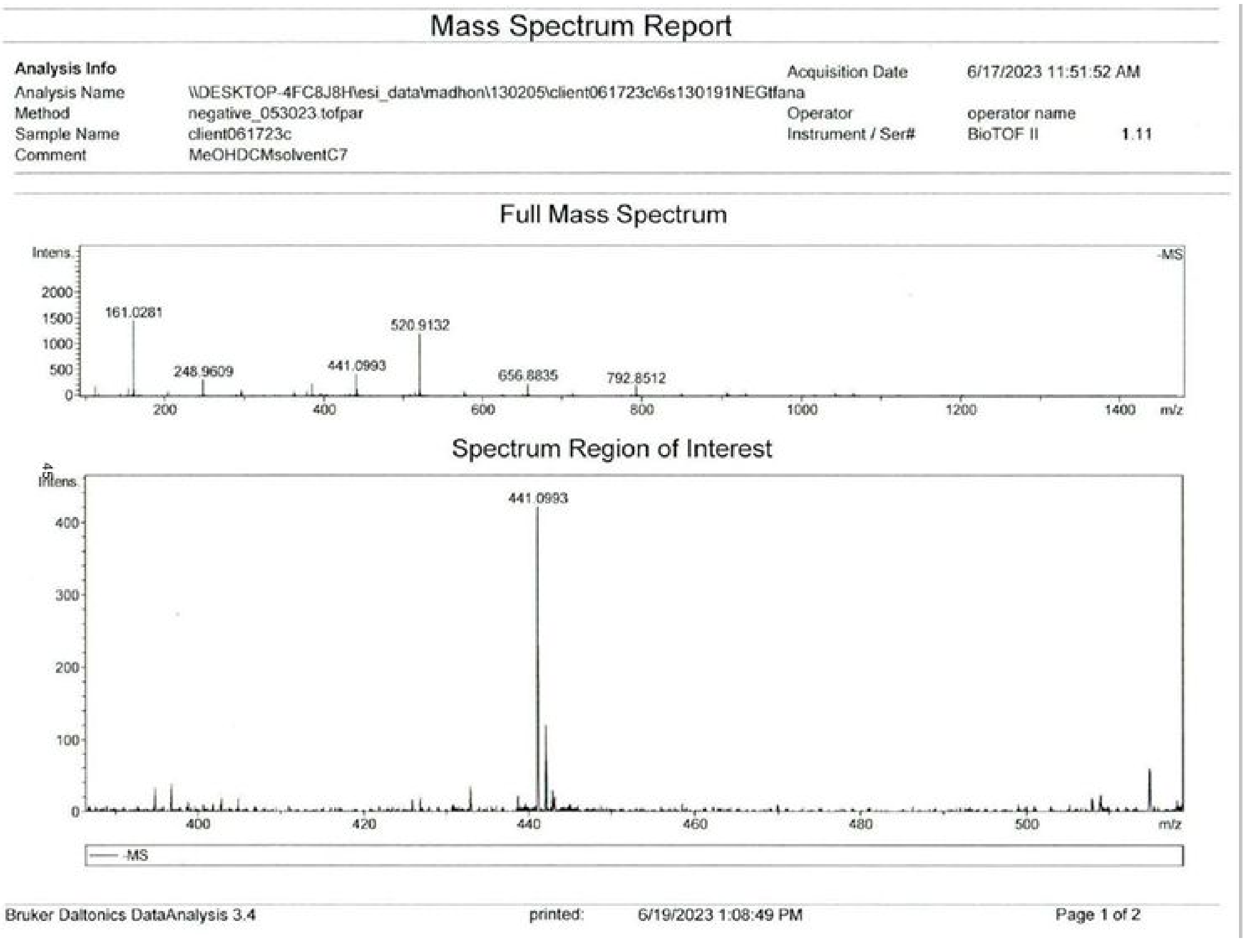

FigureS-45. HRMS results of C-7 spectrum.

Mass Spectrum Report

Elemental Composition Report

Generate Molecular Formula Parameter

Formula, min.	C20H17O7						
Formula, max.	C26H17O7						
Measured m/z	441.099	Tolerance	20	ppm		Charge	-1
Check Valence	no	Minimum	0			Maximum	0
Nirogen Rule	no	Electron Configuration		both			
Filter H/C Ratio	no	Minimum	0			Maximum	3
Estimate Carbon	yes						

Sum Formula	Sigma	m/z	Err [ppm]	Mean Err [ppm]	Err [mDa]	rdb	N Rule	e^-
C 26 H 17 O 7	0.031	441.0980	-3.10	-2.45	-1.37	18.50	ok	even

Mass Spectrum Peak List

46

#	m/z	Area	Res.	S/N
1	112.9897	5	4525	7.8
2	154.9792	5	5462	7.3
3	161.0281	56	4378	73.0
4	162.0338	4	5568	5.7
5	248.9609	20	4091	31.7
6	384.9329	23	3889	18.1
7	441.0993	47	3911	29.8
8	442.1015	12	4274	8.5
9	520.9132	162	4236	92.6
10	656.8835	42	3321	23.2
11	792.8512	41	3019	15.9

Bruker Daltonics DataAnalysis 3.4 printed: 6/19/2023 1:08:49 PM Page 2 of 2

FigureS-46. HRMS results of C-7 peak list.

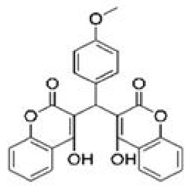

C-7

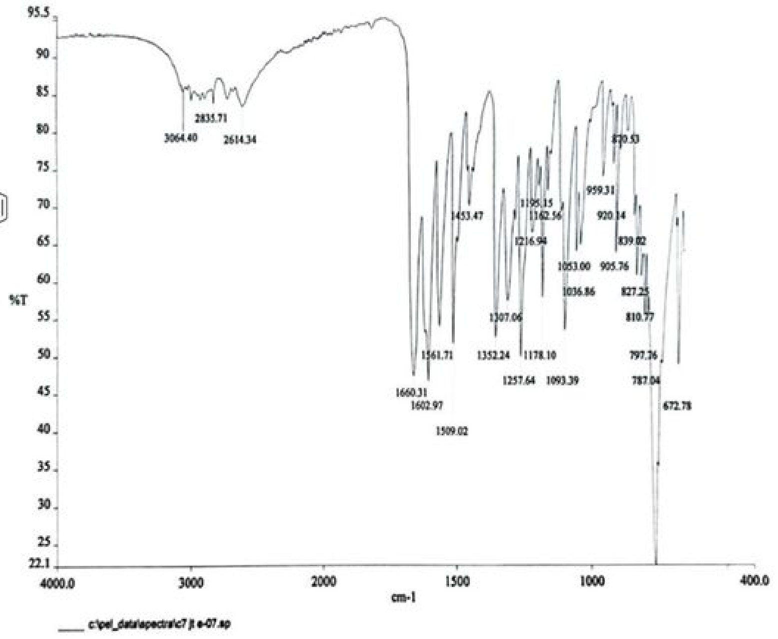

47

FigureS-47. IR spectrum of C-7.

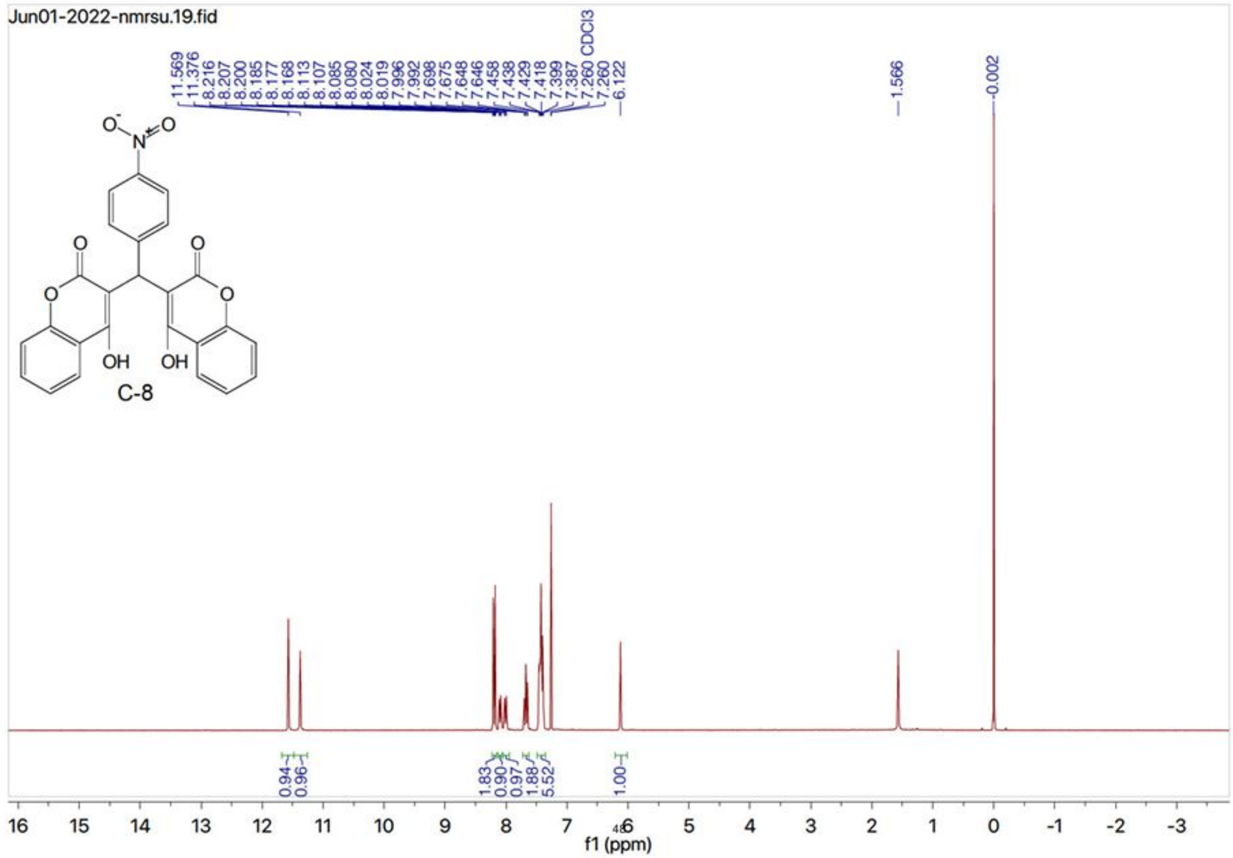

FigureS-48. ^{1}H NMR spectrum of C-8 in $CDCl_3$.

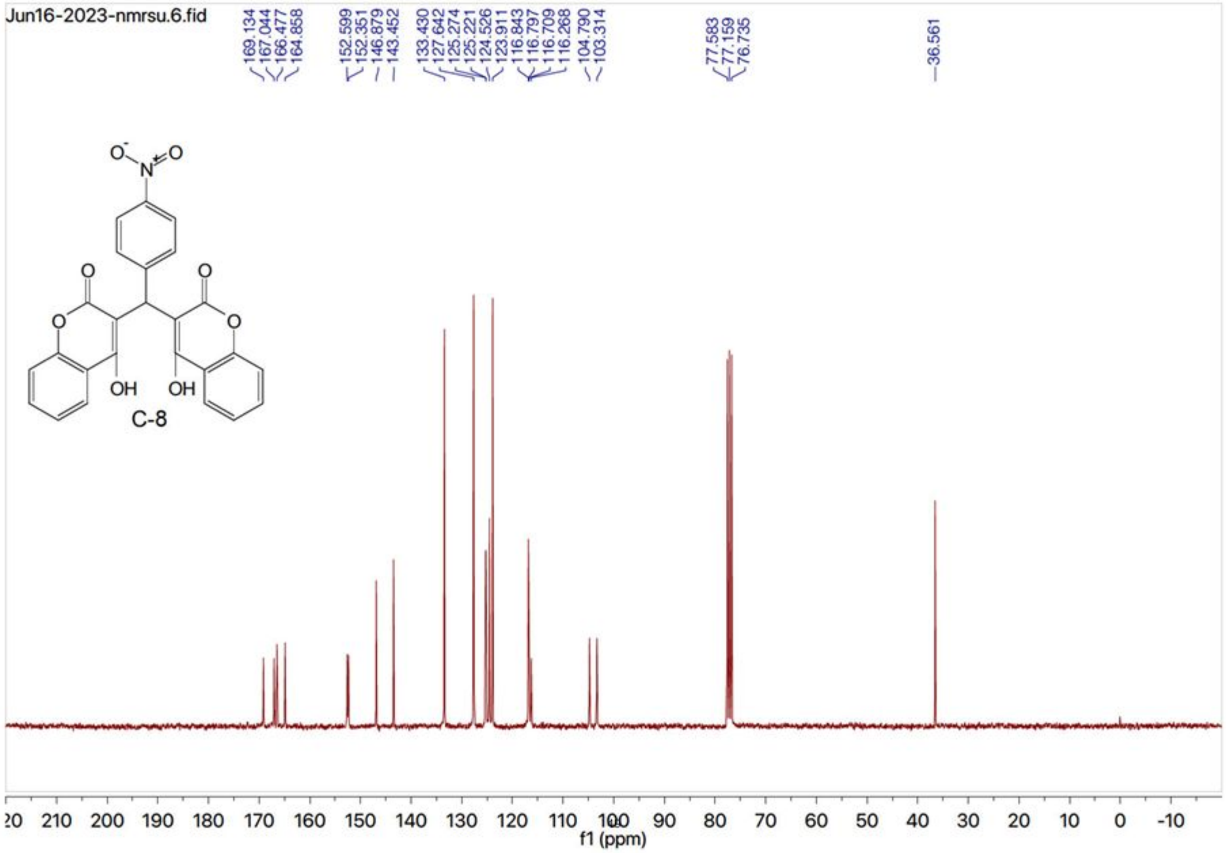

FigureS-49. ^{13}C NMR spectrum of C-8 in $CDCl_3$.

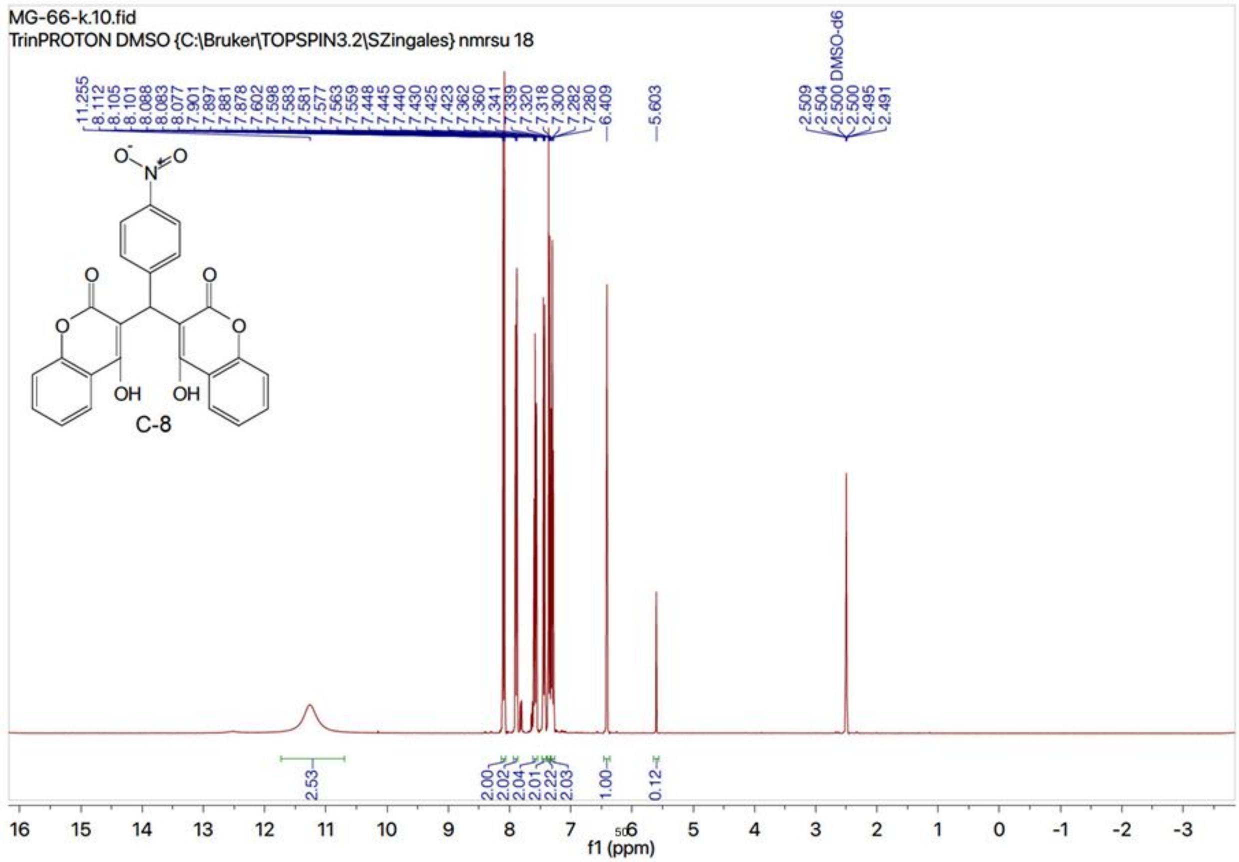

FigureS-50. DMSO-d_6 1H NMR

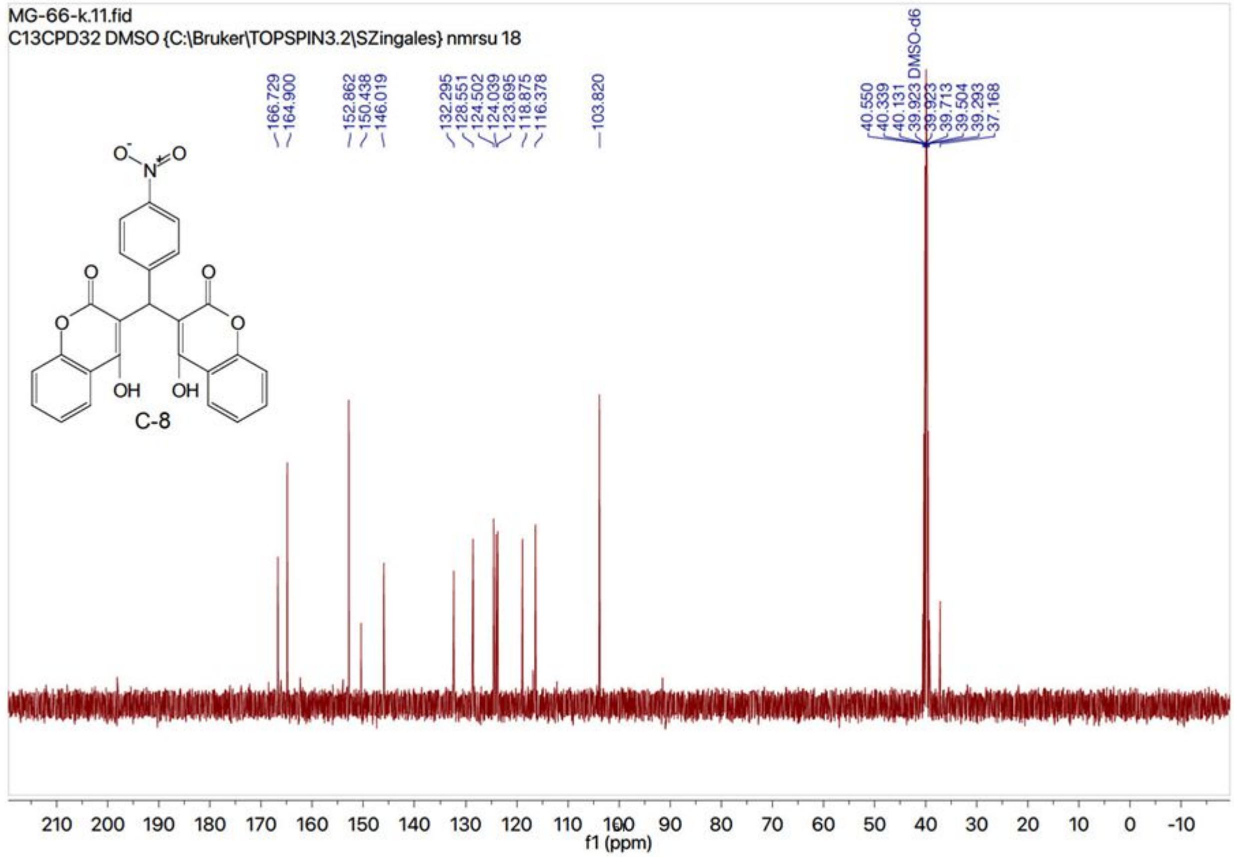

FigureS-51. DMSO-d_6 ^{13}C NMR spectra of C-8

U of M

University of Minnesota Department of Chemistry
Mass Spectrometry Service Laboratory
email: chmmslab@umn.edu

Submit Sample To: Mass Spectrometry Facility
207 Pleasant St. SE
Minneapolis, MN 55455
Phone: (612)-625-8099
FAX : (612)-626-7541

Name: Julio Tapia	Phone: [redacted] Date: 6/12/2023
Email: tapiahjs@augsburg.edu Email Results? Y/N	FAX: FAX Results? Y / N
P.I./Advisor: Michael Wentzel	U of M Budget #
Company/University: Augsburg University	P.O.# (For non-U of M Clients) Augsburg University Chemistry Department
Shipping Address:	Billing Address: 2211 Riverside Ave, Minneapolis, MN 55454
Sample Label: C8	Molecular Weight: 457.39
Structural Formula or Sample Composition:	Molecular Formula: $C_{25}H_{15}NO_8$
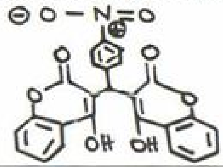	Melting/Boiling Point:
	Solubility:
	Thermal Stability:
	Toxicity:
	Reactivity:

Chromatography Conditions:

Special Sample Considerations:

Analysis Requested	EI	CI	MALDI	ESI
Low Resolution Nominal Mass				
High Resolution Accurate Mass				✓
+Ve				
-Ve				
GCMS				
LCMS				

Instrument Used		Conditions Used
Finnigan MAT 95		Source Temp:
Bruker Reflex III		Acc. Voltage:
Bruker BioTOF II		Resolution:
Waters Triple Quad		Scan Range:
Waters Synapt G2		Gas Used:

Operator Comments

MeOH/DCM solvent
Neg mode. TFA-Na calibrant
M-1 theoretical 456.0725
observed 456.0730
error -1.10 ppm

Log #:	Analyst:	Analysis Date:	Analyses Run:	Total Cost:

130186
C1
oesi
madeline honig
6/15/2023 2:37:21 PM

52

FigureS-52. HRMS results of C-8

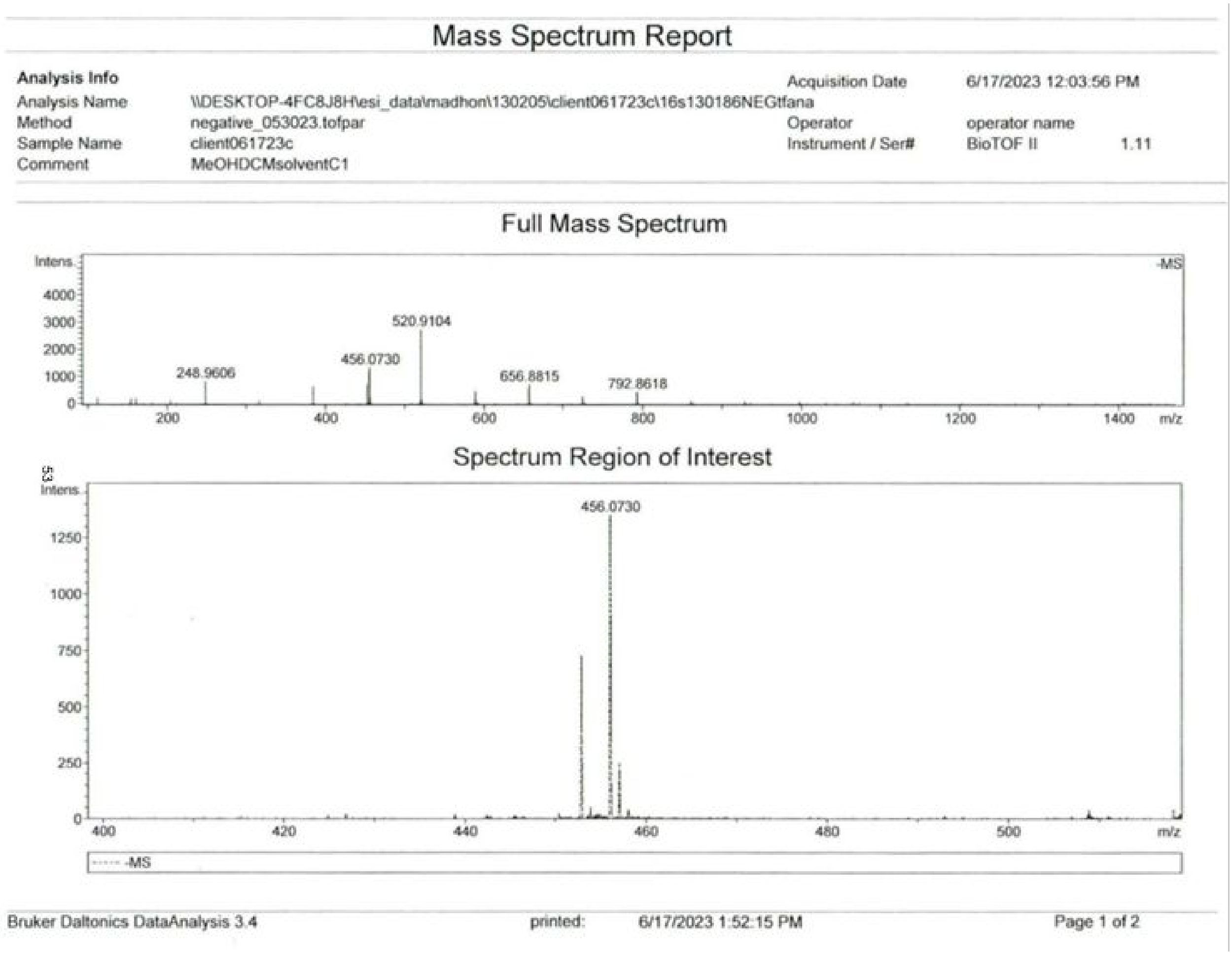

FigureS-53. HRMS results of C-8 spectrum.

Mass Spectrum Report

Elemental Composition Report

Generate Molecular Formula Parameter

Formula, min.	C10H14N1O8						
Formula, max.	C25H15NO8						
Measured m/z	456.073	Tolerance	15	ppm		Charge	-1
Check Valence	no	Minimum	0			Maximum	0
Nirogen Rule	no	Electron Configuration		both			
Filter H/C Ratio	no	Minimum	0			Maximum	3
Estimate Carbon	yes						

Sum Formula	Sigma	m/z	Err [ppm]	Mean Err [ppm]	Err [mDa]	rdb	N Rule	e⁻
C 25 H 14 N 1 O 8	0.068	456.0725	-1.10	1.32	-0.50	19.50	ok	even

Mass Spectrum Peak List

54

#	m/z	Area	Res.	S/N
1	112.9854	6	4636	15.2
2	154.9748	6	5382	15.0
3	161.0248	6	5162	15.0
4	204.9697	5	4894	8.8
5	248.9606	48	4696	69.2
6	316.9412	8	4888	12.7
7	384.9360	64	4166	38.7
8	452.9200	80	4636	38.5
9	456.0730	159	3910	72.0
10	457.0699	30	3229	12.6
11	520.9104	348	4463	179.8
12	521.9179	16	4223	7.7
13	588.8970	66	4588	41.4
14	656.8815	119	3529	51.3
15	724.8790	53	3992	18.0
16	792.8618	90	4322	38.8
17	860.8455	27	3948	12.0

Bruker Daltonics DataAnalysis 3.4 printed: 6/17/2023 1:52:15 PM Page 2 of 2

FigureS-54. HRMS results of C-8 peak list.

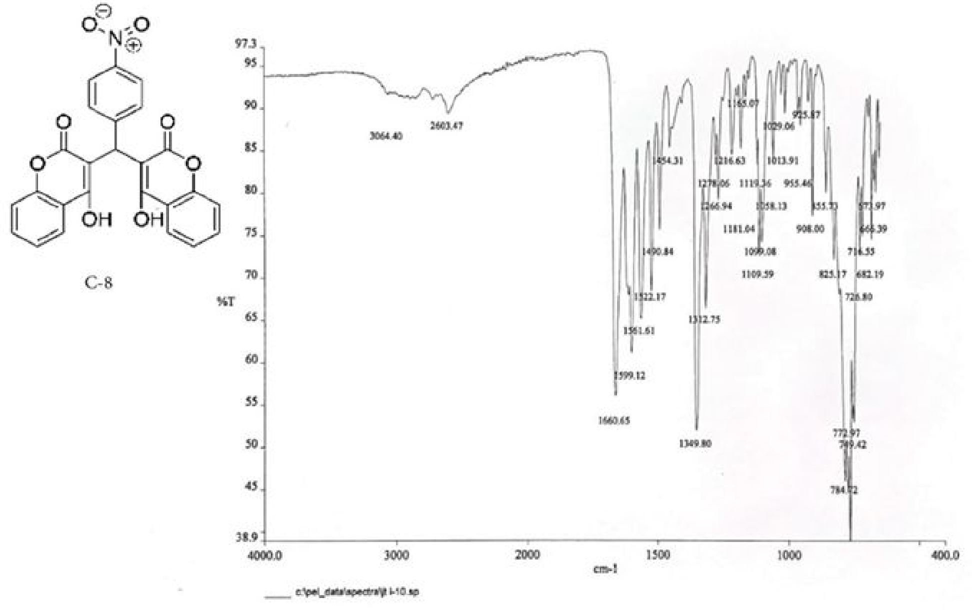

55

FigureS-55. IR spectrum of C-8.

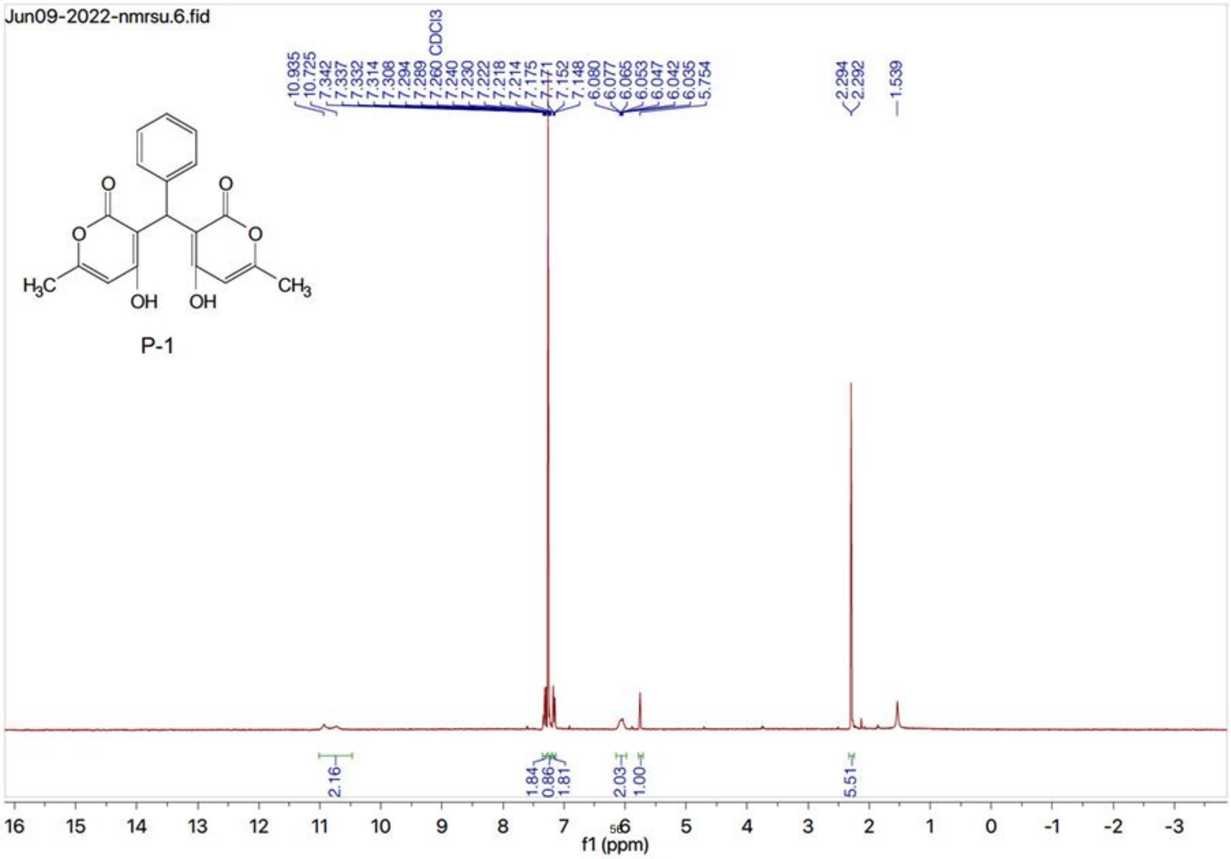

FigureS-56. ^{1}H NMR spectrum of P-1 in $CDCl_3$.

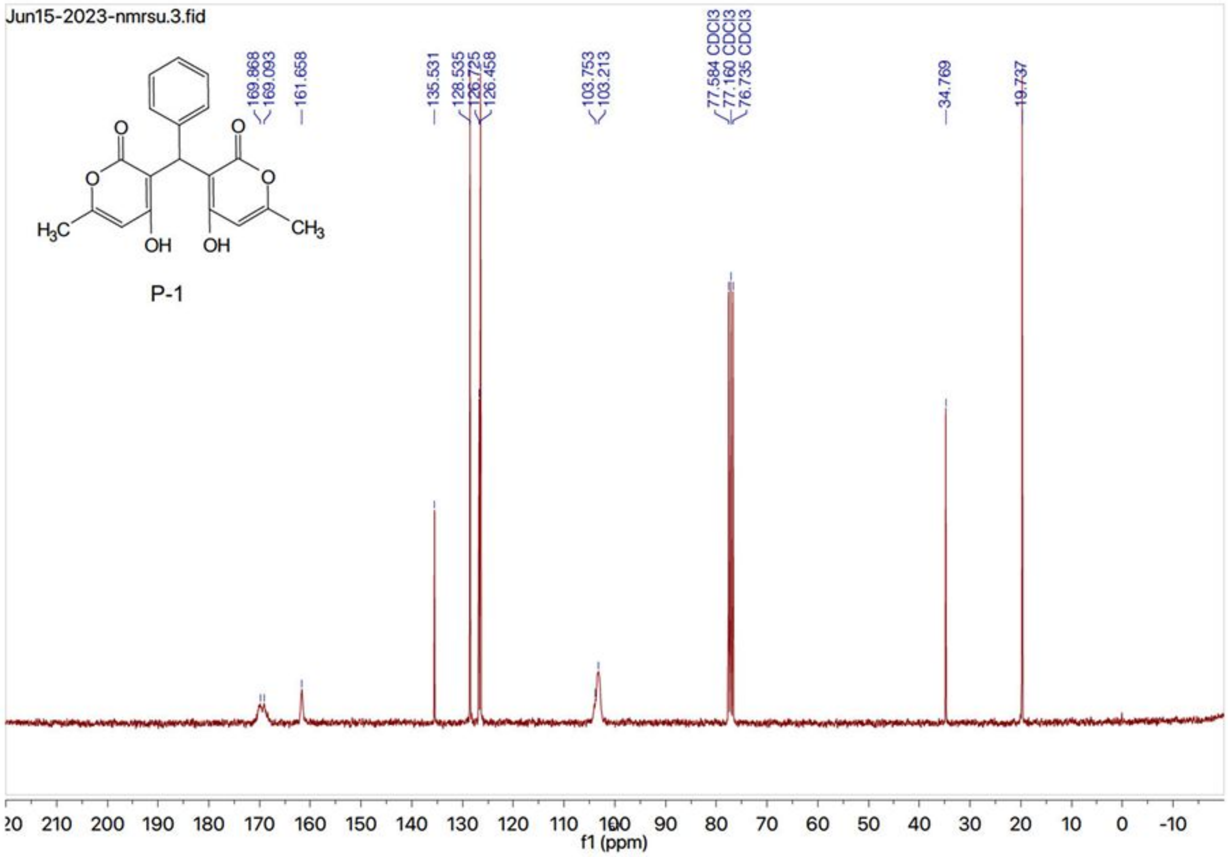

FigureS-57. ^{13}C NMR spectrum of P-1 in $CDCl_3$.

username, acct# [redacted]

U of M

University of Minnesota Department of Chemistry
Mass Spectrometry Service Laboratory
email: chmmslab@umn.edu

Submit Sample To: Mass Spectrometry Facility
207 Pleasant St. SE
Minneapolis, MN 55455
Phone: (612)-625-8099
FAX : (612)-626-7541

Name: Julio Tapia	Phone: [redacted] Date: 6/12/2023
Email: tapiahs@augsburg.edu Email Results? Y / N	FAX: FAX Results? Y / N
P.I./Advisor: Michael Wentzel	U of M Budget #
Company/University: Augsburg University	P.O.# (For non-U of M Clients) Augsburg University Chemistry Department
Shipping Address:	Billing Address: 2211 Riverside Ave, Minneapolis, MN 55454
Sample Label: P1	Molecular Weight: 340.33
Structural Formula or Sample Composition:	Molecular Formula: $C_{19}H_{16}O_6$
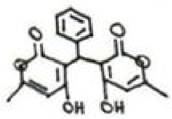	Melting/Boiling Point:
	Solubility:
	Thermal Stability:
	Toxicity:
	Reactivity:

Chromatography Conditions:

Special Sample Considerations:

Analysis Requested	EI	CI	MALDI	ESI
Low Resolution Nominal Mass				
High Resolution Accurate Mass				√
+Ve				
-Ve				
GCMS				
LCMS				

Instrument Used	Conditions Used	Operator Comments
Finnigan MAT 95	Source Temp:	POS mode, MeOH solvent
Bruker Reflex III	Acc. Voltage:	PEG300 Calibrant
Bruker BioTOF II	Resolution:	M+Na theoretical: 363.0839
Waters Triple Quad	Scan Range:	obs: 363.0835
Waters Synapt G2	Gas Used:	err: 1.07 ppm

Log #:	Analyst:	Analysis Date:	Analyses Run:	Total Cost:

130179
P1
oesi
madeline honig
6/15/2023 2:26:23 PM

58

FigureS-58. HRMS results of P-1

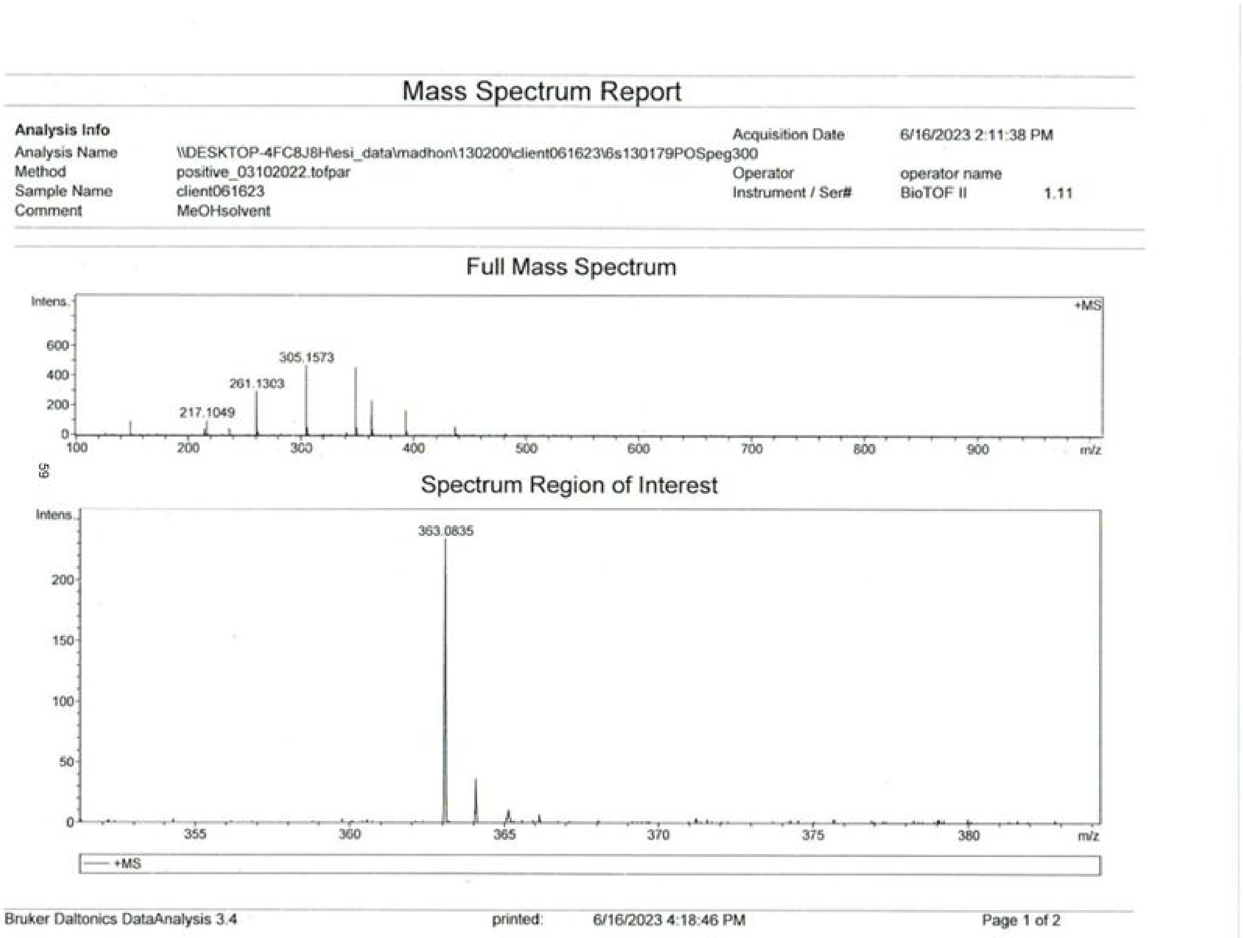

FigureS-59. HRMS results of P-1 spectrum.

Mass Spectrum Report

Elemental Composition Report

Generate Molecular Formula Parameter

Formula, min.	C0H16O6						
Formula, max.	C19H16O6Na						
Measured m/z	363.084	Tolerance	10	ppm		Charge	1
Check Valence	no	Minimum	0			Maximum	0
Nirogen Rule	no	Electron Configuration		both			
Filter H/C Ratio	no	Minimum	0			Maximum	3
Estimate Carbon	yes						

Sum Formula	Sigma	m/z	Err [ppm]	Mean Err [ppm]	Err [mDa]	rdb	N Rule	e⁻
C 19 H 16 Na 1 O 6	0.123	363.0839	1.07	1.06	0.39	11.50	ok	even

Mass Spectrum Peak List

#	m/z	Area	Res.	S/N
1	217.1049	3	7974	5.5
2	261.1303	11	6928	12.9
3	305.1573	19	7813	23.0
4	349.1837	17	11127	18.4
5	363.0835	9	10756	8.8
6	393.2092	8	8751	7.7

60

Bruker Daltonics DataAnalysis 3.4 printed: 6/16/2023 4:18:46 PM Page 2 of 2

FigureS-60. HRMS results of P-1 peak list.

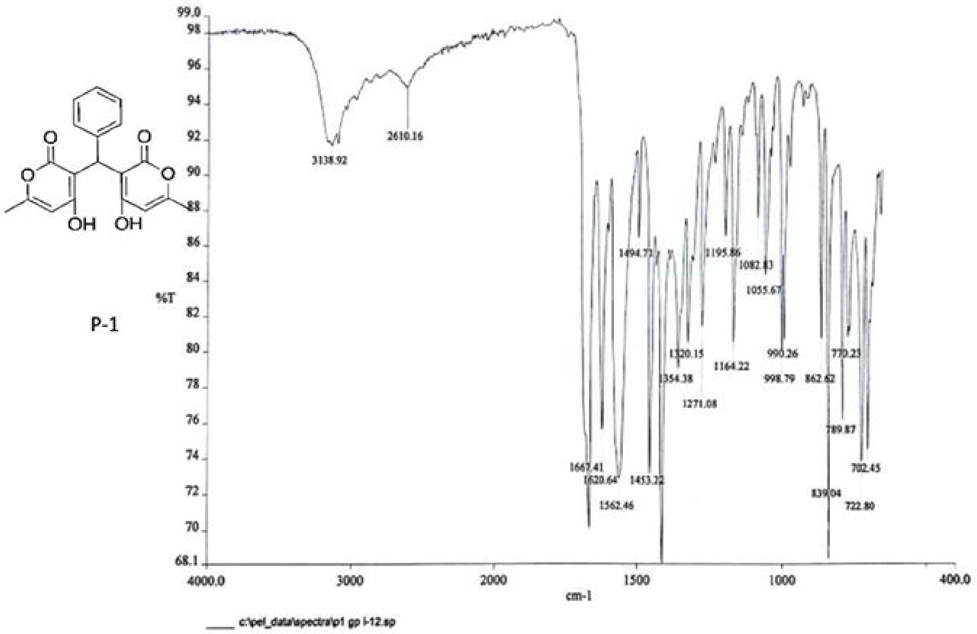

61

FigureS-61. IR spectrum of P-1.

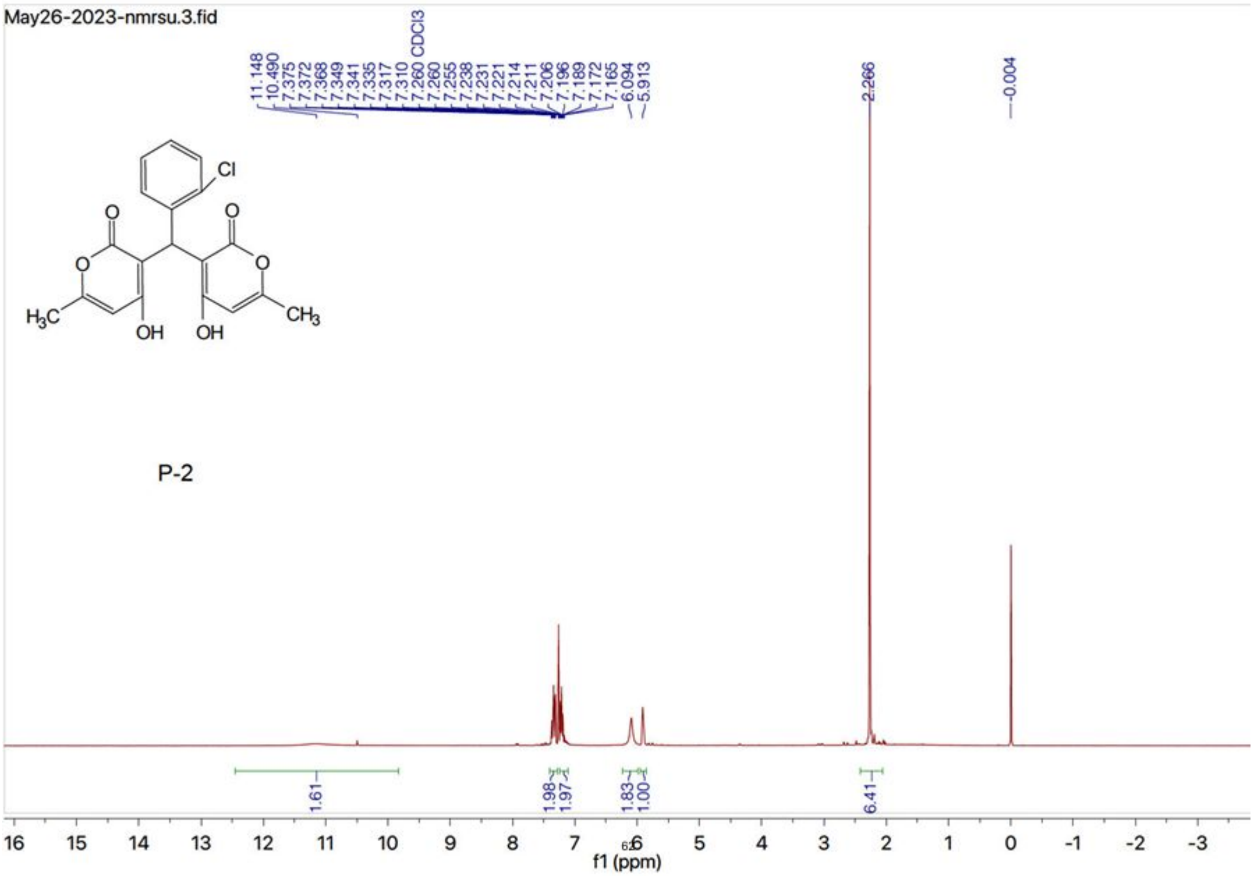

FigureS-62. ^1H NMR spectrum of P-2 in $CDCl_3$.

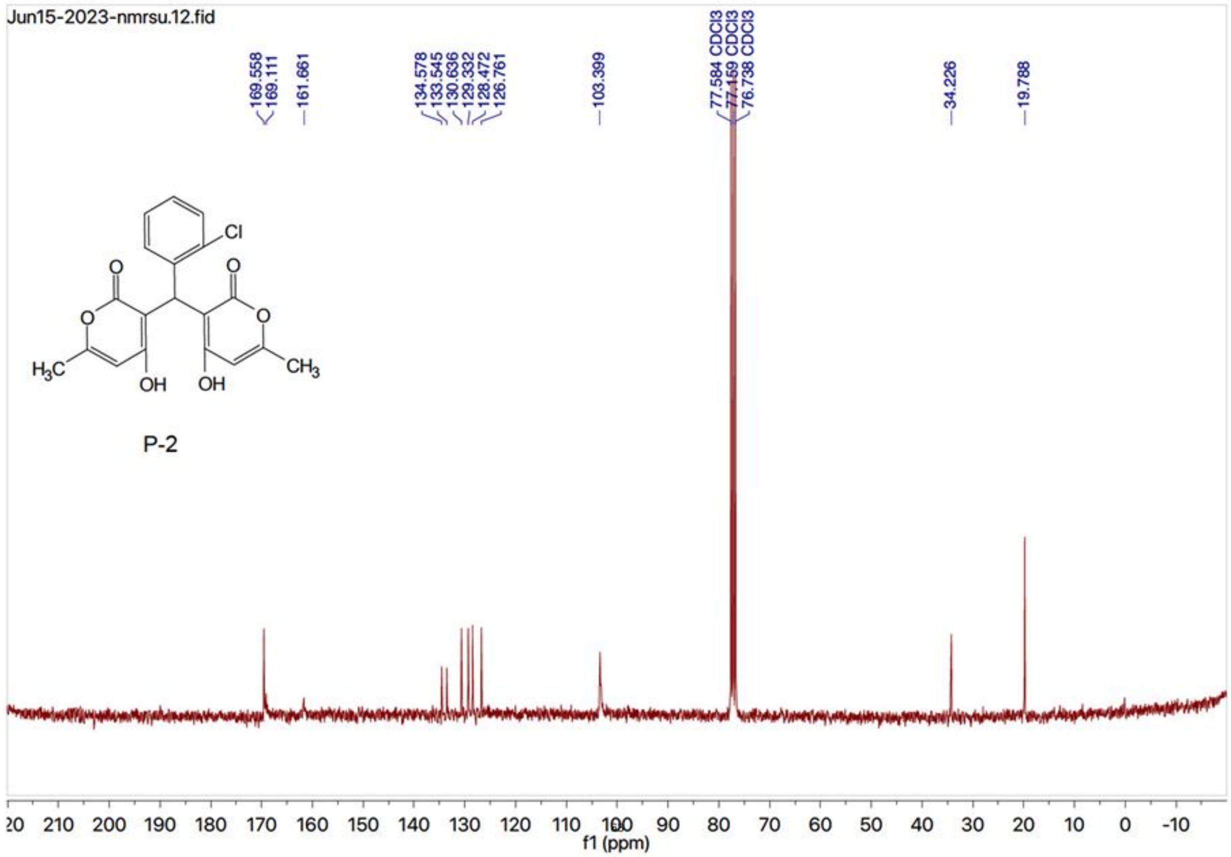

FigureS-63. ^{13}C NMR spectrum of P-2 in $CDCl_3$.

U of M

University of Minnesota Department of Chemistry
Mass Spectrometry Service Laboratory
email: chmmslab@umn.edu

Submit Sample To: Mass Spectrometry Facility
207 Pleasant St. SE
Minneapolis, MN 55455
Phone: (612)-625-8099
FAX : (612)-626-7541

Name: Julio Tapia	Phone: [redacted] Date: 6/12/2023
Email: tapiahjs @ augsburg.edu Email Results? Y / N	FAX: [redacted] FAX Results? Y / N
P.I./Advisor: Michael Wentzel	U of M Budget #
Company/University: Augsburg university	P.O.# (For non-U of M Clients) Augsburg university Chemistry Department
Shipping Address:	Billing Address: 2211 Riverside Ave, Minneapolis, MN 55454
Sample Label: P2	Molecular Weight: 374.77
Structural Formula or Sample Composition:	Molecular Formula: $C_{19}H_{15}ClO_6$
	Melting/Boiling Point:
	Solubility:
	Thermal Stability:
	Toxicity:
	Reactivity:

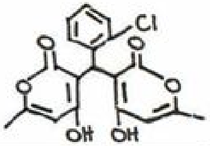

Chromatography Conditions:

Special Sample Considerations:

Analysis Requested

	EI	CI	MALDI	ESI
Low Resolution Nominal Mass				
High Resolution Accurate Mass				√
+Ve				
-Ve				
GCMS				
LCMS				

Instrument Used		Conditions Used
Finnigan MAT 95		Source Temp:
Bruker Reflex III		Acc. Voltage:
Bruker BioTOF II		Resolution:
Waters Triple Quad		Scan Range:
Waters Synapt G2		Gas Used:

Operator Comments

pos mode, MeOH solvent
PEG400 calibrant
M+Na+ theoretical 397.0449
observed 397.0449
error -0.01 ppm

Log #:	Analyst:	Analysis Date:	Analyses Run:	Total Cost:

130184
P6
oesi
madeline honig
6/15/2023 2:34:55 PM

64

FigureS-64. HRMS results of P-2

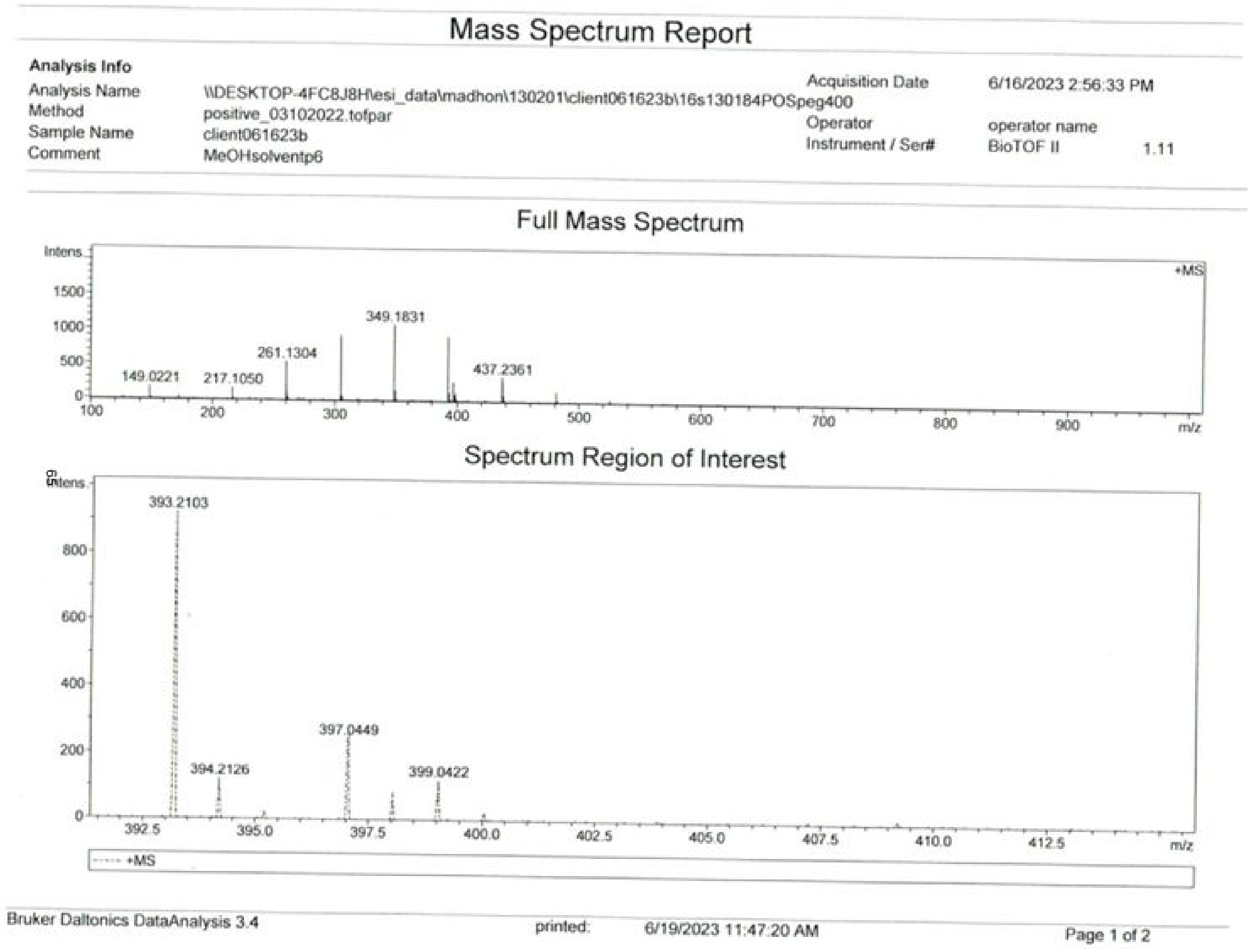

FigureS-65. HRMS results of P-2 spectrum.

Mass Spectrum Report

Elemental Composition Report

Generate Molecular Formula Parameter

Formula, min.	C0H15Cl1O6						
Formula, max.	C19H15ClO6Na						
Measured m/z	397.045	Tolerance	10	ppm		Charge	1
Check Valence	no	Minimum	0			Maximum	0
Nirogen Rule	no	Electron Configuration		both			
Filter H/C Ratio	no	Minimum	0			Maximum	3
Estimate Carbon	yes						

Sum Formula	Sigma	m/z	Err [ppm]	Mean Err [ppm]	Err [mDa]	rdb	N Rule	e⁻
C 19 H 15 Cl 1 Na 1 O 6	0.117	397.0449	-0.01	0.48	-0.00	11.50	ok	even

Mass Spectrum Peak List

#	m/z	Area	Res.	S/N
1	149.0221	4	6702	10.1
2	217.1050	5	8221	8.9
3	261.1304	16	10381	21.7
4	305.1568	32	10392	44.3
5	349.1831	46	8776	32.5
6	350.1865	5	11524	4.3
7	393.2103	42	9140	28.6
8	394.2126	5	12197	3.8
9	397.0449	14	7726	8.1
10	399.0422	5	17698	4.1
11	437.2361	18	8270	25.9
12	481.2614	7	6418	14.7

Bruker Daltonics DataAnalysis 3.4 printed: 6/19/2023 11:47:20 AM Page 2 of 2

FigureS-66. HRMS results of P-2 peak list.

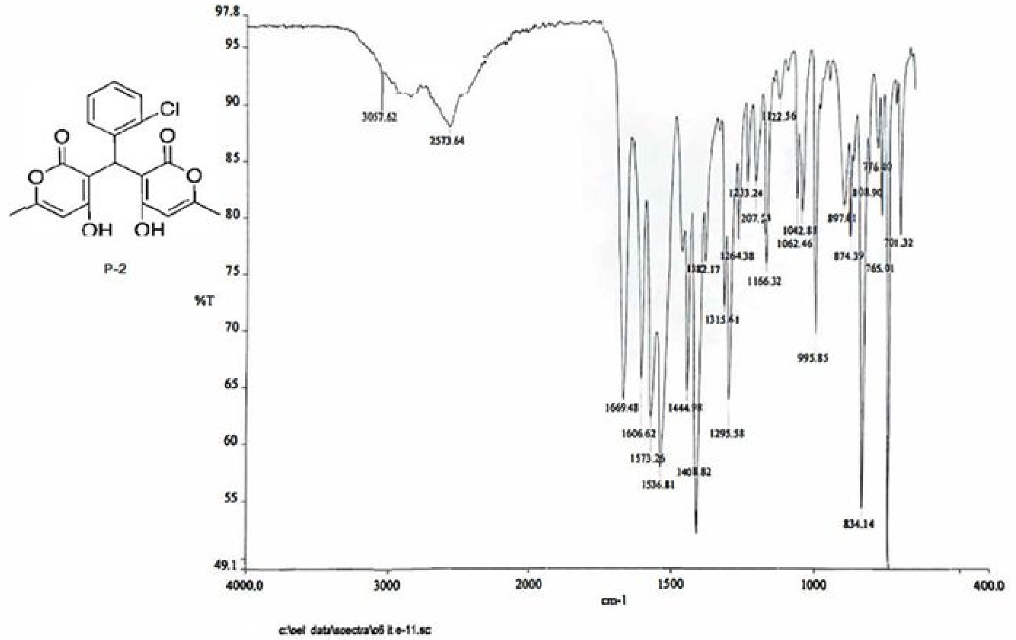

67

FigureS-67. IR spectrum of P-2.

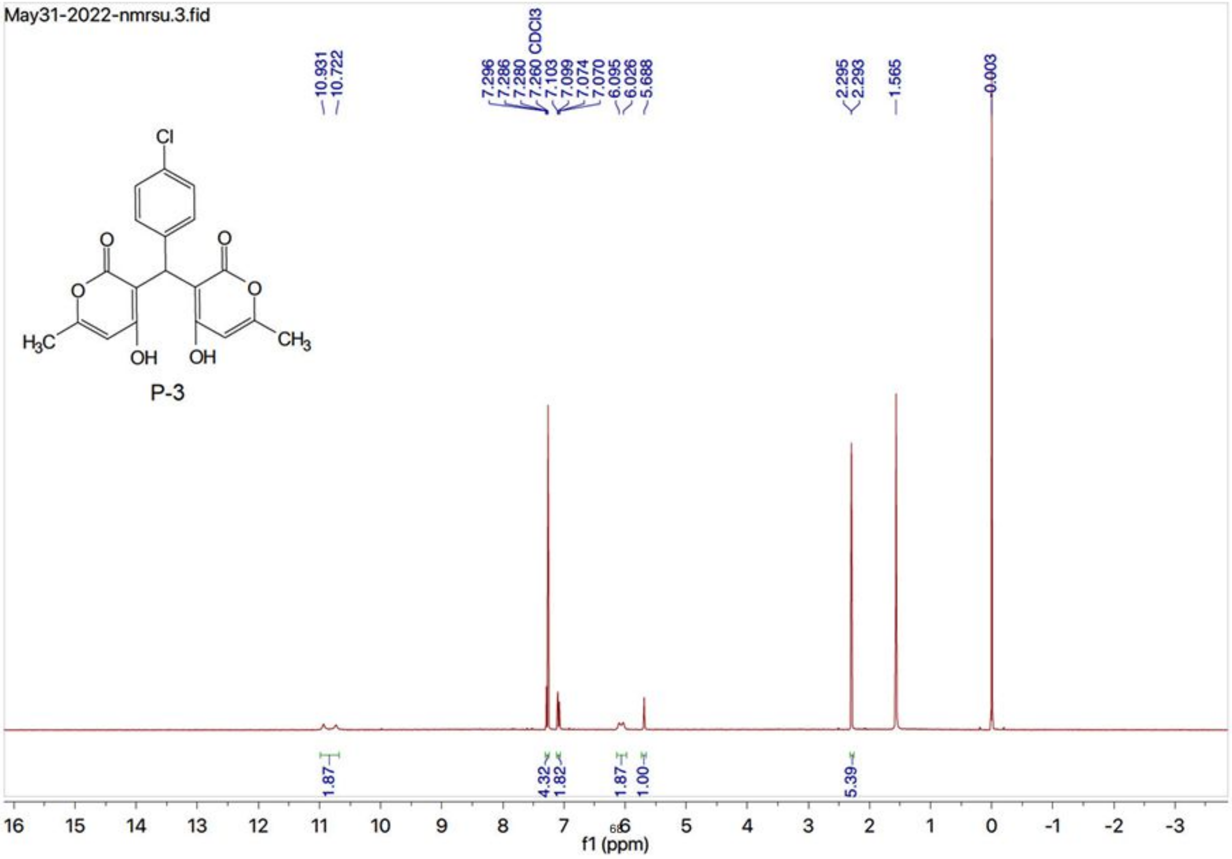

FigureS-68. ^{1}H NMR spectrum of P-3 in $CDCl_3$.

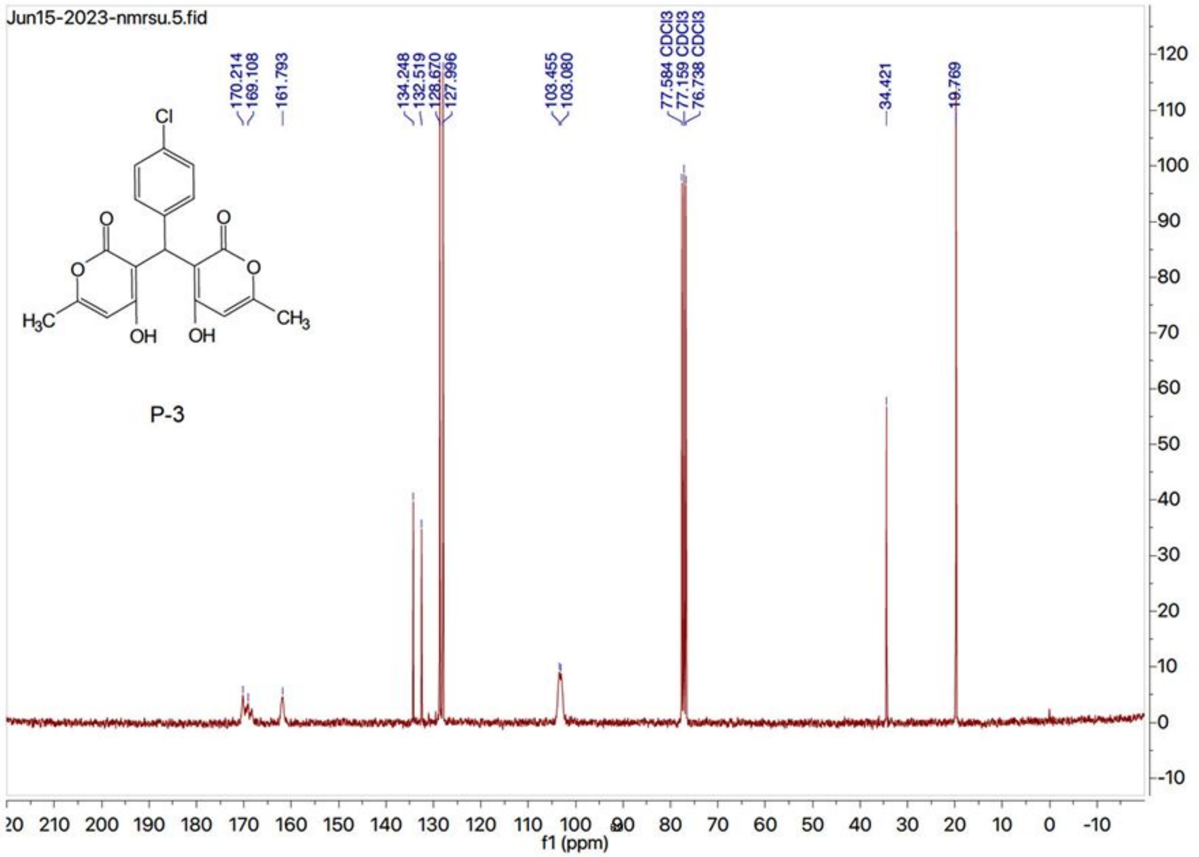

FigureS-69. ^{13}C NMR spectrum of P-3 in $CDCl_3$.

U of M
University of Minnesota Department of Chemistry
Mass Spectrometry Service Laboratory
email: chmmslab@umn.edu

Submit Sample To: Mass Spectrometry Facility
207 Pleasant St. SE
Minneapolis, MN 55455
Phone: (612)-625-8099
FAX : (612)-626-7541

Name: Julio Tapia	Phone: [redacted] Date: 6/12/2023
Email: tapiahjs @ augsburg.edu Email Results? Y / N	FAX: FAX Results? Y / N
P.I./Advisor: Michael Wentzel	U of M Budget #
Company/University: Augsburg University	P.O.# (For non-U of M Clients) Augsburg University Chemistry Department
Shipping Address:	Billing Address: 2211 Riverside Ave, Minneapolis, MN 55454
Sample Label: P3	Molecular Weight: 374.77
Structural Formula or Sample Composition:	Molecular Formula: $C_{19}H_{15}ClO_6$
	Melting/Boiling Point:
	Solubility:
	Thermal Stability:
	Toxicity:
	Reactivity:

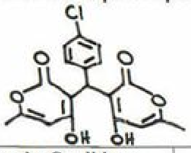

Chromatography Conditions:

Special Sample Considerations:

Analysis Requested	EI	CI	MALDI	ESI
Low Resolution Nominal Mass				
High Resolution Accurate Mass				√
+Ve				
-Ve				
GCMS				
LCMS				

Instrument Used		Conditions Used
Finnigan MAT 95		Source Temp:
Bruker Reflex III		Acc. Voltage:
Bruker BioTOF II		Resolution:
Waters Triple Quad		Scan Range:
Waters Synapt G2		Gas Used:

Operator Comments
Pos mode, MeOH solvent
PEG300 calibrant
M+Na theoretical: 397.0449
observed: 397.0435
error: 3.63 ppm

Log #:	Analyst:	Analysis Date:	Analyses Run:	Total Cost:

130180
P2
oesi
madeline honig
6/15/2023 2:28:38 PM

70

FigureS-70. HRMS results of P-3

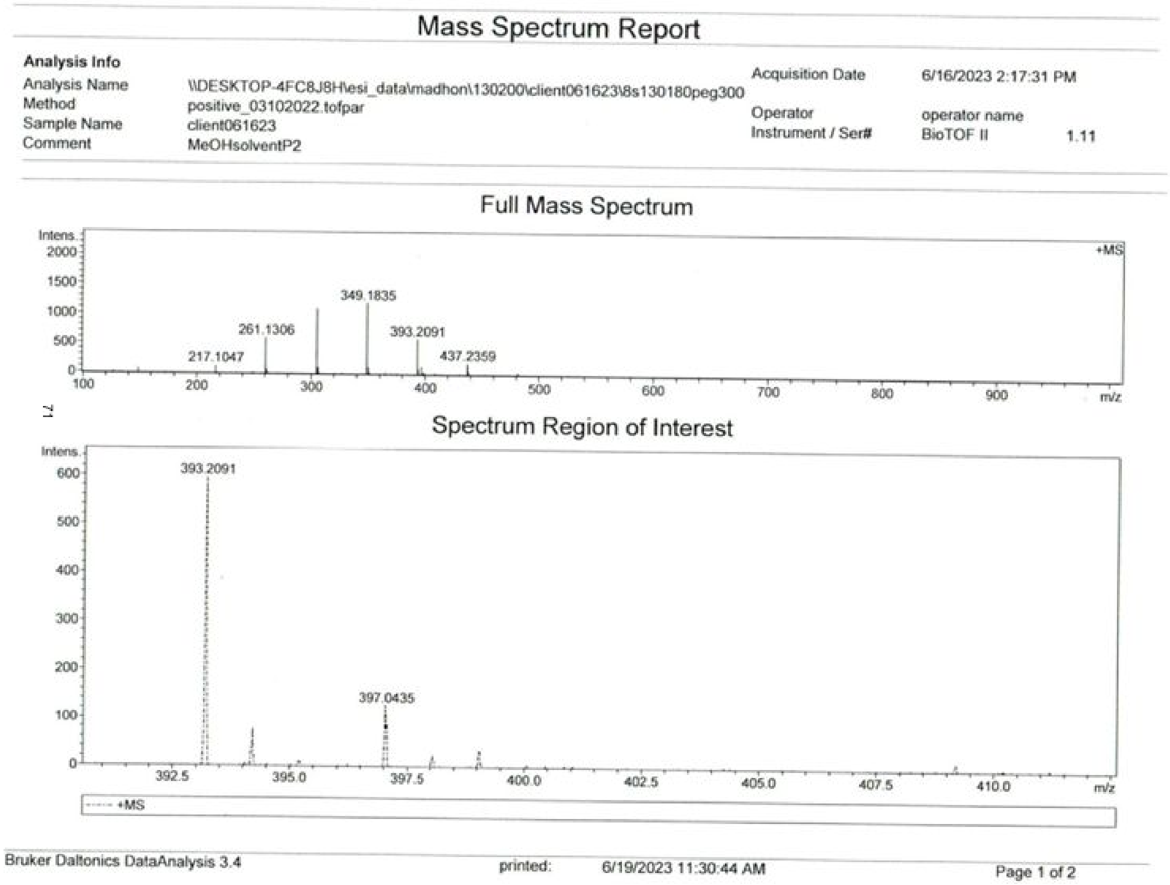

FigureS-71. HRMS results of P-3 spectrum

Mass Spectrum Report

Elemental Composition Report

Generate Molecular Formula Parameter

Formula, min.	C0H15Cl1O6						
Formula, max.	C19H16NaClO6						
Measured m/z	397.043	Tolerance	50	ppm		Charge	1
Check Valence	no	Minimum	0			Maximum	0
Nirogen Rule	no	Electron Configuration		both			
Filter H/C Ratio	no	Minimum	0			Maximum	3
Estimate Carbon	yes						

Sum Formula	Sigma	m/z	Err [ppm]	Mean Err [ppm]	Err [mDa]	rdb	N Rule	e⁻
C 19 H 15 Cl 1 Na 1 O 6	0.186	397.0449	3.63	3.52	1.44	11.50	ok	even

Mass Spectrum Peak List

72

#	m/z	Area	Res.	S/N
1	217.1047	4	6760	9.3
2	261.1306	19	8700	36.2
3	305.1573	38	9630	58.5
4	306.1626	4	11656	5.8
5	349.1835	50	8817	30.3
6	350.1849	5	7866	2.6
7	393.2091	27	9305	16.0
8	397.0435	5	8749	3.7
9	437.2359	8	11494	21.4

Bruker Daltonics DataAnalysis 3.4 printed: 6/19/2023 11:30:44 AM Page 2 of 2

FigureS-72. HRMS results of P-3 peak list.

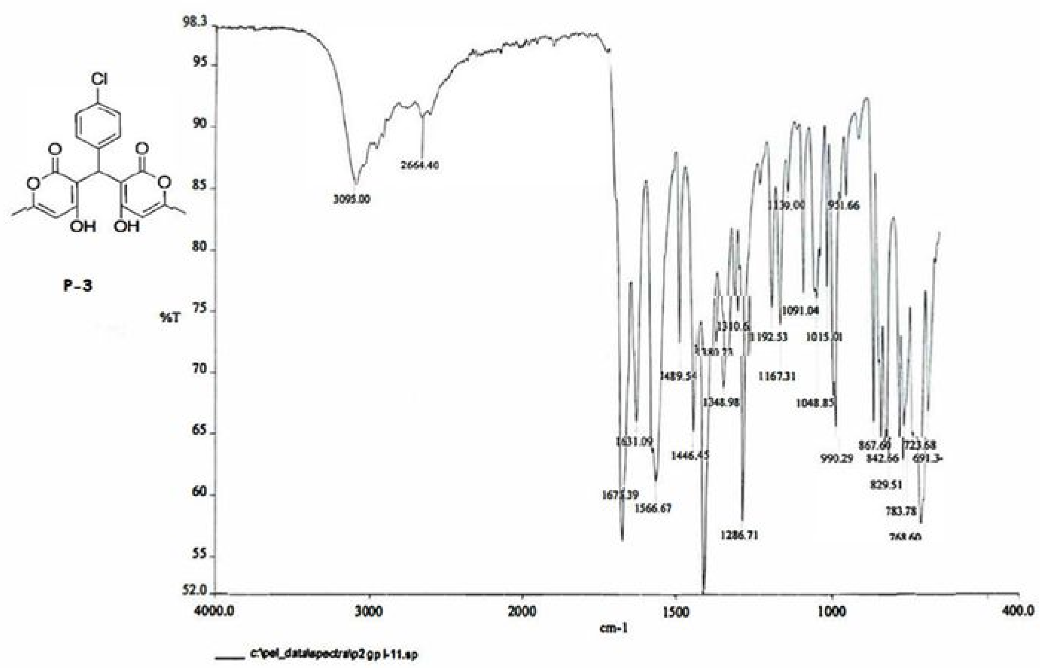

73

FigureS-73. IR spectrum of P-3

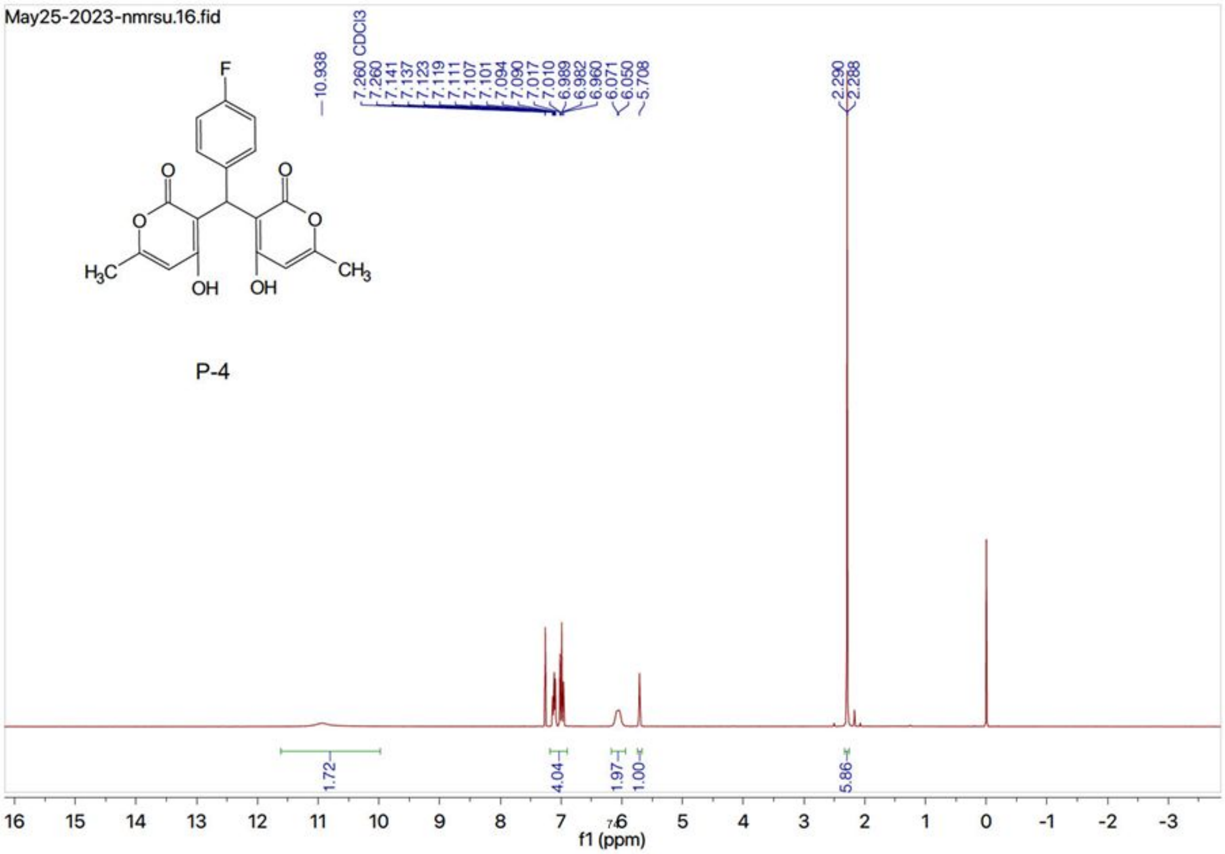

FigureS-74. ¹H NMR spectrum of P-4 in $CDCl_3$.

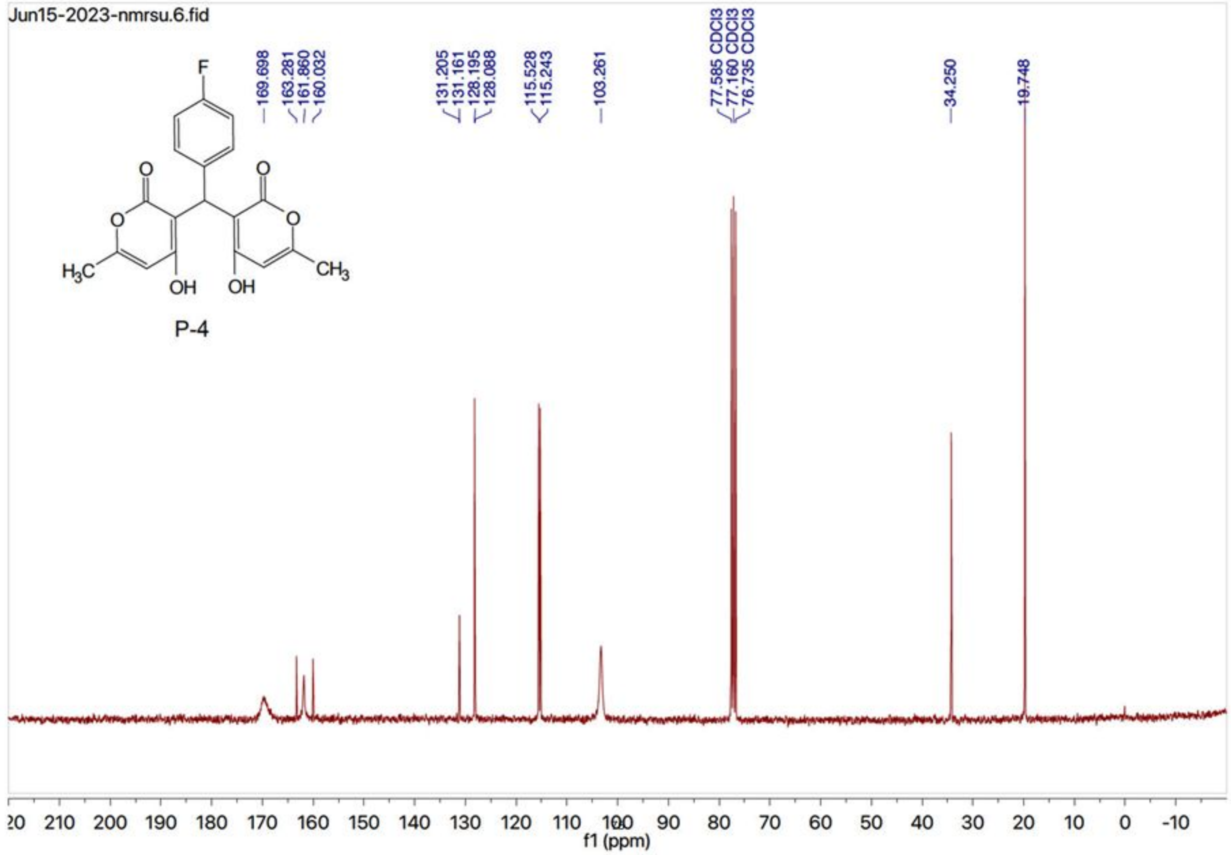

FigureS-75. ^{13}C NMR spectrum of P-4 in $CDCl_3$

U of M
University of Minnesota Department of Chemistry
Mass Spectrometry Service Laboratory
email: chmmslab@umn.edu

Submit Sample To: Mass Spectrometry Facility
207 Pleasant St. SE
Minneapolis, MN 55455
Phone: (612)-625-8099
FAX : (612)-626-7541

Name: Julio Tapia	Phone: [redacted] Date: 6/12/2023
Email: tapiahJS @augsburg.edu Email Results? Y / N	FAX: FAX Results? Y / N
P.I./Advisor: Michael Wentzel	U of M Budget #
Company/University: Augsburg University	P.O.# (For non-U of M Clients) 2211 Riverside Ave, Mineapolis, MN 55454
Shipping Address:	Billing Address:
Sample Label: P4	Molecular Weight: 358.32
Structural Formula or Sample Composition:	Molecular Formula: $C_{19}H_{15}FO_6$
	Melting/Boiling Point:
	Solubility:
	Thermal Stability:
	Toxicity:
	Reactivity:

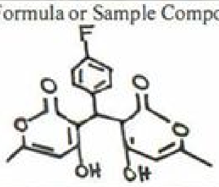

Chromatography Conditions:

Special Sample Considerations:

Analysis Requested

	EI	CI	MALDI	ESI
Low Resolution Nominal Mass				
High Resolution Accurate Mass				√
+Ve				
-Ve				
GCMS				
LCMS				

Instrument Used	Conditions Used	Operator Comments
Finnigan MAT 95	Source Temp:	pos mode. MeOH solvent
Bruker Reflex III	Acc. Voltage:	PEG300 calibrant
Bruker BioTOF II	Resolution:	M+Na theoretical 381.0745
Waters Triple Quad	Scan Range:	observed 381.0751
Waters Synapt G2	Gas Used:	error -1.60 ppm

Log #:	Analyst:	Analysis Date:	Analyses Run:	Total Cost:

130181
P3
oesi
madeline honig
6/15/2023 2:30:45 PM

76

FigureS-76. HRMS results of P-4

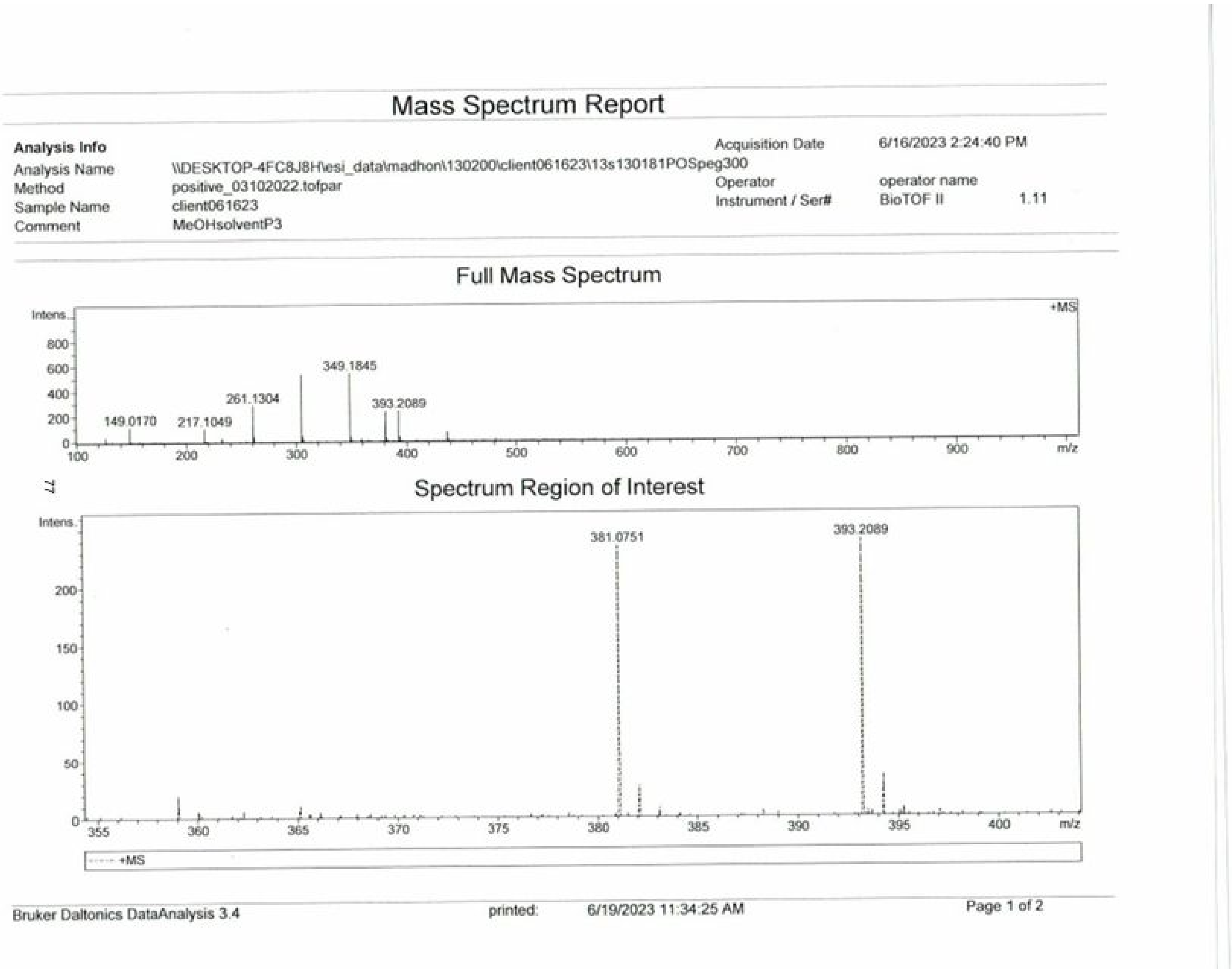

FigureS-77. HRMS results of P-4 spectrum.

Mass Spectrum Report

Elemental Composition Report

Generate Molecular Formula Parameter

Formula, min.	C0H15F1O6						
Formula, max.	C19H16NaFO6						
Measured m/z	381.075	Tolerance	10	ppm		Charge	1
Check Valence	no	Minimum	0			Maximum	0
Nirogen Rule	no	Electron Configuration		both			
Filter H/C Ratio	no	Minimum	0			Maximum	3
Estimate Carbon	yes						

Sum Formula	Sigma	m/z	Err [ppm]	Mean Err [ppm]	Err [mDa]	rdb	N Rule	e⁻
C 19 H 15 F 1 Na 1 O 6	0.122	381.0745	-1.60	-1.58	-0.61	11.50	ok	even

Mass Spectrum Peak List

#	m/z	Area	Res.	S/N
1	149.0170	2	8103	8.2
2	217.1049	3	9639	7.8
3	261.1304	11	7511	14.5
4	305.1567	21	8191	51.2
5	349.1845	20	11250	22.4
6	381.0751	9	11204	7.8
7	393.2089	11	9182	9.5

Bruker Daltonics DataAnalysis 3.4 printed: 6/19/2023 11:34:25 AM Page 2 of 2

FigureS-78. HRMS results of C-1 peak list.

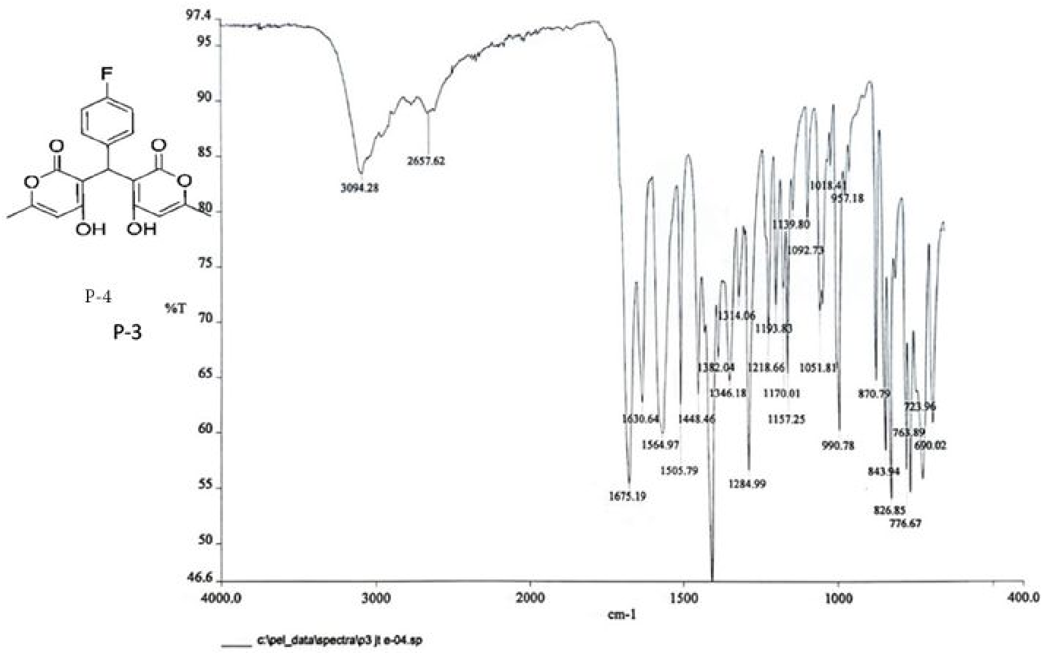

79

FigureS-79. IR spectrum of P-3.

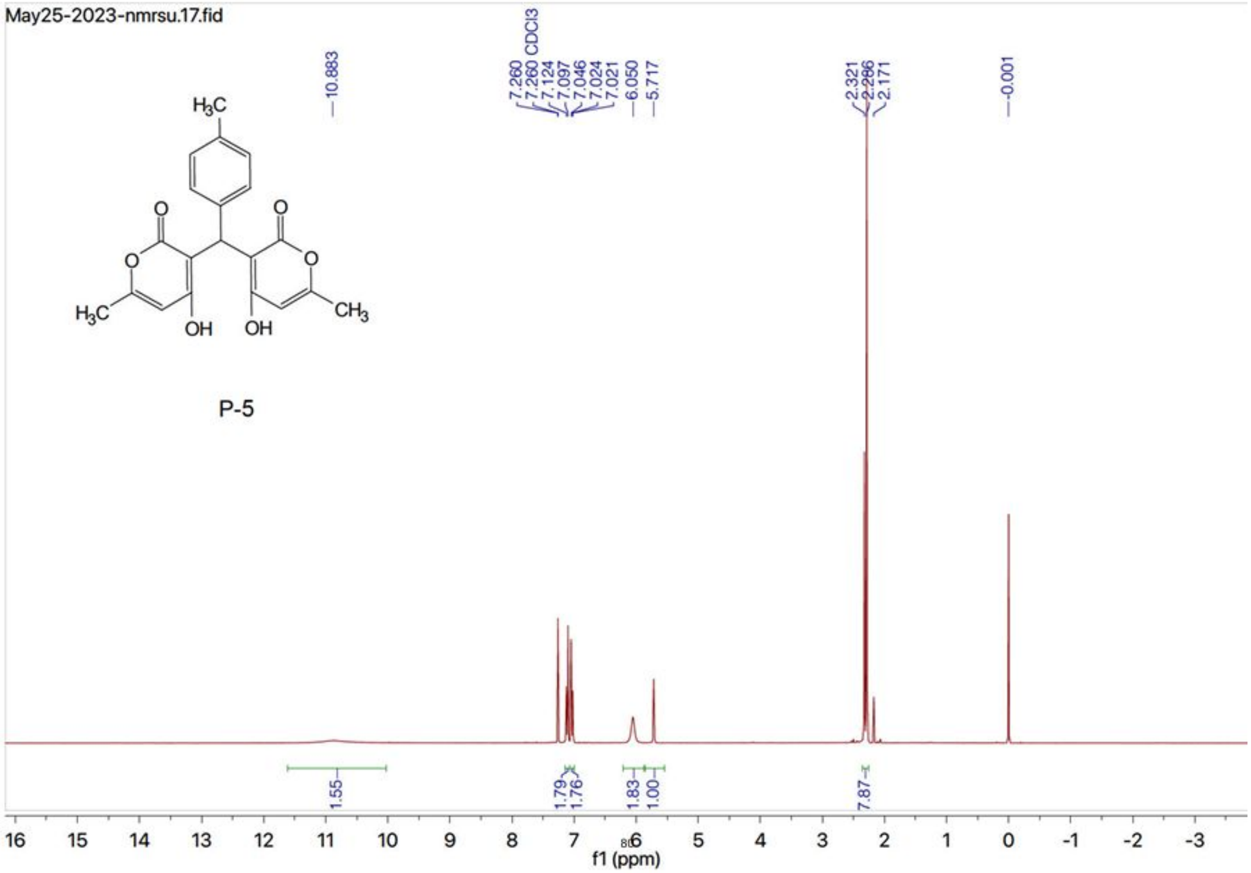

FigureS-80. ^{1}H NMR spectrum of P-5 in $CDCl_3$.

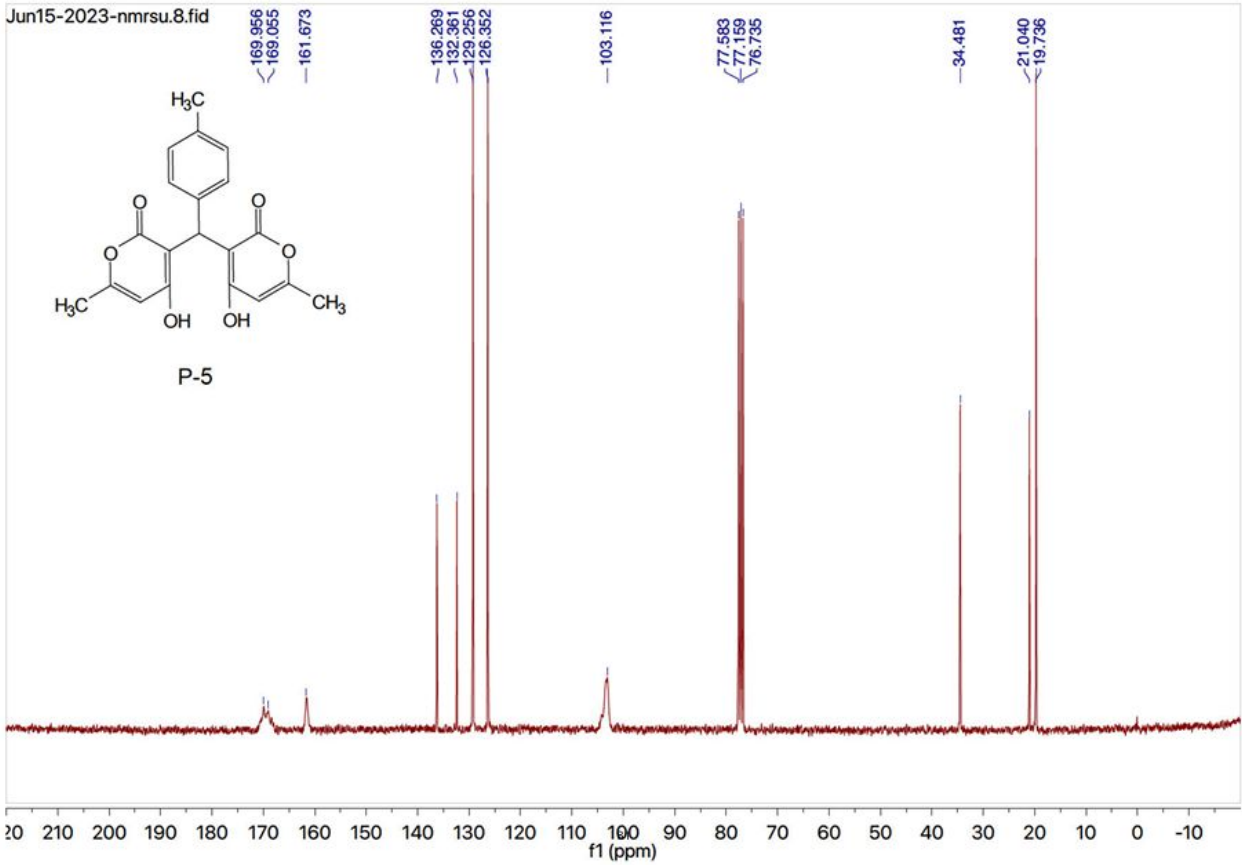

FigureS-81. ^{13}C NMR spectrum of P-5 in $CDCl_3$.

U of M

University of Minnesota Department of Chemistry
Mass Spectrometry Service Laboratory
email: chmmslab@umn.edu

Submit Sample To: Mass Spectrometry Facility
207 Pleasant St. SE
Minneapolis, MN 55455
Phone: (612)-625-8099
FAX : (612)-626-7541

Name: Julio Tapia	Phone: [redacted] Date: 6/12/2023
Email: tapiahjs@augsburg.edu Email Results? Ⓨ/N	FAX: FAX Results? Y / N
P.I./Advisor: Michael Wentzel	U of M Budget #
Company/University: Augsburg University	P.O.# (For non-U of M Clients) Augsburg University Chemistry Department
Shipping Address:	Billing Address: 2211 Riverside Ave, Minneapolis, MN 55454
Sample Label: P5	Molecular Weight: 354.36
Structural Formula or Sample Composition:	Molecular Formula: $C_{20}H_{18}O_6$
	Melting/Boiling Point:
	Solubility:
	Thermal Stability:
	Toxicity:
	Reactivity:

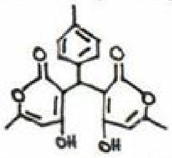

Chromatography Conditions:

Analysis Requested

	EI	CI	MALDI	ESI
Low Resolution Nominal Mass				
High Resolution Accurate Mass				√
+Ve				
-Ve				
GCMS				
LCMS				

Special Sample Considerations:

Instrument Used		Conditions Used	Operator Comments
Finnigan MAT 95		Source Temp:	POS mode. MeOH solvent
Bruker Reflex III		Acc. Voltage:	PEG400 calibrant
Bruker BioTOF II		Resolution:	M+Na theoretical 377.0996
Waters Triple Quad		Scan Range:	observed 377.0988
Waters Synapt G2		Gas Used:	error 1.99 ppm

Log #:	Analyst:	Analysis Date:	Analyses Run:	Total Cost:

130182
P4
oesi
madeline honig
6/15/2023 2:31:47 PM

82

FigureS-82. HRMS results of P-5

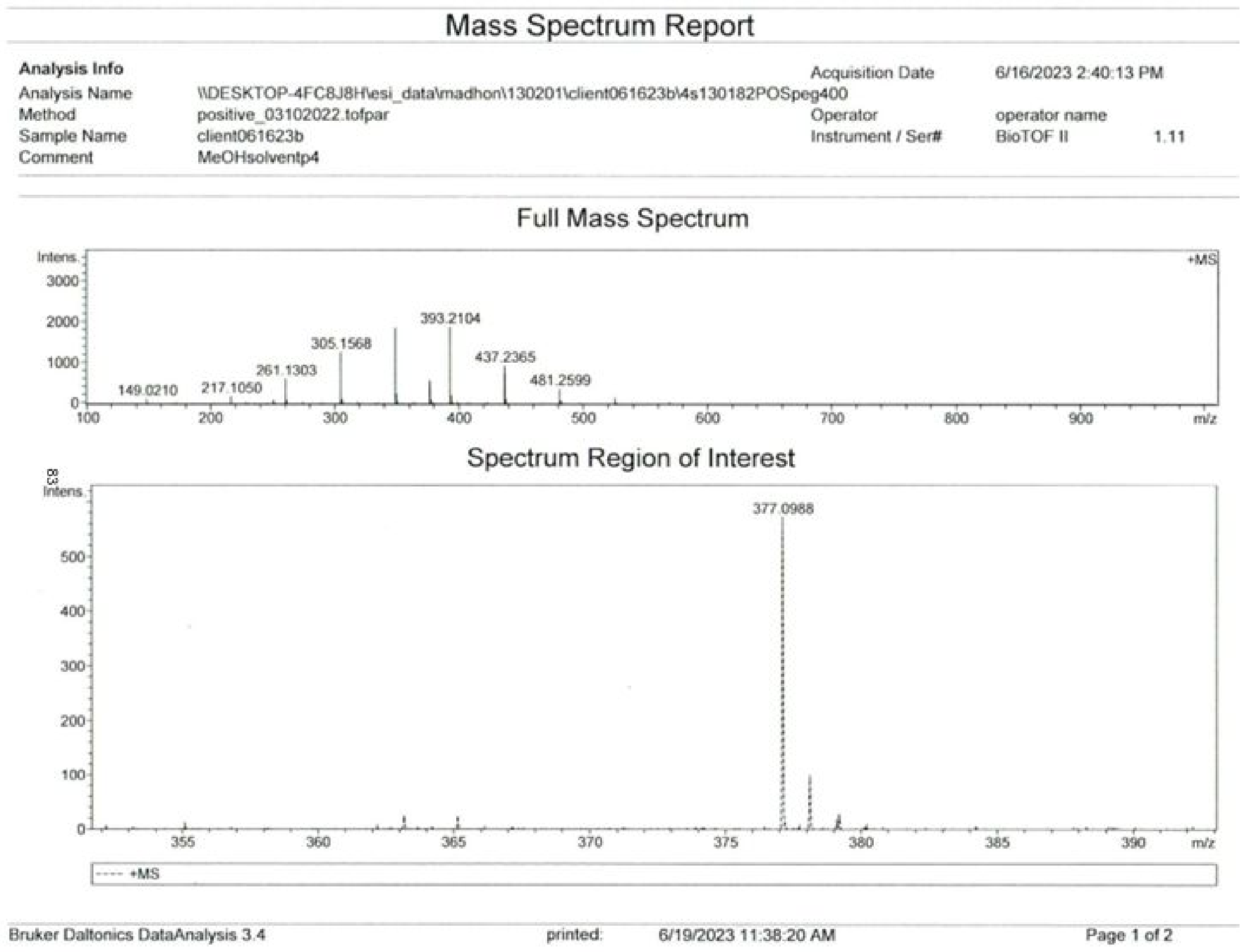

FigureS-83. HRMS results of P-5 spectrum.

Mass Spectrum Report

Elemental Composition Report

Generate Molecular Formula Parameter

Formula, min.	C0H18Na1O6						
Formula, max.	C20H18O6Na						
Measured m/z	377.099	Tolerance	10	ppm		Charge	1
Check Valence	no	Minimum	0			Maximum	0
Nirogen Rule	no	Electron Configuration		both			
Filter H/C Ratio	no	Minimum	0			Maximum	3
Estimate Carbon	yes						

Sum Formula	Sigma	m/z	Err [ppm]	Mean Err [ppm]	Err [mDa]	rdb	N Rule	e⁻
C 20 H 18 Na 1 O 6	0.129	377.0996	1.99	1.99	0.75	11.50	ok	even

Mass Spectrum Peak List

84

#	m/z	Area	Res.	S/N
1	149.0210	3	6904	7.8
2	217.1050	6	7234	8.5
3	261.1303	19	9563	20.6
4	305.1568	47	8861	36.1
5	306.1623	4	7030	3.1
6	349.1833	85	8201	34.6
7	350.1878	9	10426	4.4
8	377.0988	28	8399	9.4
9	393.2104	90	8519	39.4
10	394.2110	9	8713	4.3
11	437.2365	51	8097	64.3
12	438.2383	6	8337	7.2
13	481.2599	21	9270	35.5
14	525.2889	7	10660	16.6

Bruker Daltonics DataAnalysis 3.4 printed: 6/19/2023 11:38:20 AM Page 2 of 2

FigureS-84. HRMS results of P-5 peak list.

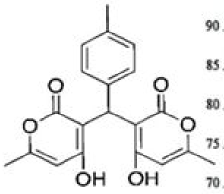

P-5

P-4

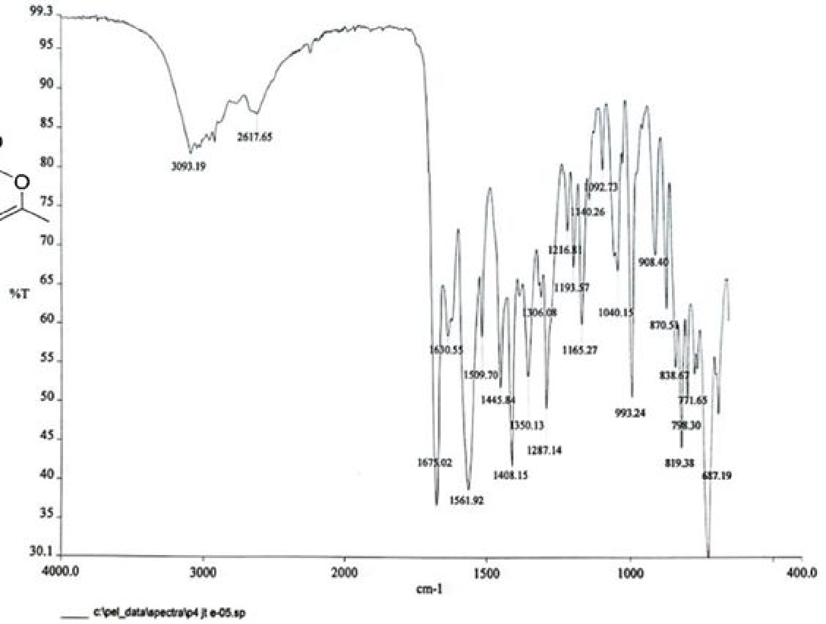

85

FigureS-85. IR spectrum of P-5.

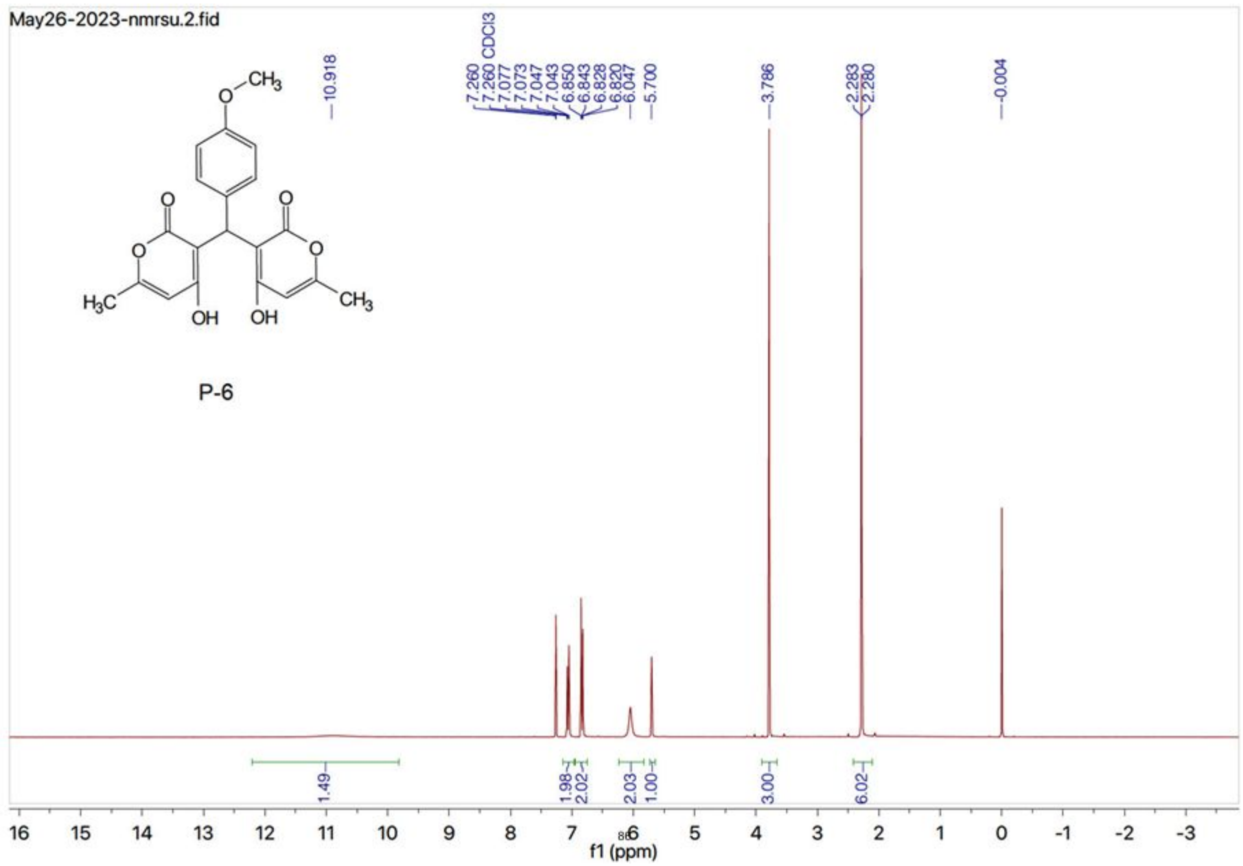

FigureS-86. ^{1}H NMR spectrum of P-6 in $CDCl_3$.

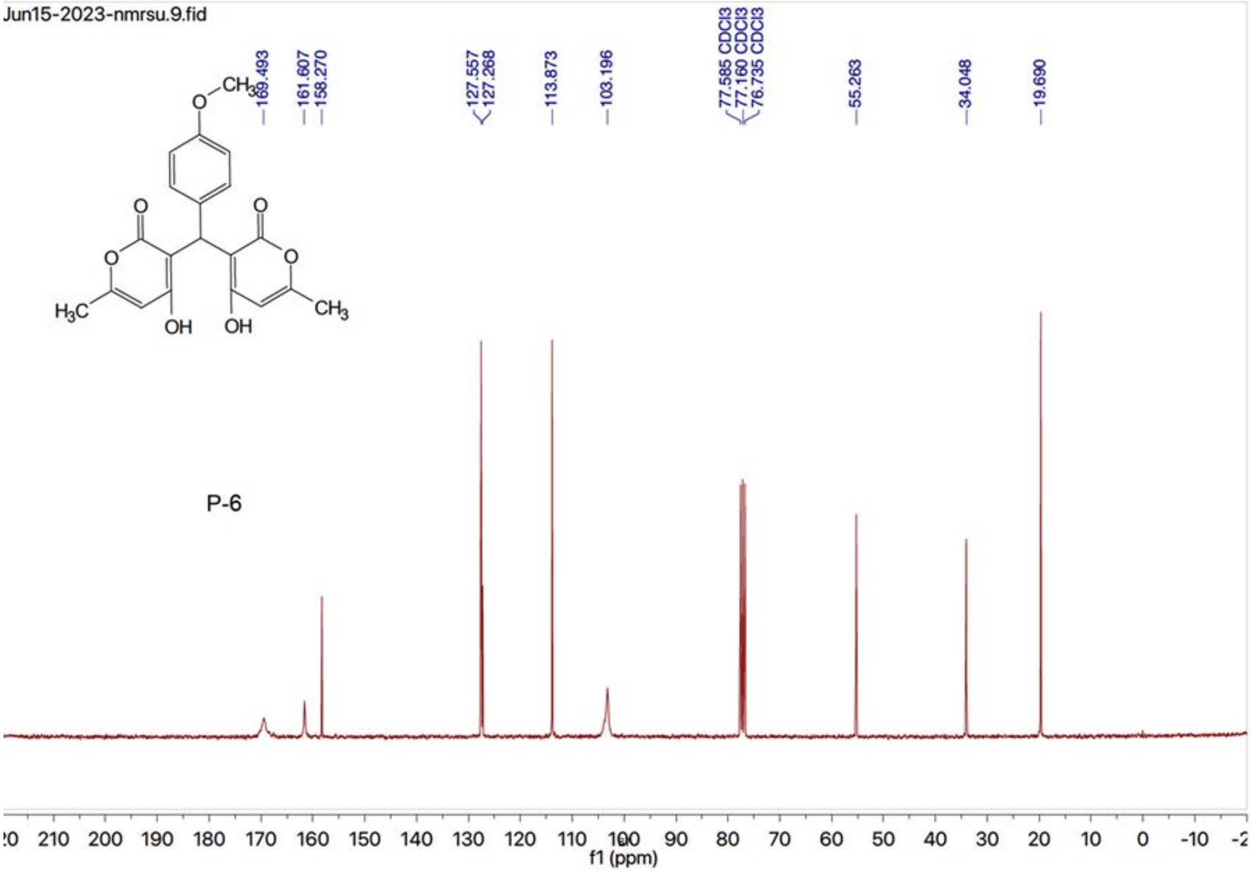

FigureS-87. ^{13}C NMR spectrum of P-6 in $CDCl_3$.

U of M
University of Minnesota Department of Chemistry
Mass Spectrometry Service Laboratory
email: chmmslab@umn.edu

Submit Sample To: Mass Spectrometry Facility
207 Pleasant St. SE
Minneapolis, MN 55455
Phone: (612)-625-8099
FAX : (612)-626-7541

Name: Julio Tapia	Phone: [redacted] Date: 6/12/2023
Email: tapiahjs @ augsburg.edu Email Results? Y / N	FAX: FAX Results? Y / N
P.I./Advisor: Michael Wentzel	U of M Budget #
Company/University: Augsburg University	P.O.# (For non-U of M Clients) Augsburg University Chemistry Department
Shipping Address:	Billing Address: 2211 Riverside Ave, Minneapolis, MN 55454
Sample Label: P6	Molecular Weight: 370.36
Structural Formula or Sample Composition:	Molecular Formula: $C_{20}H_{18}O_7$
	Melting/Boiling Point:
	Solubility:
	Thermal Stability:
	Toxicity:
	Reactivity:

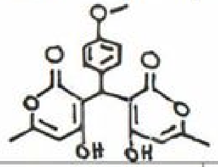

Chromatography Conditions:

Special Sample Considerations:

Analysis Requested

	EI	CI	MALDI	ESI
Low Resolution Nominal Mass				
High Resolution Accurate Mass				√
+Ve				
-Ve				
GCMS				
LCMS				

Instrument Used		Conditions Used
Finnigan MAT 95		Source Temp:
Bruker Reflex III		Acc. Voltage:
Bruker BioTOF II		Resolution:
Waters Triple Quad		Scan Range:
Waters Synapt G2		Gas Used:

Operator Comments
POS mode. MeOH solvent
PPG425 calibrant
M+Na theoretical 393.0945
observed 393.0945
error ·0.12 ppm

Log #:	Analyst:	Analysis Date:	Analyses Run:	Total Cost:

130183
P5
oesi
madeline honig
6/15/2023 2:33:04 PM

88

FigureS-88. HRMS results of P-6

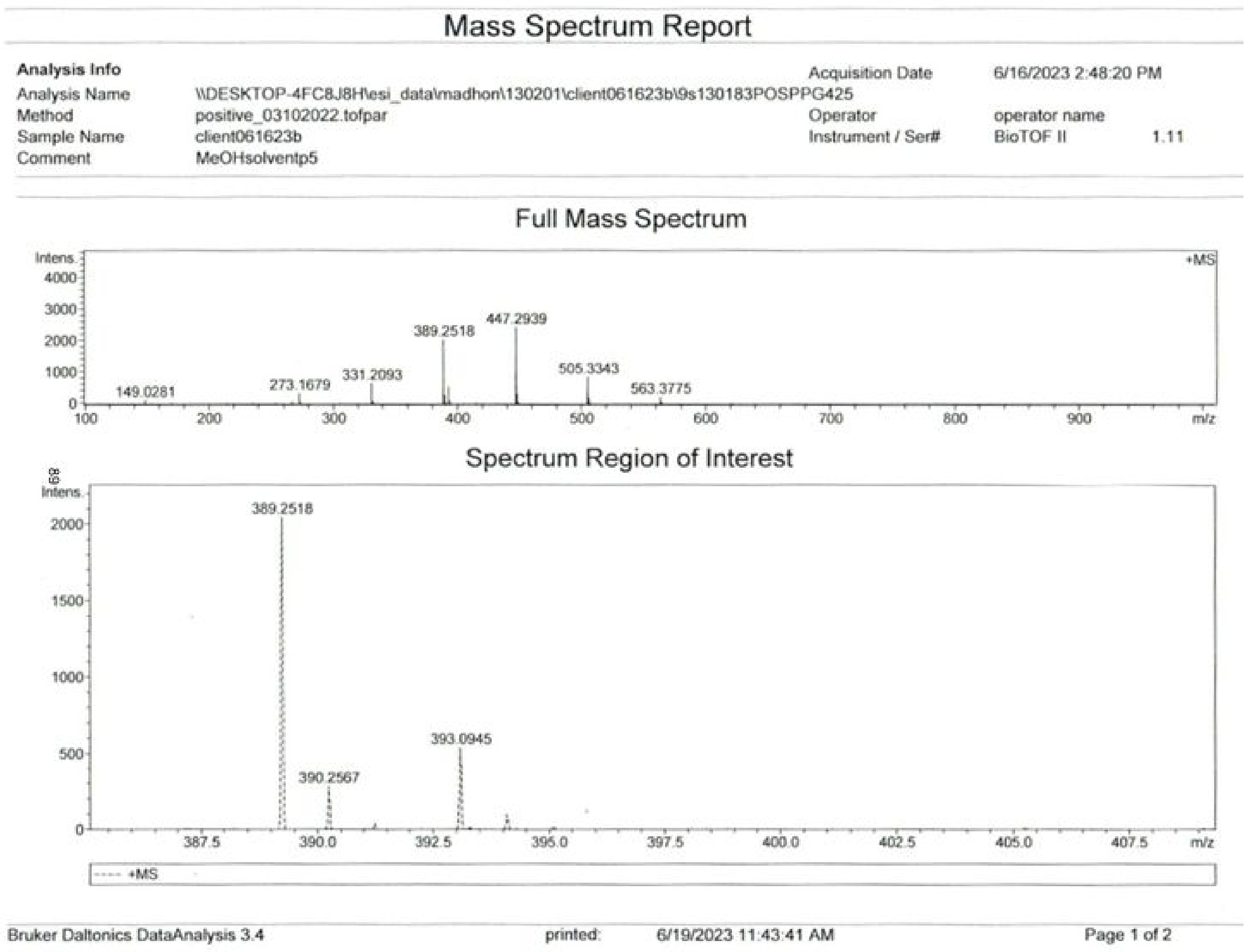

FigureS-89. HRMS results of P-6 spectrum.

Mass Spectrum Report

Elemental Composition Report

Generate Molecular Formula Parameter

Formula, min.	C0H18Na1O6						
Formula, max.	C20H18O7Na						
Measured m/z	393.094	Tolerance	10	ppm		Charge	1
Check Valence	no	Minimum	0			Maximum	0
Nirogen Rule	no	Electron Configuration		both			
Filter H/C Ratio	no	Minimum	0			Maximum	3
Estimate Carbon	yes						

Sum Formula	Sigma	m/z	Err [ppm]	Mean Err [ppm]	Err [mDa]	rdb	N Rule	e⁻
C 20 H 18 Na 1 O 7	0.129	393.0945	-0.12	-0.12	-0.05	11.50	ok	even

Mass Spectrum Peak List

#	m/z	Area	Res.	S/N
1	149.0281	3	7664	14.9
2	273.1679	12	7954	15.3
3	331.2093	31	7776	17.9
4	389.2518	101	8375	39.4
5	390.2567	13	8606	5.5
6	393.0945	26	8501	10.7
7	447.2939	129	9255	91.0
8	448.2961	18	7930	11.0
9	505.3343	48	10140	137.1
10	506.3424	9	10756	30.6
11	563.3775	13	12320	33.8

90

Bruker Daltonics DataAnalysis 3.4 printed: 6/19/2023 11:43:41 AM Page 2 of 2

FigureS-90. HRMS results of P-6 peak list.

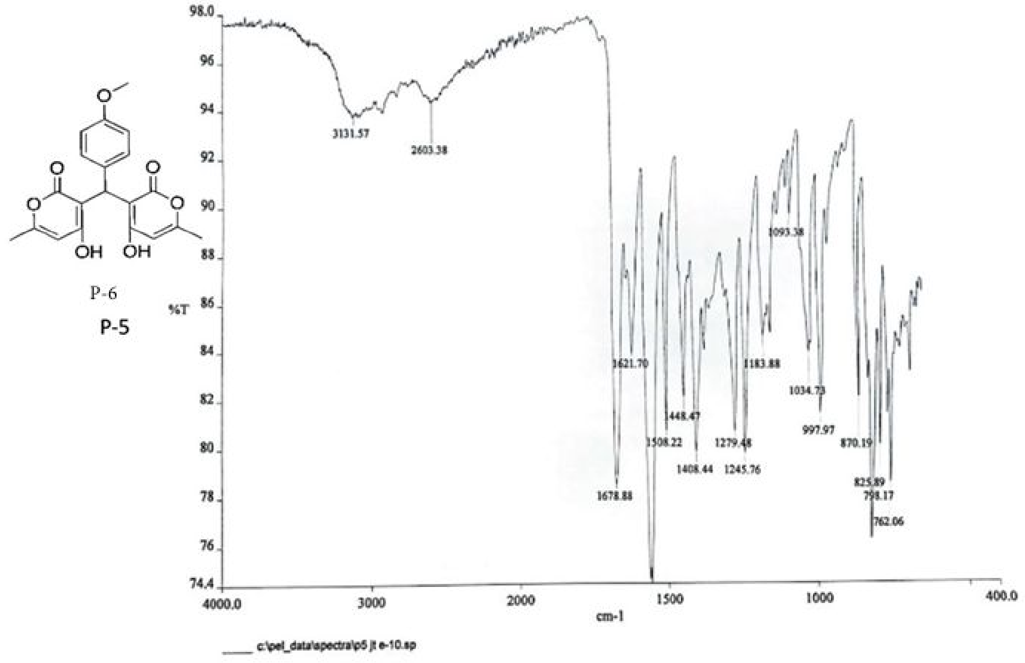

91

FigureS-91. IR spectrum of P-6.

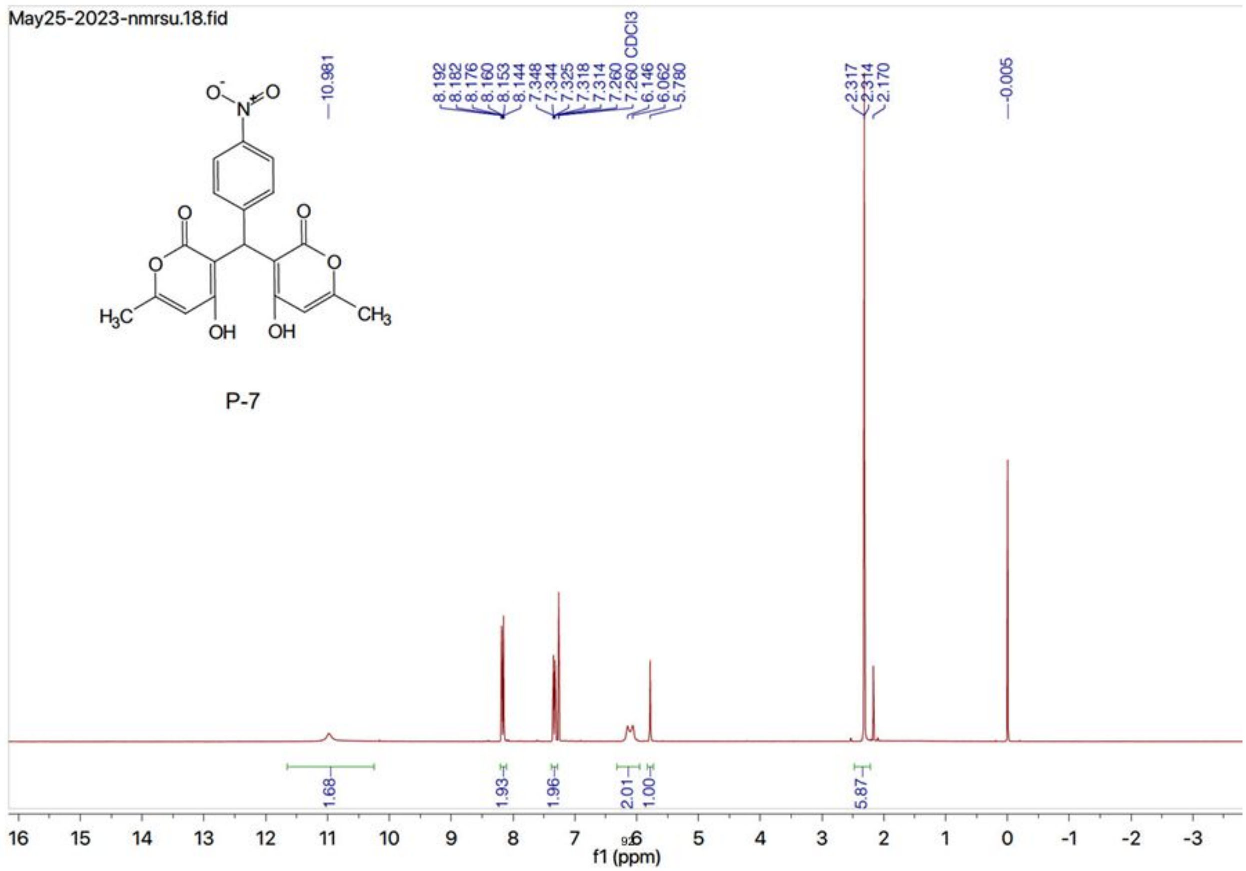

FigureS-92. ^{1}H NMR spectrum of P-7 in $CDCl_3$.

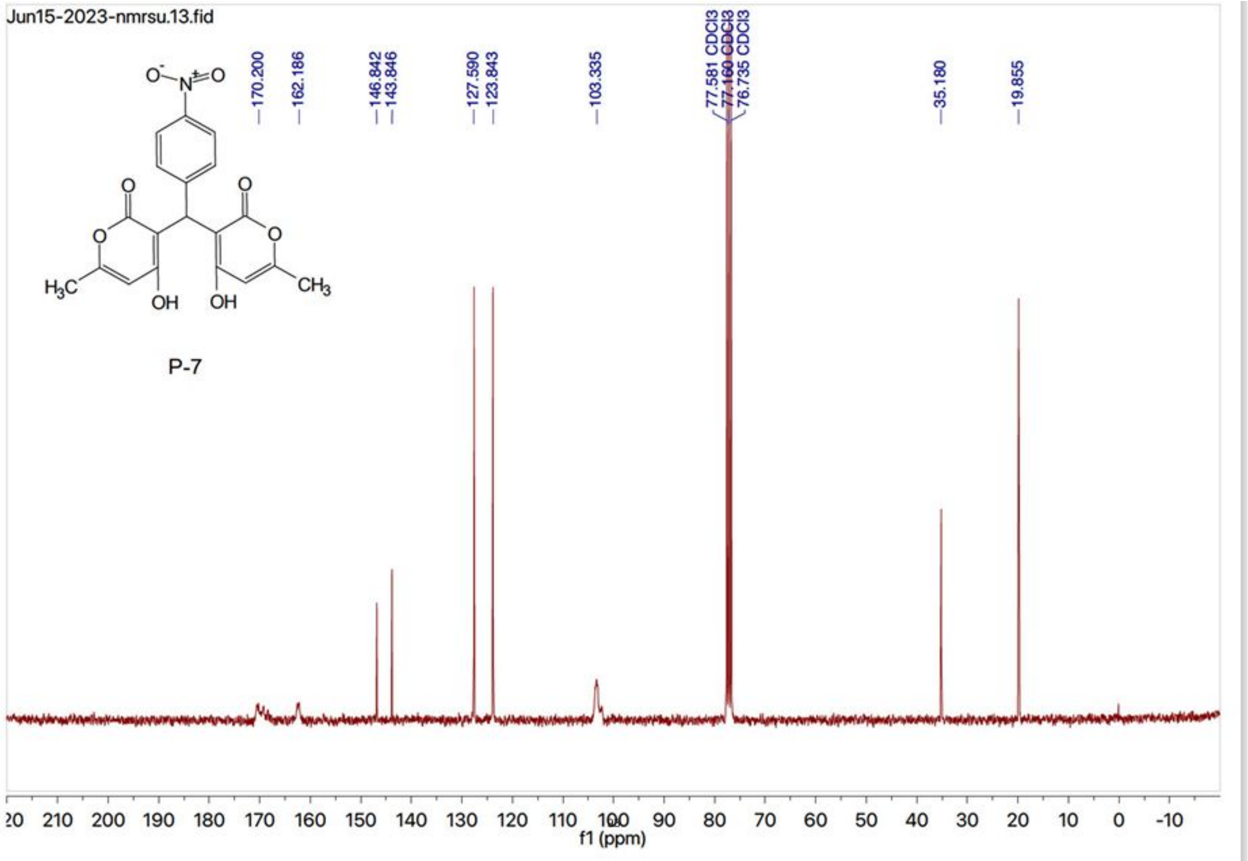

FigureS-93. ^{13}C NMR spectrum of P-7 in $CDCl_3$.

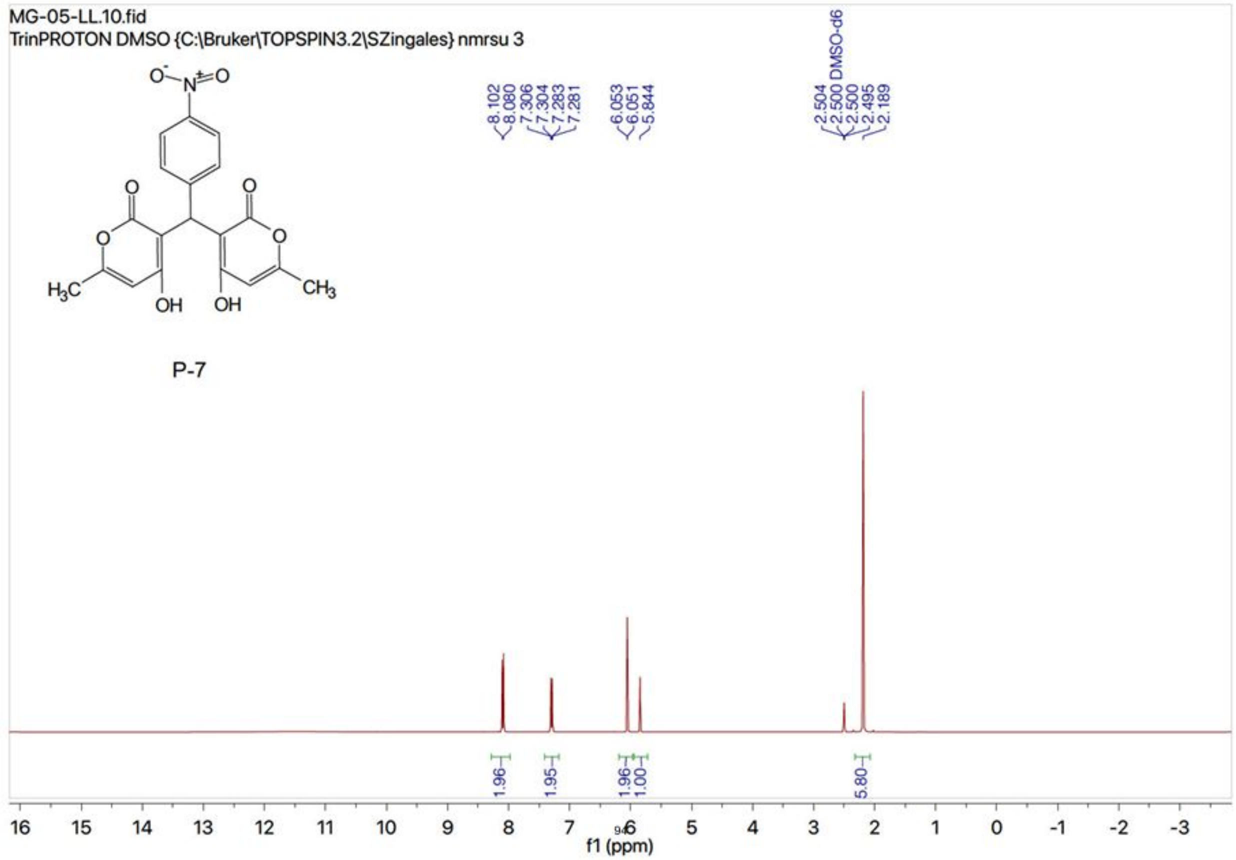

FigureS-94. DMSO-d_6 1H NMR spectra of P-7

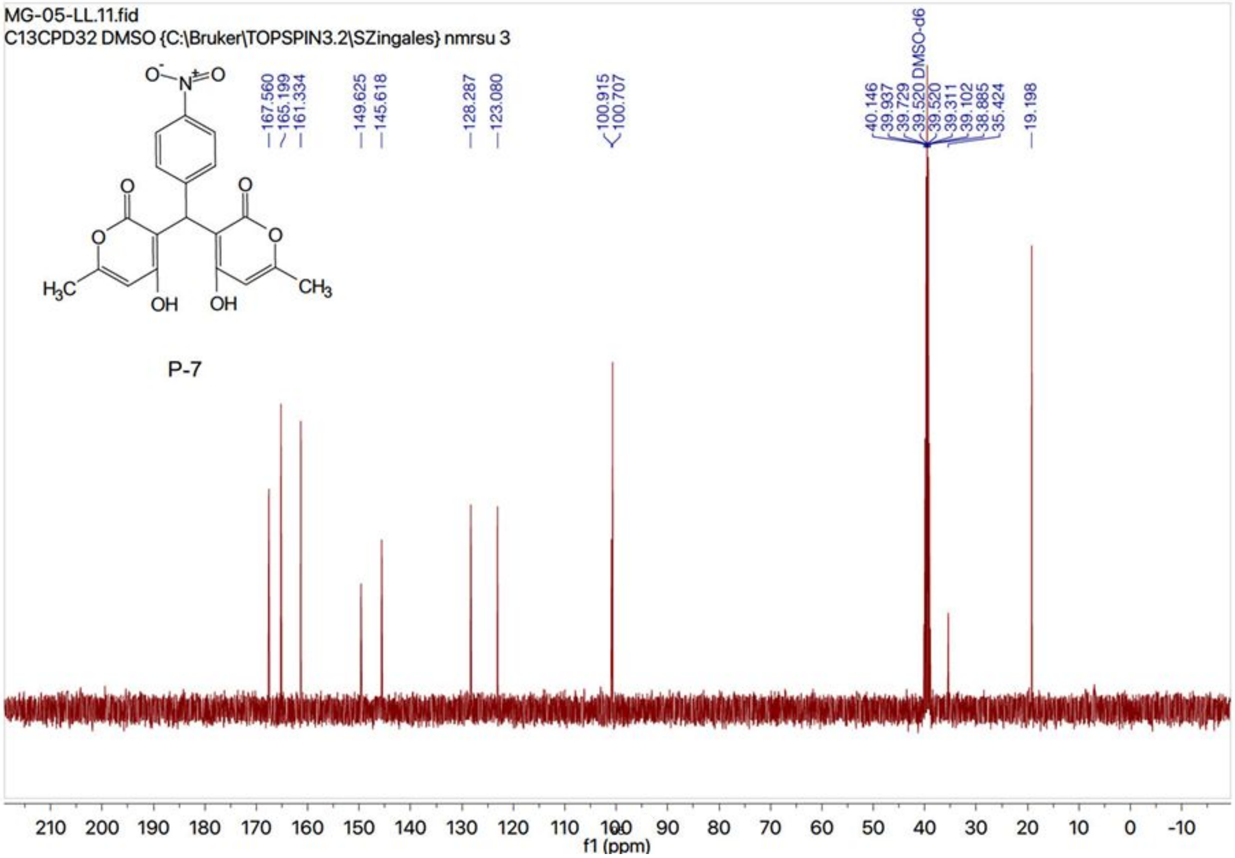

FigureS-95. DMSO-d_6 13C NMR spectra of P-7

U of M
University of Minnesota Department of Chemistry
Mass Spectrometry Service Laboratory
email: chmmslab@umn.edu

Submit Sample To: Mass Spectrometry Facility
207 Pleasant St. SE
Minneapolis, MN 55455
Phone: (612)-625-8099
FAX : (612)-626-7541

Name: Julio Tapia	Phone: [redacted] Date: 6/12/2023
Email: tapiahss@augsburg.edu Email Results? Y / N	FAX: FAX Results? Y / N
P.I./Advisor: Michael Wentzel	U of M Budget #
Company/University: Augsburg University	P.O.# (For non-U of M Clients) Augsburg University Chemistry Department
Shipping Address:	Billing Address: 2211 Riverside Ave, Minneapolis, MN 55454
Sample Label: P7	Molecular Weight: 385.33
Structural Formula or Sample Composition:	Molecular Formula: $C_{19}H_{15}NO_8$
	Melting/Boiling Point:
	Solubility:
	Thermal Stability:
	Toxicity:
	Reactivity:

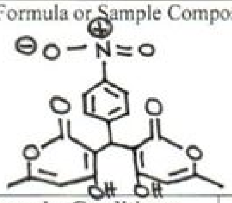

Chromatography Conditions:

Special Sample Considerations:

Analysis Requested

	EI	CI	MALDI	ESI
Low Resolution Nominal Mass				
High Resolution Accurate Mass				✓
+Ve				
-Ve				
GCMS				
LCMS				

Instrument Used		Conditions Used
Finnigan MAT 95		Source Temp:
Bruker Reflex III		Acc. Voltage:
Bruker BioTOF II		Resolution:
Waters Triple Quad		Scan Range:
Waters Synapt G2		Gas Used:

Operator Comments
Pos mode. MeOH solvent
PEG400 calibrant
M+Na+ theoretical 408.0690
observed 408.0684
error 1.46 ppm

Log #:	Analyst:	Analysis Date:	Analyses Run:	Total Cost:

130185
P7
oesi
madeline honig
6/15/2023 2:36:24 PM

96

FigureS-96. HRMS results of P-7

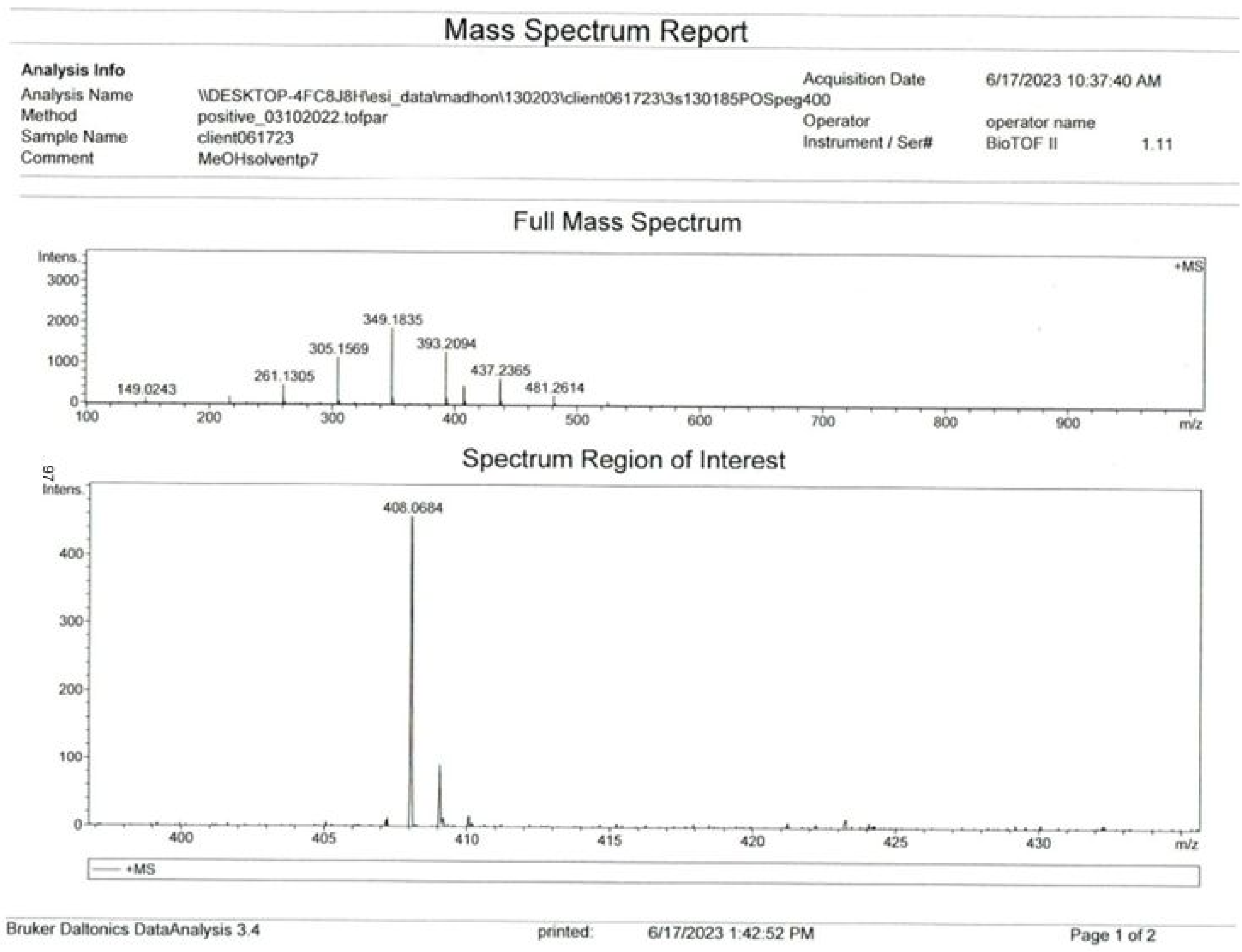

FigureS-97. HRMS results of P-7 spectrum.

Mass Spectrum Report

Elemental Composition Report

Generate Molecular Formula Parameter

Formula, min.	C0H15N1Na1O8						
Formula, max.	C19H15NO8Na						
Measured m/z	408.068	Tolerance	10	ppm		Charge	1
Check Valence	no	Minimum	0			Maximum	0
Nirogen Rule	no	Electron Configuration		both			
Filter H/C Ratio	no	Minimum	0			Maximum	3
Estimate Carbon	yes						

Sum Formula	Sigma	m/z	Err [ppm]	Mean Err [ppm]	Err [mDa]	rdb	N Rule	e⁻
C 19 H 15 N 1 Na 1 O 8	0.125	408.0690	1.46	1.45	0.60	12.50	ok	even

Mass Spectrum Peak List

#	m/z	Area	Res.	S/N
1	149.0243	2	7518	8.8
2	217.1049	5	8329	9.6
3	261.1305	17	7591	17.2
4	305.1569	47	7869	30.6
5	349.1835	80	8539	55.8
6	350.1856	7	7798	4.7
7	393.2094	66	8339	53.3
8	394.2123	8	8572	6.7
9	408.0684	25	8442	22.8
10	437.2365	31	9744	51.1
11	481.2614	12	9815	27.5

Bruker Daltonics DataAnalysis 3.4 printed: 6/17/2023 1:42:52 PM Page 2 of 2

FigureS-98. HRMS results of P-7 peak list.

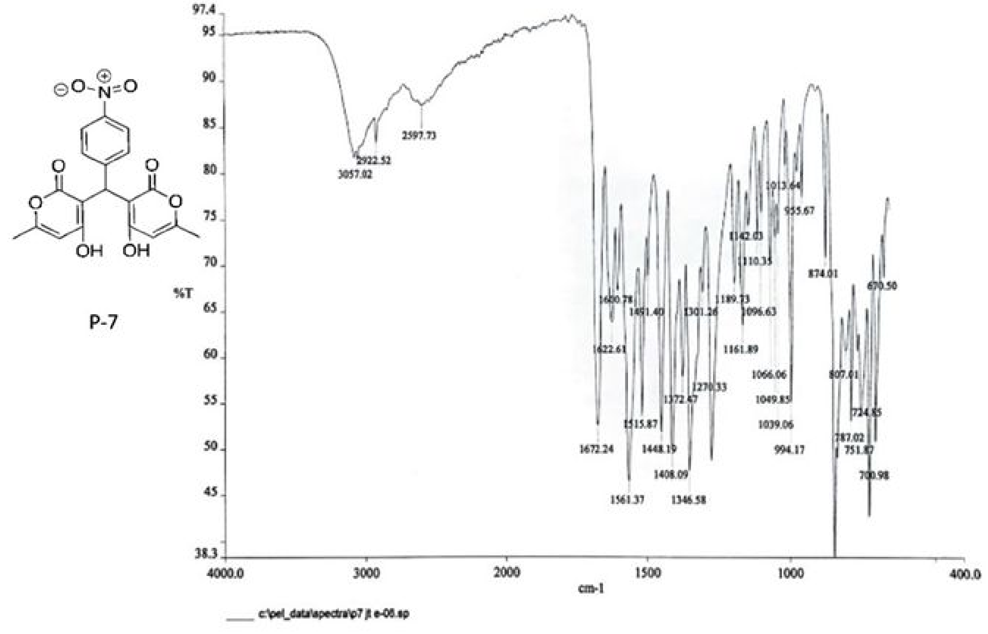

99

FigureS-99. IR spectrum of P-7.

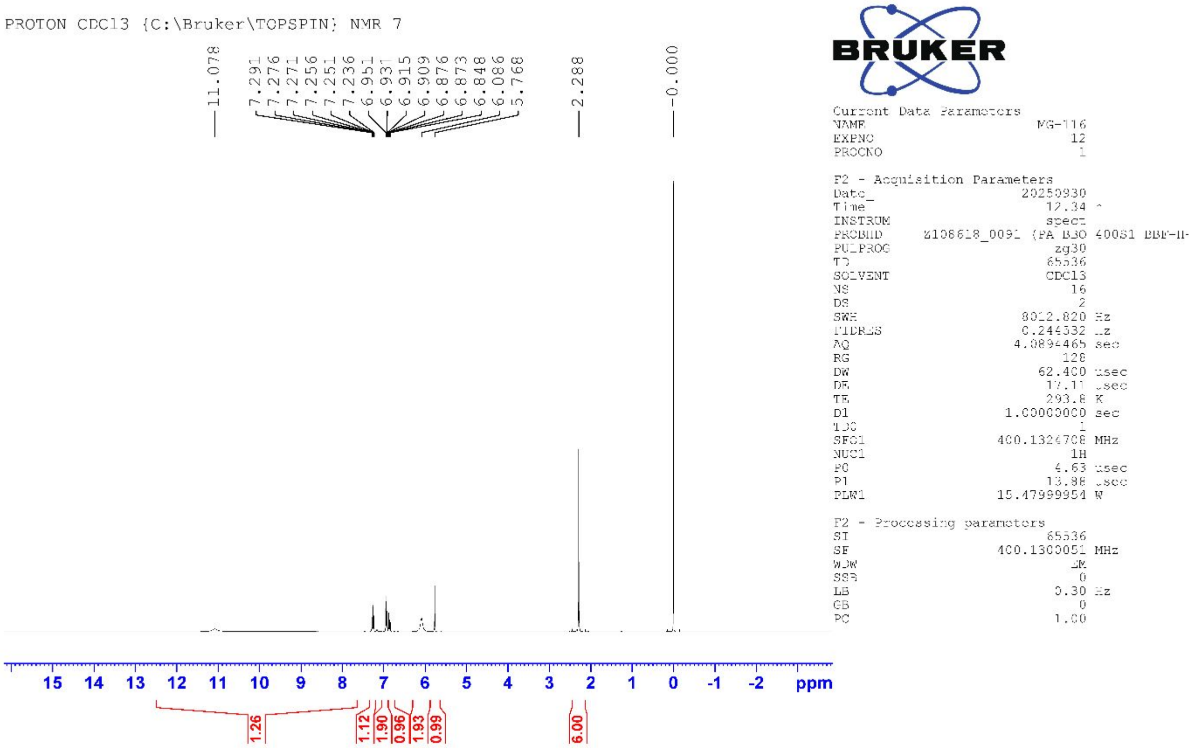

FigureS-100. **^{1}H NMR spectrum of P-8 in $CDCl_3$.**

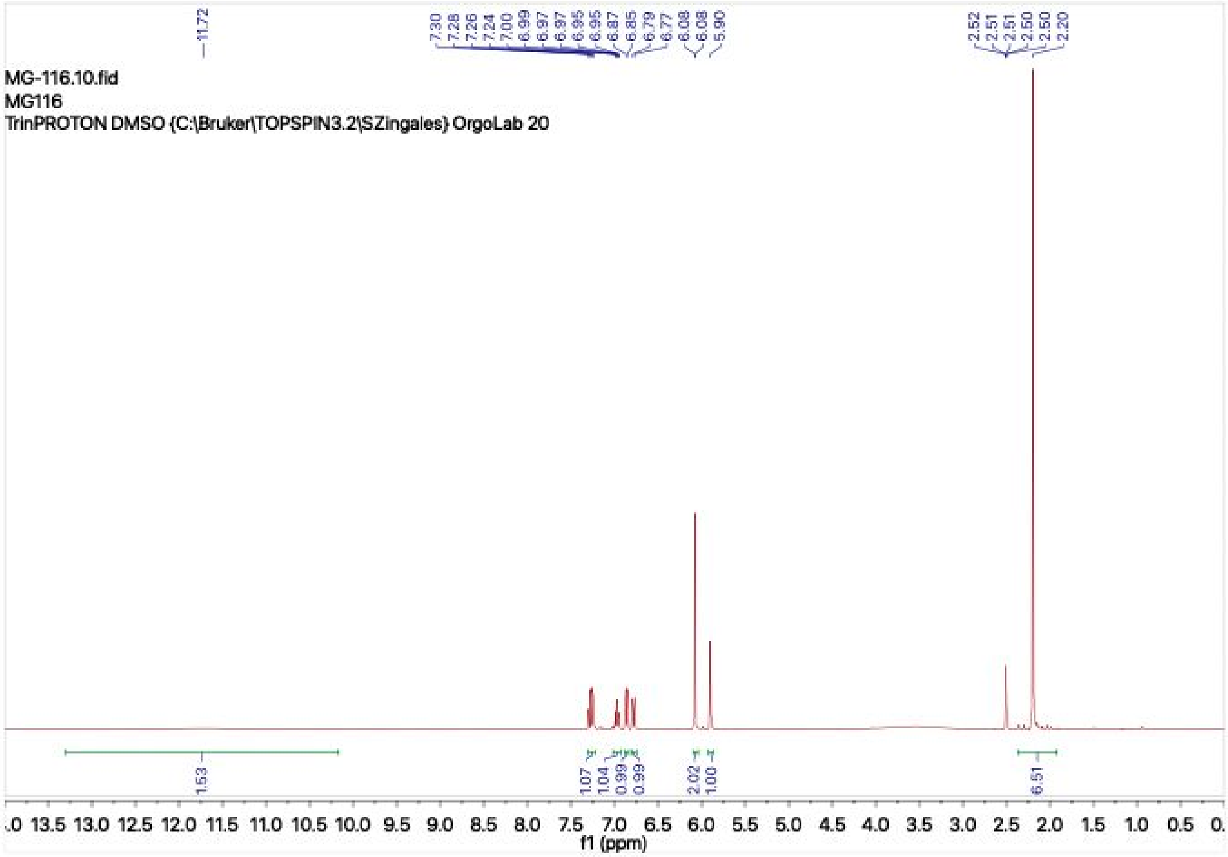

FigureS-101. **DMSO-d_6 ^{1}H NMR spectra of P-9**

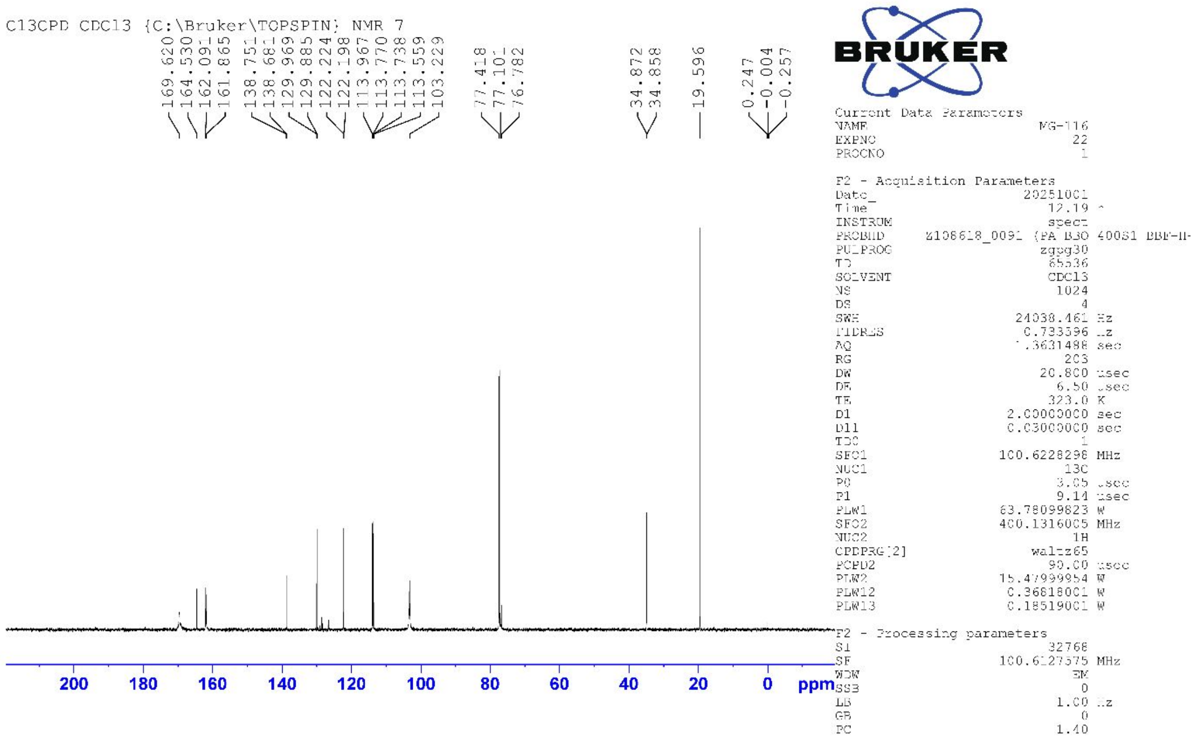

FigureS-102. **DMSO-d_6 ^{13}C NMR spectra of P-9**

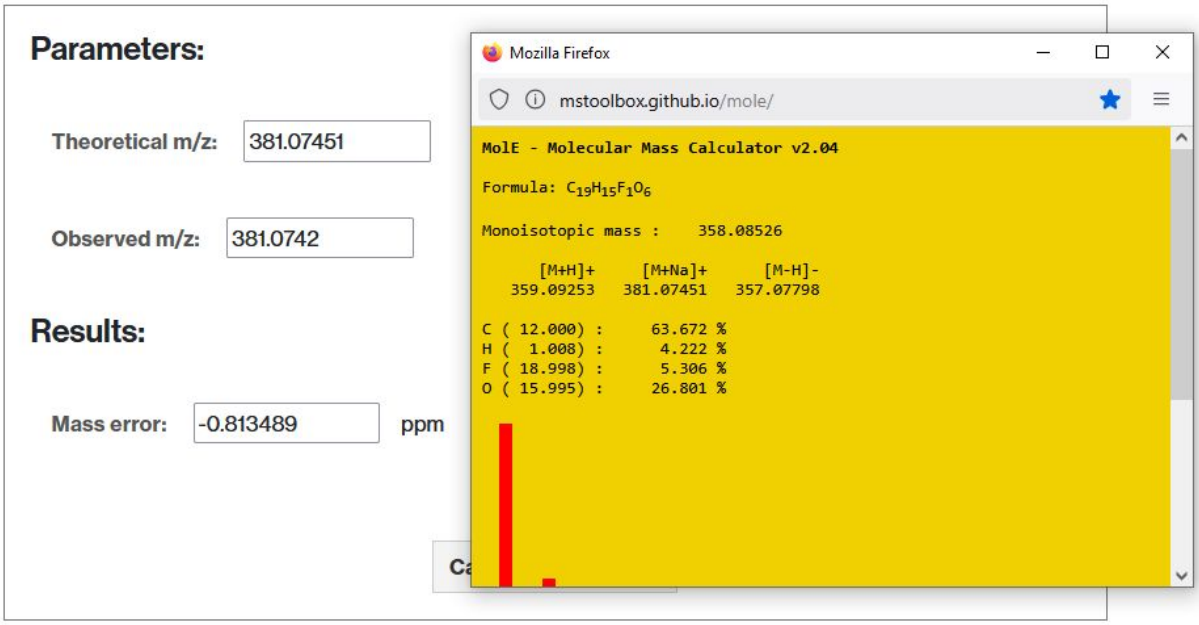

FigureS-103. **HRMS results of P-9**

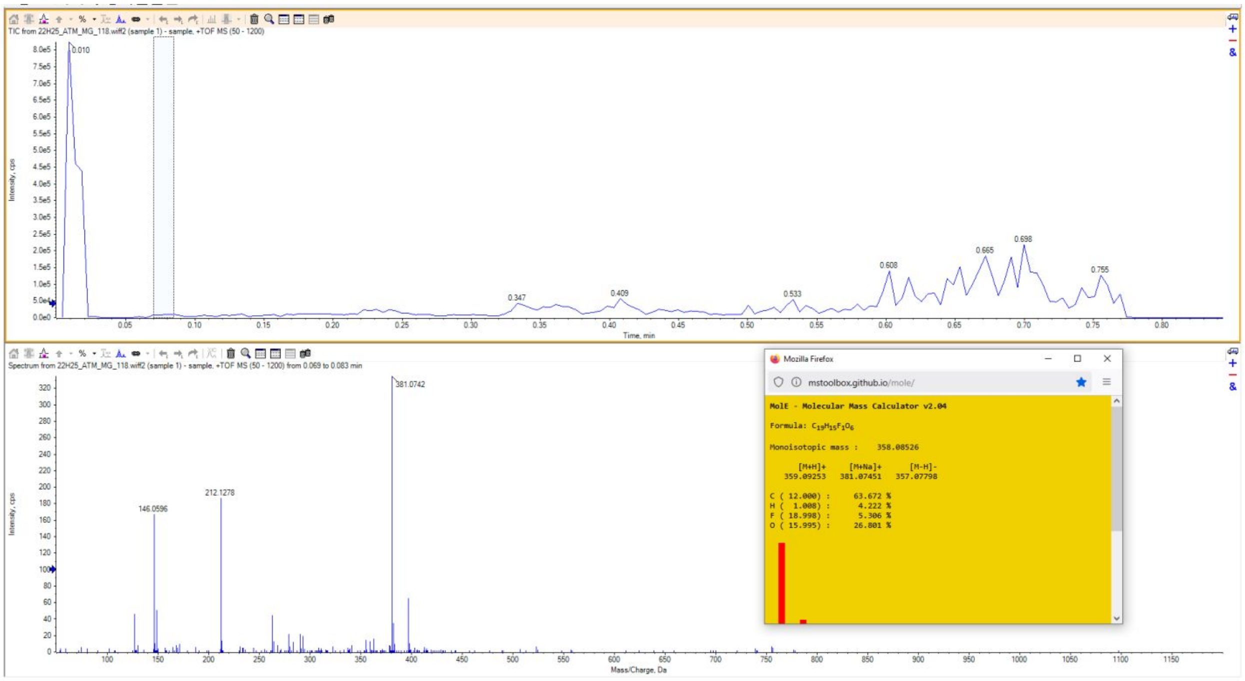

FigureS-104. **HRMS results of P-9 spectrum.**

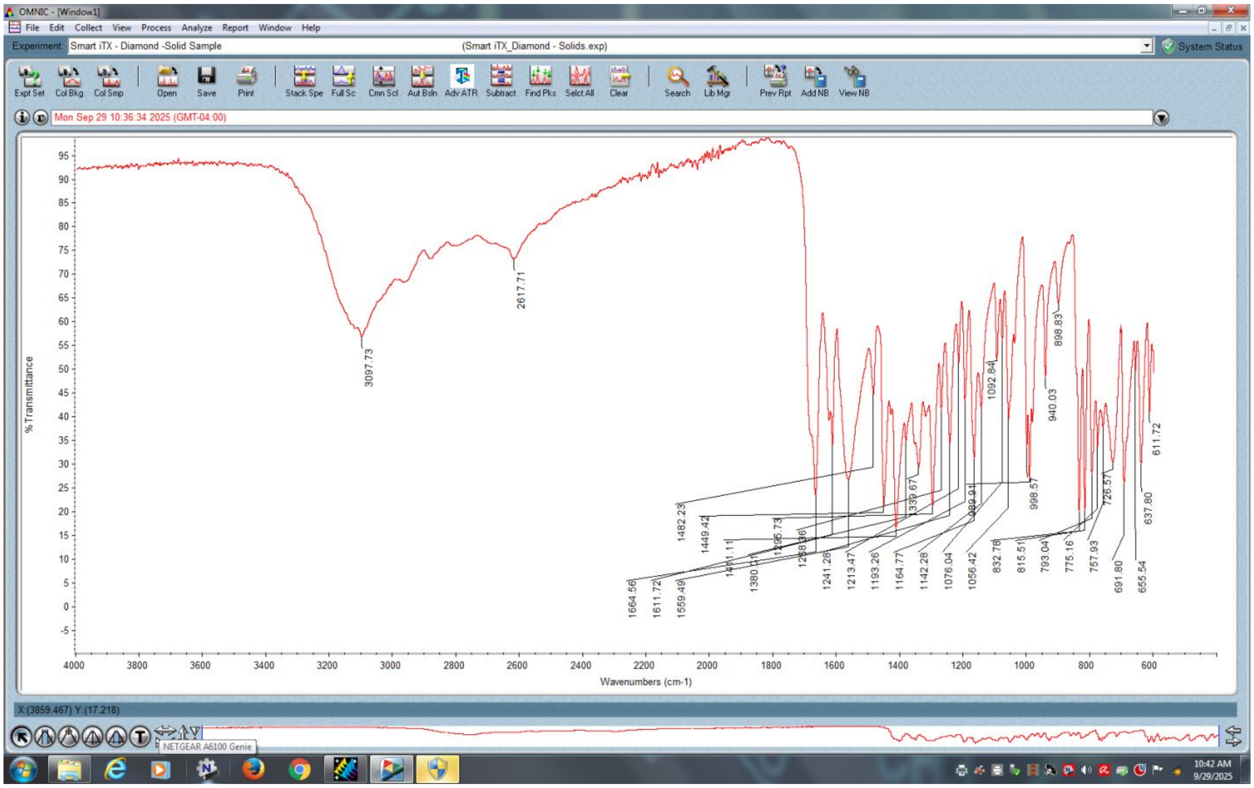

FigureS-105. **IR spectrum of P-7.**

References:

(1) Minassi, A.; Cicione, L.; Koeberle, A.; Bauer, J.; Laufer, S.; Werz, O.; Appendino, G. A Multicomponent Carba-Betti Strategy to Alkylidene Heterodimers – Total Synthesis and Structure–Activity Relationships of Arzanol. *European Journal of Organic Chemistry* **2012**, *2012* (4), 772-779. DOI: 10.1002/ejoc.201101193.
(2) Proud, M.; Sridharan, V. Iridium catalyzed acceptor-less dehydrogenative coupling of alcohols and 4-hydroxy-6-methyl-2-pyrone under microwave conditions. *Tetrahedron Letters* **2015**, *56* (47), 6614-6616. DOI: 10.1016/j.tetlet.2015.10.034.
(3) Hamdi, N.; Puerta, M. C.; Valerga, P. Synthesis, structure, antimicrobial and antioxidant investigations of dicoumarol and related compounds. *European Journal of Medicinal Chemistry* **2008**, *43* (11), 2541-2548. DOI: 10.1016/j.ejmech.2008.03.038.
(4) Zhang, X.; Qu, Y.; Fan, X.; Wang, X.; Wang, J. Ionic liquid promoted and mediated green preparation of arylbispyranylmethane and pyran derivatives and their hybrid with a pyrimidine nucleoside. *Journal of Chemical Research* **2009**, *2009* (8), 473-477. DOI: 10.3184/030823409x465321.
(5) Strashilina, I. V.; Mazhukina, O. A.; Fedotova, O. V. Variability of the Transformations of 4-Hydroxy-6-methyl-2H-pyran-2-one under Modified Biginelli Reaction Conditions. *Russian Journal of Organic Chemistry* **2018**, *54* (1), 102-106. DOI: 10.1134/S1070428018010098.
(6) Strashilina, I. V.; Arzyamova, E. M.; Fedotova, O. V. Synthesis of Fused 2H-Pyridin-2-ones under the Conditions of Multicomponent Hantzsch Reaction. *Russian Journal of Organic Chemistry* **2018**, *54* (8), 1173-1178. DOI: 10.1134/S1070428018080092.
(7) Sh, D.-Q.; Niu, L.-H.; Yao, H. An efficient synthesis of 3,3′-arylmethylenebis(4-hydroxy-6-methyl-2H-pyran-2-one)s in aqueous media. *Journal of Chemical Research* **2008**, *2008* (3), 167-169. DOI: 10.3184/030823408x304500.
(8) Zolfigol, M. A.; Navazeni, M.; Yarie, M.; Ayazi-Nasrabadi, R. Application of biological-based nano and nano magnetic catalysts in the preparation of arylbispyranylmethanes. *RSC Advances* **2016**, *6* (95), 92862-92868, 10.1039/C6RA18719F. DOI: 10.1039/C6RA18719F.
(9) Sharma, H.; Srivastava, S. Anion functionalized ionic liquid from artificial sugar: a sustainable pathway for diverse bis-enol derivatives. *New Journal of Chemistry* **2019**, *43* (30), 12054-12058, 10.1039/C9NJ01899A. DOI: 10.1039/C9NJ01899A.
(10) Elinson, M. N.; Sokolova, O. O.; Korshunov, A. D.; Barba, F.; Batanero, B. Electrocatalytic Cascade Reaction of Aldehydes and 4-Hydroxy-6-methyl-2H-pyran-2-one. *Electrocatalysis* **2018**, *9* (5), 602-607. DOI: 10.1007/s12678-018-0470-6.
